# Structural basis and evolution of the photosystem I–light-harvesting supercomplex of cryptophyte algae

**DOI:** 10.1093/plcell/koad087

**Published:** 2023-03-21

**Authors:** Long-Sheng Zhao, Peng Wang, Kang Li, Quan-Bao Zhang, Fei-Yu He, Chun-Yang Li, Hai-Nan Su, Xiu-Lan Chen, Lu-Ning Liu, Yu-Zhong Zhang

**Affiliations:** State Key Laboratory of Microbial Technology, Shandong University, Qingdao 266237, China; College of Marine Life Sciences & Frontiers Science Center for Deep Ocean Multispheres and Earth System, Ocean University of China, Qingdao 266003, China; Laboratory for Marine Biology and Biotechnology, Laoshan Laboratory, Qingdao 266237, China; College of Marine Life Sciences & Frontiers Science Center for Deep Ocean Multispheres and Earth System, Ocean University of China, Qingdao 266003, China; Laboratory for Marine Biology and Biotechnology, Laoshan Laboratory, Qingdao 266237, China; Laboratory for Marine Biology and Biotechnology, Laoshan Laboratory, Qingdao 266237, China; State Key Laboratory of Microbial Technology, Shandong University, Qingdao 266237, China; State Key Laboratory of Microbial Technology, Shandong University, Qingdao 266237, China; College of Marine Life Sciences & Frontiers Science Center for Deep Ocean Multispheres and Earth System, Ocean University of China, Qingdao 266003, China; Laboratory for Marine Biology and Biotechnology, Laoshan Laboratory, Qingdao 266237, China; State Key Laboratory of Microbial Technology, Shandong University, Qingdao 266237, China; Laboratory for Marine Biology and Biotechnology, Laoshan Laboratory, Qingdao 266237, China; State Key Laboratory of Microbial Technology, Shandong University, Qingdao 266237, China; Laboratory for Marine Biology and Biotechnology, Laoshan Laboratory, Qingdao 266237, China; College of Marine Life Sciences & Frontiers Science Center for Deep Ocean Multispheres and Earth System, Ocean University of China, Qingdao 266003, China; Institute of Systems, Molecular and Integrative Biology, University of Liverpool, Liverpool L69 7ZB, UK; College of Marine Life Sciences & Frontiers Science Center for Deep Ocean Multispheres and Earth System, Ocean University of China, Qingdao 266003, China; Laboratory for Marine Biology and Biotechnology, Laoshan Laboratory, Qingdao 266237, China; State Key Laboratory of Microbial Technology, Marine Biotechnology Research Center, Shandong University, Qingdao 266237, China

## Abstract

Cryptophyte plastids originated from a red algal ancestor through secondary endosymbiosis. Cryptophyte photosystem I (PSI) associates with transmembrane alloxanthin-chlorophyll *a*/*c* proteins (ACPIs) as light-harvesting complexes (LHCs). Here, we report the structure of the photosynthetic PSI–ACPI supercomplex from the cryptophyte *Chroomonas placoidea* at 2.7-Å resolution obtained by crygenic electron microscopy. Cryptophyte PSI–ACPI represents a unique PSI–LHCI intermediate in the evolution from red algal to diatom PSI–LHCI. The PSI–ACPI supercomplex is composed of a monomeric PSI core containing 14 subunits, 12 of which originated in red algae, 1 diatom PsaR homolog, and an additional peptide. The PSI core is surrounded by 14 ACPI subunits that form 2 antenna layers: an inner layer with 11 ACPIs surrounding the PSI core and an outer layer containing 3 ACPIs. A pigment-binding subunit that is not present in any other previously characterized PSI–LHCI complexes, ACPI-S, mediates the association and energy transfer between the outer and inner ACPIs. The extensive pigment network of PSI–ACPI ensures efficient light harvesting, energy transfer, and dissipation. Overall, the PSI–LHCI structure identified in this study provides a framework for delineating the mechanisms of energy transfer in cryptophyte PSI–LHCI and for understanding the evolution of photosynthesis in the red lineage, which occurred via secondary endosymbiosis.

## Teaser

Structural analysis of the photosystem I—light-harvesting complex of cryptophyte algae provides insights into light harvesting, energy transfer, and the evolution of photosynthesis in the red lineage.

**IN A NUTSHELL**

**Background:** Photosynthesis converts solar energy into biologically useful energy and generates oxygen, sustaining almost all life forms on Eartbh. Photosystem I (PSI) is a large pigment-protein supercomplex that plays a key role in photosynthesis. High-resolution structural analysis of the PSI and its antenna complexes is crucial for elucidating the mechanism of light capture and energy transfer in photoautotrophs. So far, the PSI structures of cyanobacteria, red algae, green algae, diatom, moss, and land plants have been resolved, providing insights into the energy conversion mechanisms and important clues for the evolutionary diversity of PSI–LHCI structures.

**Question:** Cryptophytes are an ancient group that originated from red algae during secondary endosymbiosis. They possess unique evolutionary traits and play a crucial role in ecology. Despite their importance in ecological function and evolution, the high-resolution structure of cryptophyte PSI remains elusive.

**Findings:** The PSI–light-harvesting antenna (LHCI) supercomplex was isolated from a cryptophyte *Chroomonas placoidea*, and its structure was determined by cryo-EM. Cryptophyte PSI is composed of 14 core subunits, 14 LHCIs surrounding the PSI core and a pigment-binding polypeptide not present in any other reported PSI–LHCI complexes. The structure coordinates a total of 373 pigments, which facilitate efficient capturing of light and energy transfer from LHCIs to the PSI core. Cryptophyte PSI–LHCI shares common structural features with both red algal and diatom counterparts, while also displaying unique protein organization, pigment association, and energy transfer pathways. By uncovering these structural variations, our study sheds light on the unique features of cryptophyte PSI–LHCI as an intermediate state during the evolution of red lineage PSI–LHCI.

**Next steps:** Our cryo-EM structure reveals an intense pigment network within cryptophyte PSI–LHCI. Comprehensive and accurate analysis of energy transfer pathways in cryptophyte PSI–LHCI relative to the PSI–LHCI from other photoautotrophs needs to be performed experimentally and theoretically. Moreover, uncovering the PSI–LHCI structures of various species is critical to understanding the structural diversity of PSI–LHCI during evolution and environmental adaptation.

## Introduction

Oxygenic photosynthesis, one of the most important types of metabolism that produces atmospheric oxygen and organic matter, plays a fundamental role in driving evolution (Dekker and Boekema 2005; Nelson and Junge 2015). Through oxygenic photosynthesis, cyanobacteria, algae, and plants capture sunlight energy and convert it into chemical energy to drive almost all life activities. The major components responsible for light-driven photosynthetic electron transport are photosystem I (PSI) and photosystem II (PSII); these multi-subunit pigment-protein supercomplexes reside in thylakoid membranes (Mullineaux and Liu 2020). PSI transfers electrons derived from the PSII-mediated oxidation of water to ferredoxin, producing reducing power and the energy needed for CO_2_ assimilation. The PSI reaction center core is generally surrounded by light-harvesting complexes (LHCs), which bind with numerous pigments including chlorophylls (Chls) and carotenoids, forming a PSI−LHCI supercomplex to increase the absorption cross-section of PSI (Nelson and Junge 2015; Qin et al. 2015, 2019; Mazor et al. 2017; Pi et al. 2018; Antoshvili et al. 2019; Su et al. 2019; Suga et al. 2019; Caspy et al. 2020; Nagao et al. 2020; Xu et al. 2020; Yan et al. 2021; Gorski et al. 2022; Naschberger, Fadeeva, et al. 2022; Naschberger, Mosebach, et al. 2022).

Cryptophytes are a phylum of single-celled biflagellate eukaryotic algae that function as significant primary producers in ecologically diverse habitats (Shalchian-Tabrizi et al. 2008; Stiller et al. 2014; Zimorski et al. 2014; Kim et al. 2017; Abidizadegan et al. 2021). In parallel with the evolution of green lineages, cryptophytes and diatoms obtained the specialized photosynthetic organelles plastids from an ancestral red alga via secondary endosymbiosis. Whereas red algae contain extrinsic antenna phycobilisomes and transmembrane (TM) LHCs but lack Chl *c*, and diatoms possess Chl *a/c*-containing LHCs but lack phycobiliproteins, cryptophytes utilize both phycobiliproteins and LHCs, which contain Chl *a*/*c*_2_ as well as alloxanthins as the major carotenoids, thereby designated alloxanthin Chl *a*/*c*-binding proteins (ACPIs). ACPIs play an role in extending the spectral region of captured light (Pennington et al. 1985; Schagerl and Donabaum 2003; Chen et al. 2007; Janssen and Rhiel 2008; Kereiche et al. 2008; Takaichi 2011; Takaichi et al. 2016; Greenwold et al. 2019), pointing to their unique position in the evolution of red-lineage plastids. The structures of PSI–LHCI supercomplexes in red algae and diatoms, known as PSI–LHCR and PSI–FCPI (fucoxanthin-chlorophyll *a*/*c*-binding proteins), respectively, have recently been solved (Pi et al. 2018; Antoshvili et al. 2019; Nagao et al. 2020; Xu et al. 2020). However, the architecture and the pigment arrangement of cryptophyte PS−LHCI supercomplexes remain unclear.

Here, we report the cryogenic electron microscopy (cryo-EM) structure of PSI–ACPI, an 885-kDa membrane-spanning photosynthetic protein supercomplex, from the cryptophyte *Chroomonas placoidea* at 2.7-Å resolution. The structure reveals that PSI–ACPI comprises 14 ACPI subunits that form 2 antenna layers surrounding a PSI core containing a total of 373 pigments. In addition, 2 protein subunits were identified that are not present in other previously characterized PSI–LHCI complexes. Our study provides important insight into the molecular mechanisms of light capturing and energy transfer in cryptophyte PSI–ACPI. In addition, we provide evidence for the evolutionary variations of PSI–LHCI supercomplexes in the red lineage.

## Results and discussion

### Overall architecture of the PSI–ACPI supercomplex

**Figure 1.**
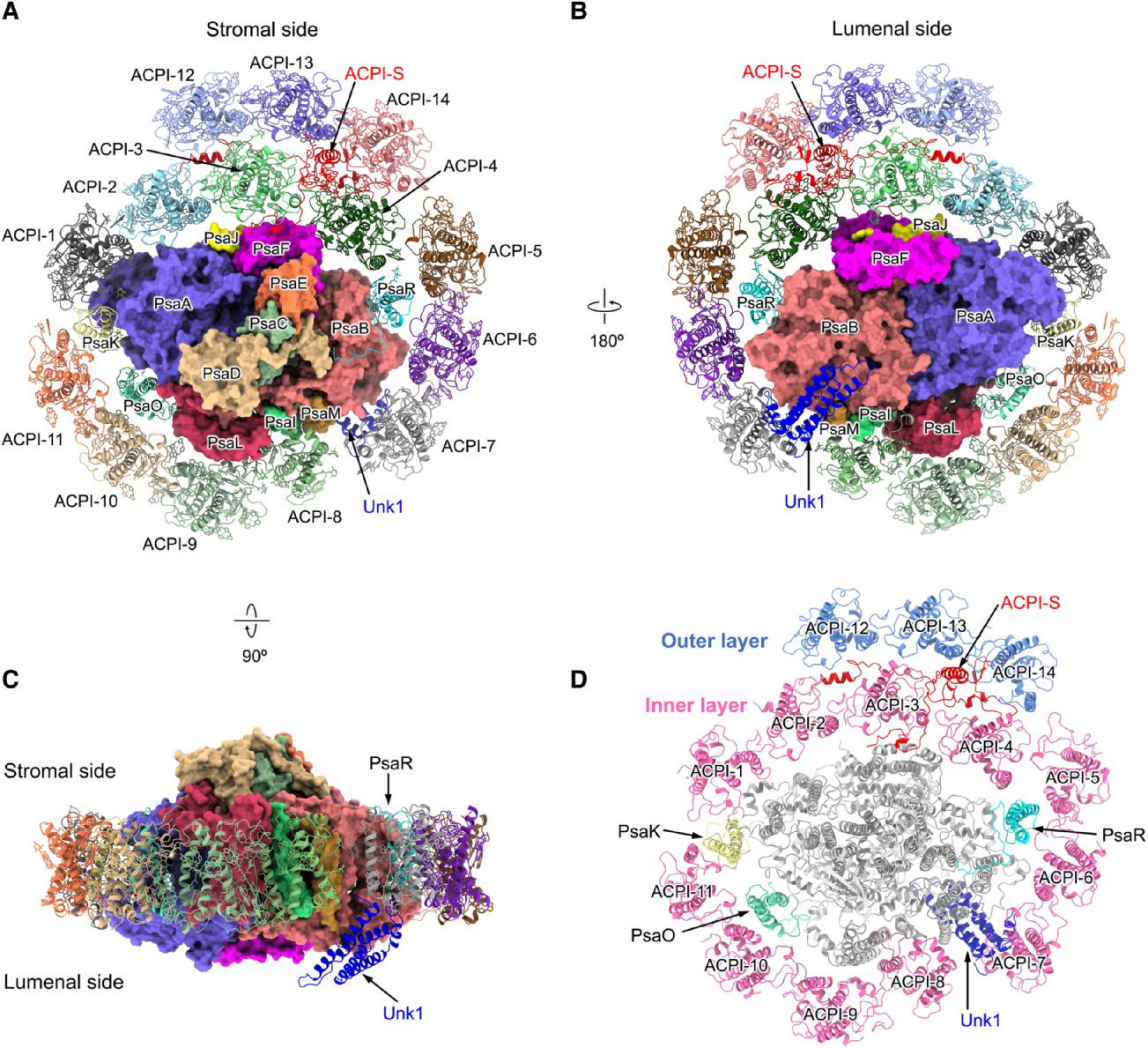
Overall structure of the cryptophyte PSI–ACPI supercomplex. **A)** The PSI–ACPI supercomplex viewed from the stromal side. Core subunits are shown in surface view and their names labeled. LHCs are shown in cartoon form and labeled as ACPI. The PSI core subunit and the subunit lying between the ACPs are labeled “Unk1” and “ACPI-S”, respectively. **B)** The PSI–ACPI supercomplex viewed from the lumenal side. **C)** Side view of the PSI–ACPI supercomplex. **D)** Two layers of ACPIs in the PSI–ACPI supercomplex. Outer layer, pink; the PSI core, gray; ACPI-S and Unk1 are shown in red and blue, respectively.

We isolated the PSI–ACPI supercomplex from *C. placoidea* (Supplemental Fig. S1) and determined its structure by cryo-EM (Supplemental Fig. S2 and Table S1). Two distinct classes of PSI–ACPI were identified: 1 with 14 ACPI subunits (2.66 Å resolution) and 1 with 11 ACPI subunits (2.71 Å resolution), possessing similar B-factors (Supplemental Fig. S3 and Table S1). Apart from the 3 additional ACPIs (see details below), the remaining structures of the 2 classes of PSI–ACPI are essentially identical (Supplemental Fig. S4). The PSI–ACPI supercomplex has a dimension of approximately 205 × 195 × 110 Å^3^ and a molecular mass of approximately 885 kDa. This supercomplex comprises 1 PSI core surrounded by 14 ACPI subunits and 2 subunits that are not present in other supercomplexes that were previously examined, ACPI-S and unknown protein 1 (Unk1) (Fig. 1, A and C). ACPI-S separates the 14 ACPIs into 2 layers: an inner antenna layer around the PSI core comprising ACPI-1 to ACPI-11; and an outer antenna layer comprising ACPI-12 to ACPI-14 (Fig. 1D). We also identified 254 Chl *a*, 20 Chl *c*, 59 alloxanthin (Alx), 25 α-carotene (α-Car), 12 crocoxanthin (Cro), 3 monadoxanthin (Mon), and 2 phylloquinone molecules, 3 Fe_4_S_4_ clusters, 5 detergent molecules, as well as 34 lipids including 23 phosphatidyl glycerol (PG), 7 monogalactosyldiacyl glycerol, 2 digalactosyldiacyl glycerol, and 2 sulfoquinovosyldiacyl glycerol molecules (Supplemental Fig. S5 and Table S2). The ratios of Chl *a*, Chl *c*, and Alx are consistent with those of the purified samples determined by high-performance liquid chromatography (HPLC) (Supplemental Fig. S1E).

### Structure of the PSI core

The cryptophyte PSI core consists of 14 subunits (PsaA–F, PsaI–M, PsaO, PsaR) and the subunit Unk1, which is not present in other previously characterized PSI complexes (Fig. 1; Supplemental Table S3). PsaA/B/C/D/E/F/I/J/L are conserved in all oxyphototrophs. In contrast, PsaK is absent in diatoms, PsaM is absent in plants, PsaO is absent in cyanobacteria and diatoms, and PsaR was recently identified in the diatom PSI–LHCI (Supplemental Table S3) (Jordan et al. 2001; Qin et al. 2015, 2019; Mazor et al. 2017; Pi et al. 2018; Antoshvili et al. 2019; Su et al. 2019; Suga et al. 2019; Nagao et al. 2020; Xu et al. 2020; Yan et al. 2021; Gorski et al. 2022; Kato et al. 2022). Moreover, the cryptophyte PSI core lacks the PsaG/H/N subunits, which are unique in green-lineage organisms and PsaX, which is unique in cyanobacteria (Jordan et al. 2001; Qin et al. 2015, 2019; Mazor et al. 2017; Su et al. 2019; Yan et al. 2021; Gorski et al. 2022).

The PSI core contains 100 Chl *a*, 19 α-Car, 5 Alx (A850, B843, O204, O205, R203), and 2 Cro (K104, L204) molecules (Supplemental Table S2). The cryptophyte PSI core possesses all the Chl-binding sites identified in the red algal and diatom PSI cores (Supplemental Fig. S6). A new Chl-binding sites (PsaB/a841) were found in the cryptophyte PSI core, which is absent in the red algal and diatom PSI cores (Supplemental Fig. S6, B and E). The cryptophyte PSI core lacks 1 carotenoid-binding site identified in red algal PsaA, as does the diatom PSI core. However, 6 new carotenoid-binding sites evolved in the PsaJ/M/O/R subunits compared with the red algal PSI core, 3 of which were identified in PsaJ and PsaO but are also absent in the diatom PSI core (Supplemental Fig. S6, C and F). Alx and Cro replace α-Cars or β-Cars in the PSI cores of other oxyphototrophs (Supplemental Fig. S6F), which is similar to the diatom PSI core, in which several β-Cars are replaced by Ddx and Fx (Xu et al. 2020).

**Figure 2.**
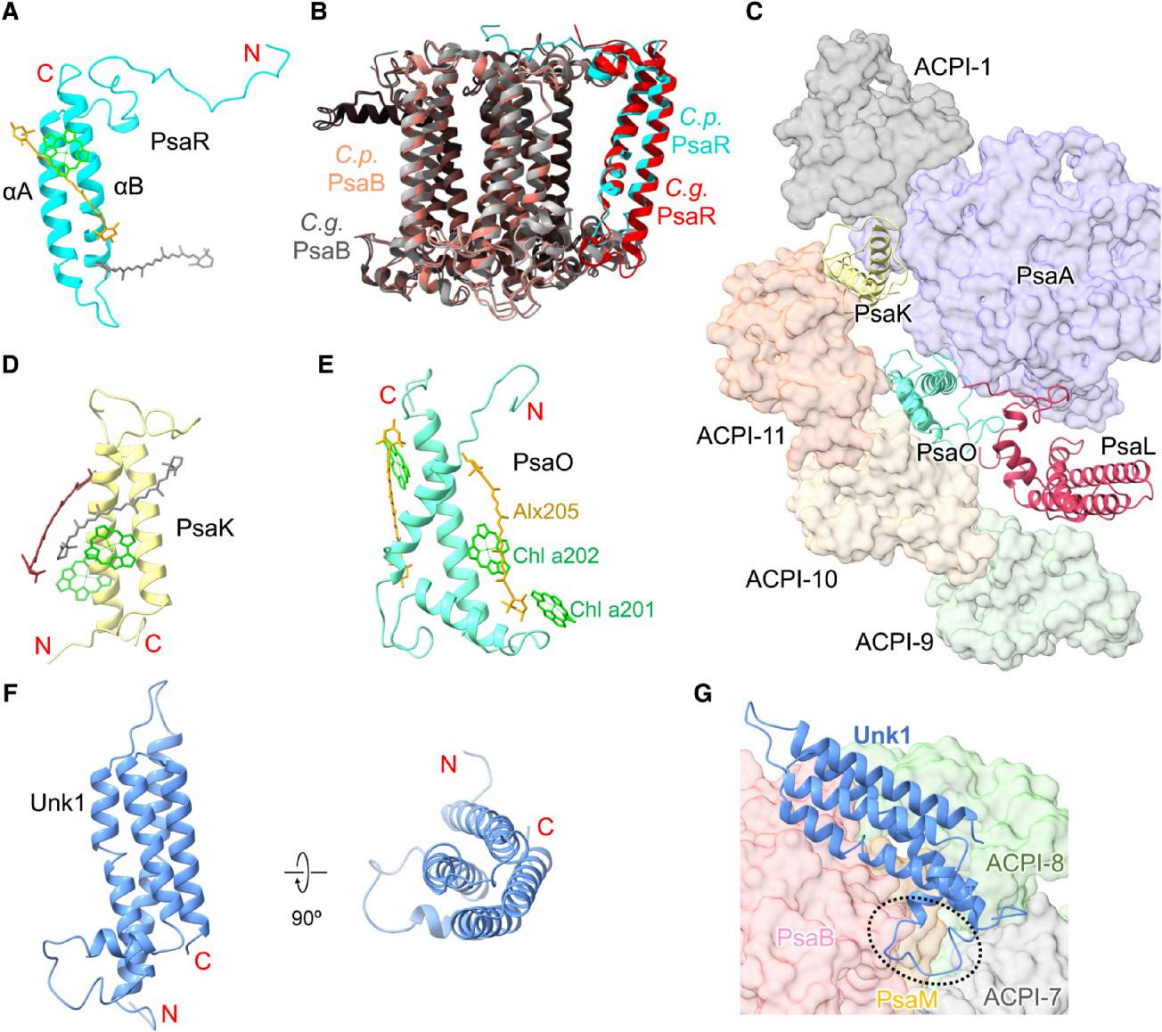
Locations and structures of the subunits PsaR, PsaK, PsaO, and Unk1. **A)** The structure of PsaR. The 2 TM helices are labeled as αA and αB. **B)** Comparison of the locations of PsaR in *C. placoidea* PSI and PsaR in *C. gracilis* PSI. **C)** The locations of PsaK and PsaO in *C. placoidea* PSI viewed from the stromal side. **D)** The structure of PsaK. **E)** The structure of PsaO. **F)** The structure of Unk1. **G)** The binding site of Unk1 in the PSI core, as viewed from the lumenal side. Color code for pigments: Chl *a*, green; α-Car, gray; Alx, orange; Cro, brown.

Cryptophyte PsaR binds to 1 Chl *a* and 2 carotenoids and shares a high similarity with diatom PsaR in terms of amino acid sequence, structure, and location within PSI–LHCI (Fig. 2, A and B; Supplemental Figs S7A and S8B); both have low sequence similarity to PsaG of green-lineage organisms (Xu et al. 2020). In addition, cryptophyte PsaR possesses a longer N-terminal loop at the stromal side, a shorter helix αA at the lumenal side, and a shorter loop between αA and αB relative to diatom PsaR, which may enhance its binding with PsaB (Supplemental Fig. S8, A and D). Like diatom PsaR (Xu et al. 2020), cryptophyte PsaR also mediates the association of peripheral antenna components and energy transfer (Supplemental Fig. S8, E to G). Cryptophyte PsaK and PsaO are highly similar to their counterparts located at the same positions in the red algal PSI core (Pi et al. 2018; Antoshvili et al. 2019) (Fig. 2, C to E; Supplemental Figs S6A, S7, C, D, and S9, A and E). PsaK binds to 2 Chl *a* and 2 carotenoids and mediates the binding of the PSI core with ACPI-1 and ACPI-11 via its long loop at the stromal side, which is absent in red algal PsaK (Fig. 2C; Supplemental Fig. S9, A to C). The extended N-terminal loop of cryptophyte PsaK facilitates its association with PsaA (Supplemental Fig. S9, A and D). PsaO binds to 3 Chl *a* and 2 Alx molecules, and a new carotenoid-binding site (Alx205) was identified close to Chl *a*201/202 (Fig. 2E), suggesting its role in energy quenching. The extended terminal loops of PsaO and PsaL enhance the association of PsaO with the core (Supplemental Fig. S9, E to I). Although PsaK and PsaO coexist in red algae, green algae, and plants (Qin et al. 2015, 2019; Mazor et al. 2017; Pi et al. 2018; Antoshvili et al. 2019; Su et al. 2019; Caspy et al. 2020; Yan et al. 2021; Gorski et al. 2022), the PsaK/O-mediated binding of antennas to the PSI core at the PsaK–PsaO side was only identified in cryptophyte PSI–ACPI (Supplemental Fig. S10).

A distinctive feature of the cryptophyte PSI core is the presence of a extrinsic subunit Unk1 at the lumenal side close to PsaB, PsaM, and ACPI-7 (Figs 1 and 2, F and G). Its amino acid residues could not be assigned due to its low-resolution density map, and its structure was built as poly-alanines. Unk1 lacks ligands and contains 4 parallel α-helices close to the lumenal side of PsaB (Fig. 2, F and G). Its long loop between helices binds to the interface of PsaB, PsaM, and ACPI-7 (Fig. 2G). The low-resolution densities suggest the loose association of Unk1 with the PSI core. We found that the densities assigned to Unk1 do not fit the structures of the cryptophyte phycocyanin β subunit (PDB: 4LMS), PsbQ (PDB: 3LS0), Psb27 (PDB: 2Y6X), or the PsnL2 or PsnL3 subunits of the NDH-1 complex (PDB: 7WFF), which all possess 4 parallel α-helices. The exact structure and role of Unk1 merit further investigation.

### Structures of ACPI subunits

**Figure 3.**
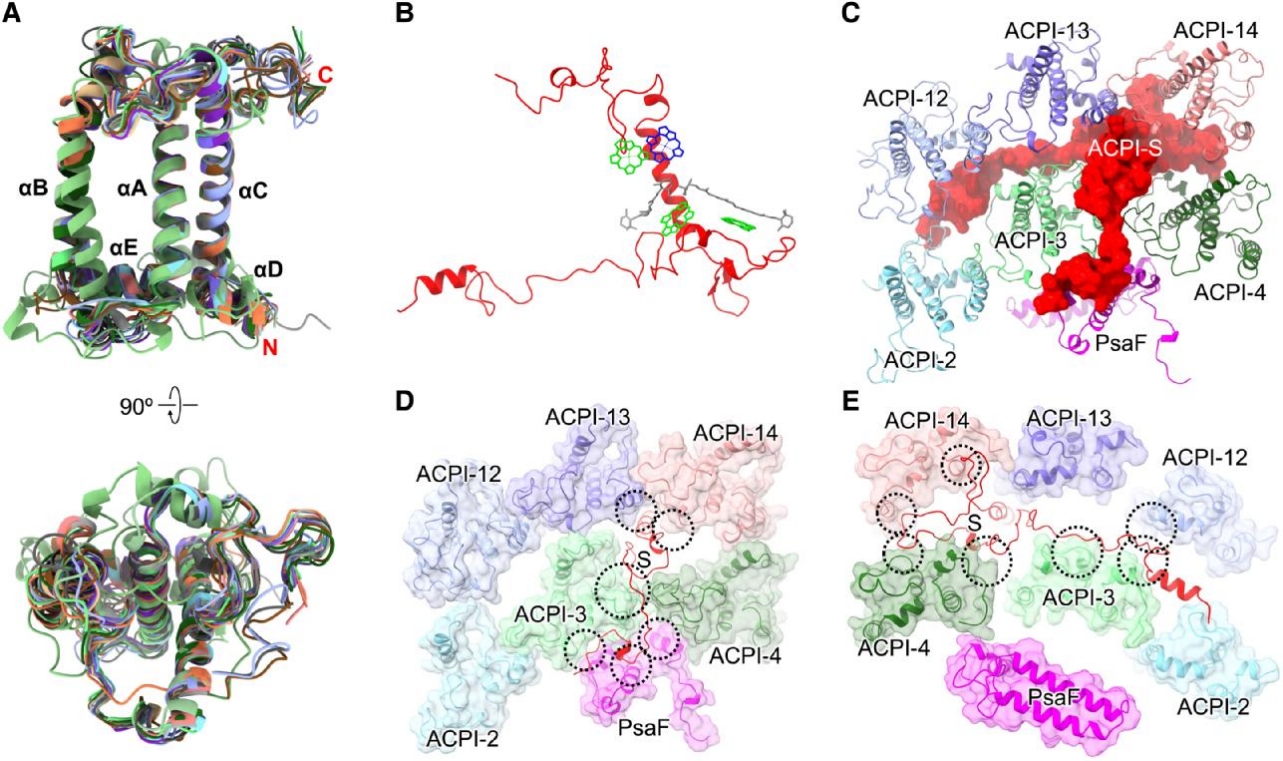
Structures of ACPI subunits and location of ACPI-S. **A)** Structural comparison of 14 ACPIs. **B)** The structure of ACPI-S. Color code for pigments: Chl *a*, green; Chl *c*, blue; α-Car, gray. **C)** Location of ACPI-S (displayed in surface view from the stromal side). Interactions between ACPI-S and the surrounding subunits at the stromal side **D)** and lumenal side **E)** indicated by dashed circles. Detailed analyses are shown in Supplemental Fig. S14.

All ACPI apoproteins contain 3 major TM helices (αA, αB, αC) and a short amphipathic helix αE between αA and αB, and 11 ACPIs (2 to 4, 6 to 11, 13 to 14) possess an amphipathic helix αD at the C-terminal region (Fig. 3A; Supplemental Figs S11 and S12), resembling those of other LHCs (Qin et al. 2015, 2019; Mazor et al. 2017; Pi et al. 2018; Antoshvili et al. 2019; Su et al. 2019; Suga et al. 2019; Caspy et al. 2020; Nagao et al. 2020; Xu et al. 2020; Yan et al. 2021; Gorski et al. 2022). The C-termini of ACPI-1/5/12 are shorter and that of ACPI-9 is longer than those of other ACPIs (Supplemental Figs S11 and S12). Since the cryo-EM densities of the ACPI-10 and ACPI-13 subunits are identical, we used the same sequence from the transcriptome to build their models. To better illustrate the structure, we assigned them ACPI-10 and ACPI-13 based on their distinct positions and binding sites in the PSI–ACPI supercomplex (Fig. 1A). Two highly conserved Glu-Arg pairs form salt bridges between the αA and αC helices in all ACPIs to stabilize the ACPI structure (Supplemental Fig. S12).

The ACPI-S subunit has a molecular mass of 20.6 kDa and is located between ACPI-2/3/4 and ACPI-12/13/14; it binds to 3 Chls *a*, 1 Chl *c*, and 2 α-Car molecules, thereby presumably serving as a special antenna subunit (Fig. 3, B and C; Supplemental Fig. S13A). An ACPI-S homolog was only found in one other cryptophyte species, *Guillardia theta* (Supplemental Fig. S7E). ACPI-S has 1 TM helix at the interface between ACPI-3, ACPI-4, ACPI-13, and ACPI-14, as well as long-terminal loops and an amphipathic helix at the C-terminal region parallel to the lumenal membrane surface. Its structure is very different from that of other ACPIs: its TM helix is shorter than those of other ACPIs, and its coordinated Chls have distinct binding positions from those of other ACPIs (Supplemental Fig. S13B). The overall structure of ACPI-S stretches across ACPI-2/3/4/12/13/14 via its long N- and C-terminal loops, forming interactions with PsaF, ACPI-3/4, and ACPI-12/13/14 and resulting in slight shifts of ACPI-4 and ACPI-5 in PSI–ACPI (relative to FCPI-8 and FCPI-9 in diatom PSI–FCPI) (Fig. 3, D and E; Supplemental Fig. S13, C, D, and S14).

Phylogenic analysis revealed that ACPIs belong to the Lhcr family, similar to red algal LHCRs and diatom Lhcr-type FCPIs, except that the ACPI-8 subunit forms a group with FCPI-1 (Supplemental Fig. S15). Lhcr-type ACPIs can be categorized into Group I (ACPI-2/3/4/7/11/14) and Group II (ACPI-1/5/6/9/10/12/13), which mainly differ in the structures of their AE loops between αA and αE (Supplemental Fig. S16, A and B). ACPI-3/7/11/14 in Group I share similar structures containing a short αB helix, which is consistent with the structures of red algal Lhcr1 and diatom FCPI-7 (Supplemental Fig. S16A). ACPI-1/2 structurally resemble the corresponding red algal Lhcrs (Lhcr3/2). However, the structures of ACPI-1 and its counterpart diatom FCPI-5 differ in both the loop and helix regions due to the loss of PsaK in diatom PSI–FCPI (Supplemental Fig. S16B) (Xu et al. 2020). ACPI-4 shows some structural differences with its diatom counterpart FCPI-8, especially in the loop regions, facilitating its interactions with adjacent subunits (Supplemental Figs S13, C, D, S16A, and S17A). ACPI-S and FCPI-19 share similar positions within the supercomplexes, whereas the TM helix of ACPI-S is further from the PSI core than the TM helix αB of FCPI-19 (Supplemental Fig. S13D). These differences may result in the shifting of ACPI-4 compared with FCPI-8. ACPI-5 shifts relative to its diatom counterpart FCPI-9 and differs from FCPI-9 in its loop regions, particularly the N-terminal loop (Supplemental Fig. S16B), which favors the binding of FCPI-21, a region with no homolog in cryptophyte PSI–ACPI (Xu et al. 2020). ACPI-7 has a longer C-terminal loop than red algal Lhcr2* and diatom FCPI-11 at the same position, which helps stabilize the binding of ACPI-7 (Supplemental Figs S16A and S17B). The structure of ACPI-8 is markedly distinct from those of other ACPIs and red algal Lhcr1* but is comparable with its counterpart FCPI-1 in diatoms (Supplemental Figs S16C and S18, A and D) (Pi et al. 2018; Xu et al. 2020). ACPI-8 also possesses a unique N-terminal helix, which is absent in diatom FCPI-1 (Supplemental Fig. S18D).

Intriguingly, the 4 groups of 3 adjacent ACPIs (ACPI-1/2/3, ACPI-5/6/7, ACPI-9/10/11, and ACPI-12/13/14) show high conformational similarity, pointing to their structural coordination within the PSI–ACPI supercomplex (Supplemental Fig. S19). Similar LHC trimers were also found in red algal PSI–LHCR (Lhcr-3/2/1, which has the same binding position with the ACPI-1/2/3 trimer) and diatom PSI–FCPI (FCPI-5/6/7 relative to the ACPI-1/2/3 trimer and FCPI-9/10/11 relative to ACPI-5/6/7) (Supplemental Fig. S20, A and E). These trimers may had served as LHC building modules during the expansion of LHCs in the evolution of red lineage PSI–LHCI. These trimers directly associate with the PSI core, which is assumed to facilitate the stabilization of LHC trimer organization.

### Associations between the PSI core and ACPIs

The associations between adjacent ACPIs within the same layer are mainly formed by the BC loop (between Αb and Αc) of ACPI-*n* and the N-terminal loop of ACPI-(*n* + 1), except for ACPI-7/8 and ACPI-11/1, which have greater inter-subunit distances (Supplemental Fig. S21). The interactions between outer and inner ACPIs are mainly mediated by ACPI-S via their loops at both membrane surfaces (Supplemental Figs S13 and S14). An ACPI-S homolog is absent in diatom PSI–FCPI, and diatom FCPI-17/18/19/20 (which share similar positions with the outer ACPI-12/13/14 subunits) could readily be detached from the inner FCPI ring (Nagao et al. 2020; Xu et al. 2020), further suggesting the important role of ACPI-S in stabilizing ACPIs. In addition, the outer ACPI BC loops and N-terminal loops also form interactions with the BC loops of inner ACPIs, reinforcing inter-layer interactions (Supplemental Fig. S21).

The inner ACPIs associate with the PSI core mainly via their loop regions, as do LHCIs from other oxyphototrophic species (Supplemental Fig. S21). The ACPI-1/2/3/8 subunits have similar binding sites with the PSI core as their counterparts in red algal PSI–LHCR and diatom PSI–FCPI (Supplemental Fig. S20, A and E; Movie 1) (Pi et al. 2018; Xu et al. 2020). ACPI-8 binds with the PSI core via its AB loop (Supplemental Fig. S17, C and E), which is consistent with diatom FCPI-1 (Xu et al. 2020). ACPI-4/5/6 bind to the PSI core via PsaR, as observed in diatom PSI–FCPI (Supplemental Figs S8, E to G and S20E) (Nagao et al. 2020; Xu et al. 2020). The Unk1 subunit mediates the interactions between ACPI-7 and PsaB, resulting in the shift of ACPI-7 outward from the PSI core compared with Lhcr2* and FCPI-11 (Supplemental Fig. S20, C, D, I, and J; Movie 1 and 2). Compared with their FCPI counterparts, the locations of ACPI-9/10/11/12/13/14 are greatly shifted, presumably due to the presence of ACPI-S in cryptophyte PSI–ACPI and the absence of PsaK and PsaO in PSI–FCPI (Supplemental Fig. S20, G and H; Movie 1). Apart from the finding that the PSI–ACPI supercomplex possesses 14 ACPI subunits, we also observed 1 class of PSI–ACPI that contains 11 ACPIs but not ACPI-9/10/11, suggesting relatively weak associations of ACPI-9/10/11 to the PSI core (Supplemental Figs S2 to S4).

### Pigment arrangement in ACPIs

The 14 ACPIs contain 151 Chl *a*, 19 Chl *c*, 54 Alx, 10 Cro, 3 Mon, and 4 α-Car molecules (Supplemental Table S2). The Chl *a*/*c* ratio is 7.95, which is higher than that in diatom FCPI antennas (6.82) (Xu et al. 2020). The Chl *c* content per ACPI is 1.36, which is similar to that of FCPI (1.40) (Xu et al. 2020). The Chl/Car ratio is 2.39, which is comparable to that (2.44) for red algal LHCRs but greater than that (1.94) for diatom FCPIs (Pi et al. 2018; Xu et al. 2020); these results suggest that the capacity for blue-light absorption by ACPIs is likely lower than that of FCPIs.

**Figure 4.**
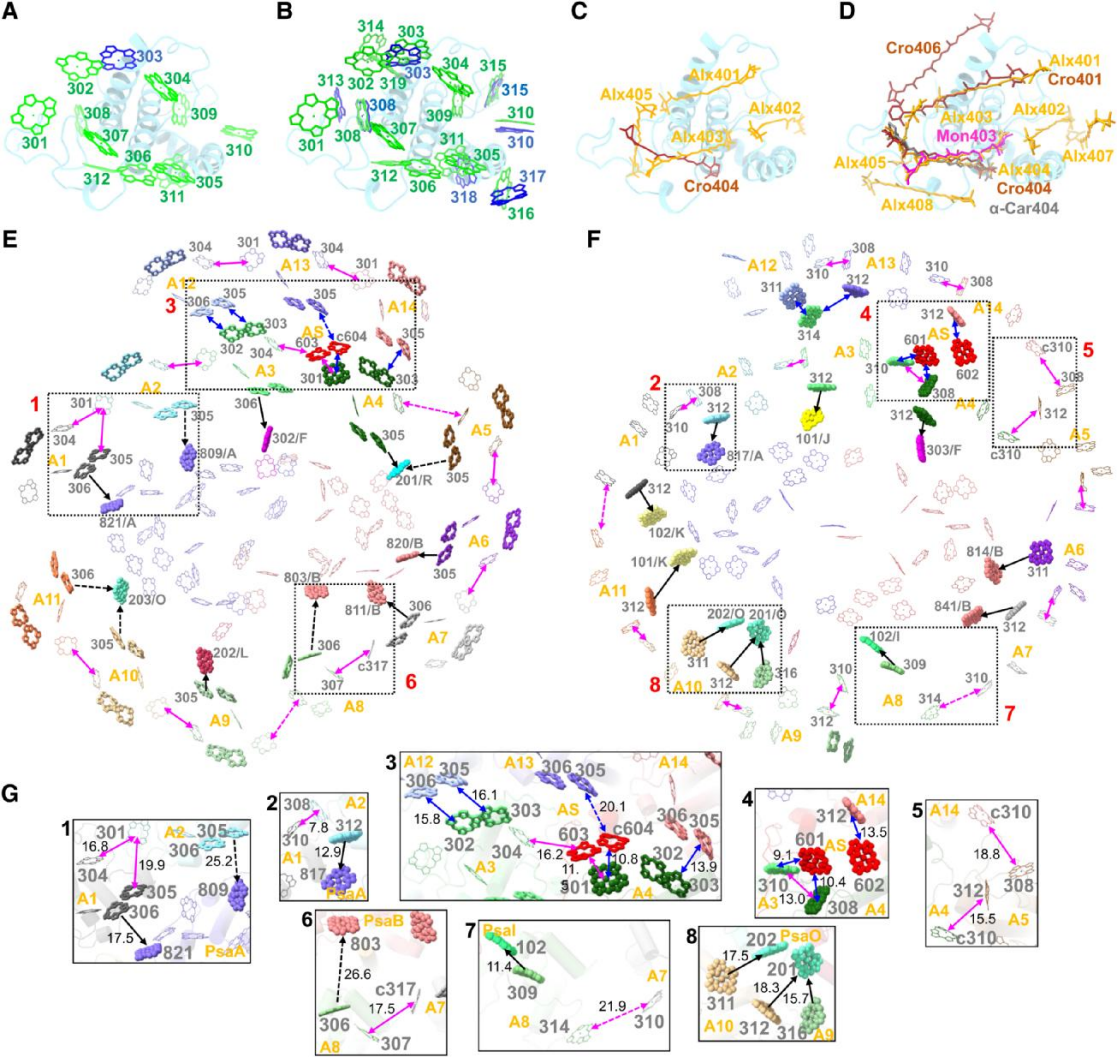
Arrangement of the pigments and possible energy transfer pathways in the PSI–ACPI supercomplex. **A)** Typical Chl sites in ACPI-2 viewed from the stromal side. Its 12 Chl sites are conserved in most ACPIs. Chls *a* and Chls *c* are colored green and blue, respectively. **B)** Nineteen Chl sites of all ACPIs viewed from the stromal side. The 303, 308, 310, and 315 sites can bind both Chl *a* (green) and Chl *c* (blue). **C)** Typical carotenoid sites in ACPI-2 viewed from the stromal side. Its 5 carotenoid sites are conserved in most ACPIs. **D)** Eight carotenoid-binding sites of all ACPIs viewed from the stromal side. Alloxanthin (Alx), crocoxanthin (Cro), monadoxanthin (Mon), and α-carotene (α-Car) are colored orange, brown, magenta, and gray, respectively. The 401, 403, and 404 sites can bind different types of carotenoids. Stromal-side view of the energy transfer pathways from the inner ACPIs to PSI core (black arrows), between the outer and inner layer ACPIs (blue double-headed arrows), and within the ACPI layers (magenta double-headed arrows) at the stromal **E)** and luminal **F)** layers. Zoom-in images of the boxed areas are shown in **(G)**. The Mg-to-Mg distances shorter or longer than 20 Å are indicated with solid or dotted arrows, respectively. A1 to A14 represent ACPI-1 to ACPI-14. AS represents ACPI-S. Chl pairs are highlighted as sticks. The interfacial Chls supporting energy transfer between the outer and inner ACPI layer and between the inner ACPIs and PSI core are shown as spheres. **G**) Zoom-in views of the squared areas in **(E)** and **(F)**. The Mg-to-Mg distances between Chls are labeled in Å.

Each Lhcr-type APCI apoprotein binds to 11 to 15 Chls, among which up to 3 are Chls *c* and the rest are Chls *a* (Supplemental Table S2). The Chl-binding sites 301 to 312 are conserved in many Lhcr-type ACPIs (Fig. 4A; Supplemental Fig. S22, A and B and Table S4; Movie 3), whereas ACPI-3/4/7/11/14 lack the 311 sites due to the absence of the histidine ligand (Supplemental Fig. S12). In contrast, other 7 Chl-binding sites (313 and 316 in ACPI-9, 314 and 318 in ACPI-3, 315 in ACPI-1/5/6/9/12, 317 in ACPI-7, and 319 in ACPI-12) are absent in red algal LHCRs, while Chls 314 to 317 of ACPIs share similar binding sites with Chls in diatom FCPIs (Supplemental Fig. S22, A and B). Chls 314/317 coordinated at the interface of adjacent ACPI subunits and Chls 316/318 at the interface of ACPIs and core subunits might mediate energy transfer between adjacent subunits (Supplemental Figs S23, A to D and S24A); Chl *c*313 binds to the C-terminal region of ACPI-9 and forms a Chl pair with Chl *c*308 (Supplemental Fig. S23E); Chl *a*315 binds to the special AE loop in most Group II ACPIs (Supplemental Fig. S23F); and Chl *a*319 binds to the C-terminal region of ACPI-12 (Supplemental Fig. S23G).

Unlike other ACPIs, ACPI-8 has only 8 Chl-binding sites (303 to 307, 309 to 310, 314), most of which are shifted due to deviations in protein structure, except for site 304 (Supplemental Fig. S18, A and B). A Chl-binding site 314 was identified in ACPI-8 that was not present in FCPI-1 (Supplemental Fig. S18E) (Xu et al. 2020). The pigments associated with ACPI-S are located at the interface between the ACPI inner and outer layers (Supplemental Fig. S23H), suggesting that ACPI-S functions in energy transfer between ACPIs.

Chl-binding sites 303, 308, 310, and 315 represent shared sites for either Chl *a* or Chl *c* (Fig. 4B; Supplemental Table S4). Two Chl pairs, 302/303 and 305/306, are present in all ACPIs, with an edge-to-edge distance of 3.6 to 3.9 Å, except for ACPI-8 (with only the Chl 305/306 pair) and ACPI-9 (containing an additional Chl pair 308/313) (Supplemental Figs S18B, 22C, S23E, and Table S4). The Chls *a*302/*a*303 homo-dimers are only present in ACPI-3 and ACPI-4 (Supplemental Fig. S24A) and might have lower energy than Chls *a*302/*c*303 due to the lower energy of Chl *a* than Chl *c* (Morosinotto et al. 2003; Qin et al. 2015; Su et al. 2019; Nagao et al. 2020), which may facilitate energy transfer from the outer ACPI-12/13/14 to ACPI-3/4.

Each APCI (except for ACPI-8) also contains 5 conserved carotenoid-binding sites (401 to 405) (Fig. 4C; Supplemental Table S4), as observed in red algal LHCRs and diatom FCPIs (Supplemental Fig. S22, D and E; Movie 3) (Pi et al. 2018; Antoshvili et al. 2019; Nagao et al. 2020; Xu et al. 2020). In contrast, ACPI-8 contains only 4 carotenoid-binding sites (401 to 403 and 407), similar to diatom FCPI-1 (Supplemental Fig. S18F). The 406 site exists only in ACPI-3 close to Chl *a*314, suggesting potential energy quenching (Fig. 4D; Supplemental Fig. 23A), whereas the 408 site exists only in ACPI-9. These 2 sites are absent in LHCRs and FCPIs (Supplemental Fig. S22, D and E).

### Energy transfer within the PSI–ACPI supercomplex

Our structural analysis allowed us to propose the possible excitation energy transfer (EET) pathways in cryptophyte PSI–ACPI based on the close Chl–Chl distances, as described in previous studies (Qin et al. 2015, 2019; Pan et al. 2018; Pi et al. 2018; Antoshvili et al. 2019; Su et al. 2019; Suga et al. 2019; Nagao et al. 2020; Xu et al. 2020; Yan et al. 2021). Chls in ACPIs can be divided into a stromal layer and a lumenal layer based on their spatial binding positions (Supplemental Fig. S24C). The Chl *a*305/*a*306 pairs are similar to the Chl pairs at the same locations in other LHCIs (Morosinotto et al. 2003; Qin et al. 2015, 2019; Mazor et al. 2017; Pi et al. 2018; Antoshvili et al. 2019; Su et al. 2019; Suga et al. 2019; Caspy et al. 2020; Nagao et al. 2020; Xu et al. 2020; Yan et al. 2021; Gorski et al. 2022). These are potential red-shifted Chls that might have lower energy in ACPIs. All Chl *a*305/*a*306 pairs are located at the interfaces between inner ACPIs and the PSI core as well as between outer and inner ACPIs at the stromal side (Fig. 4E), suggesting they might mediate EET from ACPIs to the PSI core. In addition, the Mg–Mg distances between Chl *a*305/*a*306 pairs and Chl *a*311/*a*312 in ACPI1–7/9 to 14 and Chl *a*309 in ACPI-8 are approximately 13 Å. This short distance may provide the structural basis for efficient EET between the stromal and lumenal Chl layers (Supplemental Fig. S24C).

Apart from vertical EET, EET in PSI–ACPI can be categorized into 3 possible lateral pathways: (i) between ACPIs within the same layer, (ii) from outer to inner ACPIs, and (iii) from inner ACPIs to the PSI core (Supplemental Fig. S24D). At the stromal side, EET within the same ACPI layer is primarily mediated by the Chl *a*305/*a*306 pairs, Chl *a*304 of 1 ACPI, and Chl *a*301 of the adjacent ACPI (Fig. 4, E and G; Supplemental Fig. S25A). At the lumenal side, Chl *a*/*c*310 is closely associated with Chl *a*308 in adjacent ACPI, facilitating EET within the same ACPI layer (Fig. 4, F and G; Supplemental Fig. S25A). Chls *a*603 and *a*601 of ACPI-S mediate stromal and lumenal EET between ACPI-3 and ACPI-4, respectively (Fig. 4, E to G; Supplemental Fig. S25B). In addition, EET between ACPI-4 and ACPI-5 is mediated by ACPI-4/Chl *c*310 and ACPI-5/Chl *a*312 at the lumenal side due to the larger distances between their Chls at the stromal side (Fig. 4, F and G). EET between ACPI-7 and ACPI-8 is mainly mediated by ACPI-7/Chl *c*317 and ACPI-8/Chl *a*307 at the stromal side, and EET between ACPI-8 and ACPI-9 is primarily mediated by ACPI-8/Chl *a*310 and ACPI-9/Chl *a*312 at the luminal side (Fig. 4, E to G). EET between ACPI-1 and ACPI-11 is likely less efficient due to the large gap between these structures (Fig. 4, E and F).

From the outer to inner ACPIs, EET at the stromal side is mediated by the Chl *a*305/*a*306 pair (Fig. 4E; Supplemental Table S5). The Chl *a*305/*a*306 pairs of ACPI-12 and ACPI-14 can transfer excitation energy directly to the Chl *a*302/*a*303 pairs of ACPI-3 and ACPI-4 in the inner layer, respectively, whereas the Chl *a*305/*a*306 pair of ACPI-13 can transfer energy to ACPI-4/Chl *a*301 via the Chl *a*603/*c*604 pair of ACPI-S (Fig. 4E; Supplemental Fig. S25, C to E). At the lumenal side, excitation energy can be transferred from ACPI-12/Chl *a*311 and ACPI-13/Chl *a*312 to ACPI-3/Chl *a*314 (Fig. 4F; Supplemental Table S5). ACPI-14/Chl *a*312 can transfer energy to ACPI-S/Chl *a*602 and then to ACPI-3/Chl *a*310 and ACPI-4/Chl *a*308 via ACPI-S/Chl *a*601 (Fig. 4F; Supplemental Fig. S25E). ACPI-14 can also transfer energy to ACPI-5 via ACPI-14/Chl *c*310 and ACPI-5/Chl *a*308 (Fig. 4, F and G). ACPI-S-mediated EET could be highly efficient due to the short distances between Chls of 9 to 14 Å.

From the inner ACPIs to the PSI core, Chl *a*305/*a*306 pairs mediate EET from ACPIs to the PSI core at the stromal side (Fig. 4E; Supplemental Tables S5 and S6). At the lumenal side, the EET pathways are mostly mediated via Chl *a*312 (Fig. 4F; Supplemental Tables S5 and S6). As ACPI-7 shifts outward from the PSI core, the EET between ACPI-7 and the PSI core is mediated by the Chl *a*841 in PsaB (Fig. 4F; Supplemental Fig. S6, Tables S5 and S6). EET between ACPI-10 and the PSI core is facilitated by the coupling of ACPI-10/Chl *a*311 and PsaO/Chl *a*202 (Fig. 4F; Supplemental Tables S5 and S6). The main EET pathways between ACPI-6/8/9 and the PSI core are through other Chls rather than Chl *a*312 (Fig. 4F; Supplemental Tables S5 and S6). EET from ACPI-2/8 to the PSI core mainly occurs at the lumenal side, given the larger Chl-Chl distances (≥25 nm) at the stromal side (Fig. 4, E to G). ACPI-5 has a larger distance from the PSI core than other ACPs, and its EET to the core is mediated by adjacent ACPIs (Fig. 4, E and F; Supplemental Table S5).

### The structure of cryptophyte PSI–ACPI provides insights into the evolution of red lineage PSI–LHCI

Our structural analysis revealed that cryptophyte PSI–ACPI shares common architectural features with red algal PSI–LHCR and diatom PSI–FCPI. Cryptophyte PSI–ACPI also possesses unique characteristics in terms of its protein organization, pigment association, and EET pathways. The cryptophyte PSI core contains PsaK and PsaO that are homologous to those of red algae but are absent in the diatom PSI core. PsaR is present in both the cryptophyte and diatom PSI cores but is absent in the red algal PSI core. PsaS was identified in the diatom PSI core but is absent in the cryptophyte and red algal PSI cores. The cryptophyte PSI core also contains the Unk1 subunit, which was not identified in the PSI cores of red algae, diatoms, or any other oxyphototrophs. Moreover, the number of antenna subunits of cryptophyte PSI–ACPI (14 ACPIs) lies between those of red algal PSI–LHCR (5 LHCRs) and diatom PSI–FCPI (24 FCPIs) (Pi et al. 2018; Nagao et al. 2020; Xu et al. 2020). Consistently, the variability of the number of PSI peripheral antennas has also been detected among evolutionarily distinct green-lineage photoautotrophs (Pan et al. 2020; Suga and Shen 2020; Bai et al. 2021). Most ACPIs share similar structures with red algal LHCRs, whereas ACPI-4 and ACPI-8 structurally resemble diatom FCPI-8 and FCPI-1, respectively. The arrangements of ACPI-1/2/3/7/8 are similar to those of their counterparts in red algal PSI–LHCR and diatom PSI–FCPI, and ACPI-4/5/6 share similar positions with their counterparts in diatom PSI–FCPI (due to the presence of PsaR). In contrast, the organizations of ACPI-9/10/11/12/13/14 are unique to cryptophyte PSI–ACPI, which are mediated by PsaK, PsaO, and ACPI-S (Supplemental Fig. S20; Movie 1).

Chl *c* was identified in ACPIs and FCPIs, but not in LHCRs. Twelve Chl-binding sites (301 to 312) are highly conserved in ACPIs, LHCRs, and FCPIs (Supplemental Fig. S22). In addition, ACPIs possess 7 Chl-binding sites not present in LHCRs, but lack 1 Chl *a*616 site; compared with FCPIs, ACPIs lack 8 Chl sites but have 3 unique Chl-binding sites (313/318/319; Supplemental Fig. S22) (Xu et al. 2020). Moreover, 5 carotenoid-binding sites are conserved in ACPIs, LHCRs, and FCPIs, whereas ACPIs possess 3 unique carotenoid-binding sites compared with LHCRs and lack 3 carotenoid-binding sites but have 2 sites not present in FCPIs (Pi et al. 2018; Antoshvili et al. 2019; Nagao et al. 2020; Xu et al. 2020) (Supplemental Fig. S22).

Cryptophyte ACPI-1/2/3/7 share similar EET pathways with their counterparts in red algal PSI–LHCR and diatom PSI–FCPI, and ACPI-6/8 share similar EET pathways with FCPI-10/1 of diatom PSI–FCPI (Pi et al. 2018; Nagao et al. 2020; Xu et al. 2020). ACPI-9/10/11 possess unique EET pathways to the PSI core mediated by PsaK and PsaO (Fig. 4, E to G). The ACPI-S-mediated EET from outer ACPIs to inner ACPIs is unique to cryptophyte PSI–ACPI (Fig. 4, E to G). Moreover, the EET pathways via Chl *a*312 of ACPI-1/4/7 to the PSI core are absent in diatom PSI–FCPI due to the lack of the corresponding Chls in FCPI-5/8/11 (Nagao et al. 2020; Xu et al. 2020). The EET pathways between ACPI-4 and the PSI core are distinct from those of PSI–FCPI, given the shift of FCPI-8, the absence of Chl *a*312, the binding of the Chl *a*413, and the shift of Chl *a*405 in FCPI-8 (Supplemental Fig. S25F) (Xu et al. 2020). The binding of Unk1 leads to the organizational shift of ACPI-7 outward from the PSI core, which may result in less efficient EET from ACPI-7 to the PSI core compared to red algae and diatoms (Supplemental Fig. S20; Movies 2 and 3). ACPI-9/Chl *a*316 is a specific Chl that mediates EET from ACPI-9 to PsaO, which is absent in FCPI-2 at the similar position in diatom PSI–FCPI (Fig. 4, F and G).

**Figure 5.**
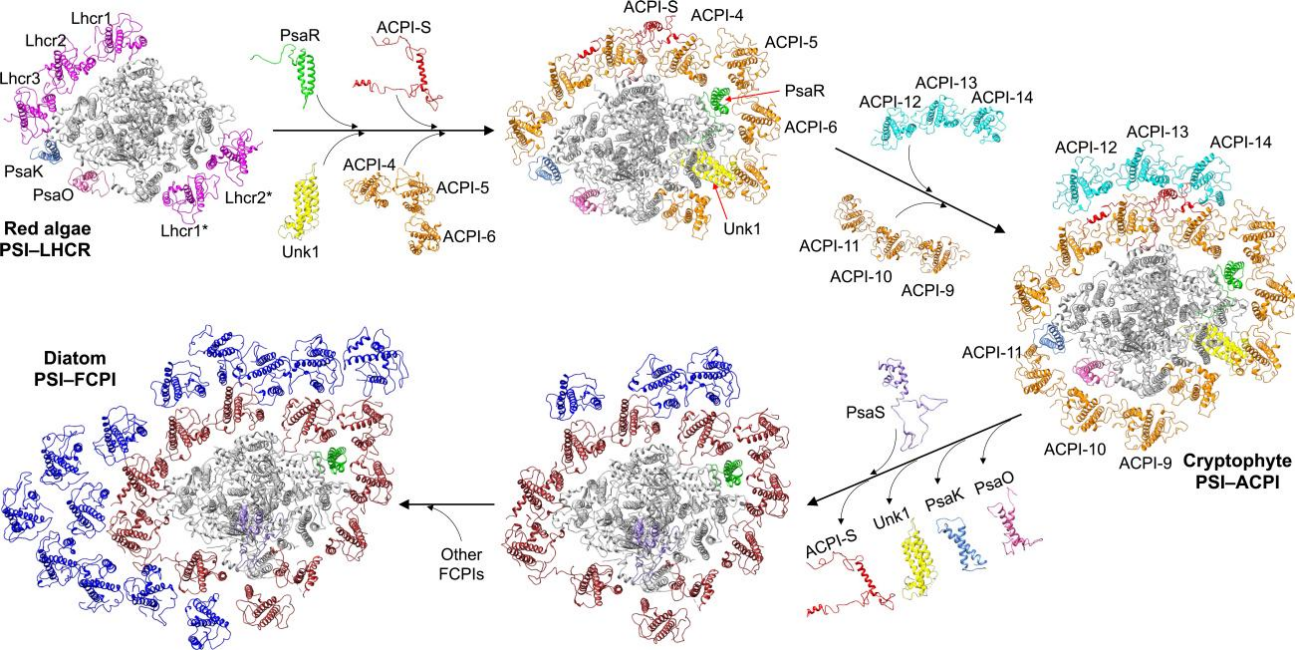
Possible evolutionary development of red-lineage PSI–LHCI supercomplexes. Cryptophyte PSI–ACPI represents an intermediate PSI–LHCI complex between the red algal PSI–LHCR and diatom PSI–FCPI complex. Briefly, during the evolution of the red-lineage PSI–LHCI supercomplex, PsaR subunit bound to the red algal PSI core and mediated the binding of ACPI-4/5/6 in cryptophyte PSI-ACPI, providing the structural basis for the association of ACPI-S, which mediated the association of outer ACPIs (ACPI-12/13/14) with inner ACPIs. Unk1 bound to cryptophyte PSI core at the lumenal side. ACPI-9/10/11 associated with the cryptophyte PSI core, forming an ACPI ring around the core. The PsaK, PsaO, Unk1, and ACPI-S subunits were lost during evolution from cryptophytes to diatoms, resulting in the conformational shifts of the corresponding FCPIs and facilitating the integration of other FCPIs to generate a large PSI–FCPI supercomplex.

The structural similarity and variations of red-lineage PSI–LHCI supercomplexes highlight the intermediate state of cryptophyte PSI–ACPI between red algal and diatom PSI–LHCI and provide important insights into the secondary endosymbiosis of red-lineage oxyphototrophs (Kooistra and Medlin 1996; Medlin et al. 1997; Shalchian-Tabrizi et al. 2008; Stiller et al. 2014; Zimorski et al. 2014) (Fig. 5). During the evolution of the red-lineage PSI–LHCI supercomplex, cryptophyte PsaR was integrated into the red algal PSI core and facilitated the binding of ACPI-4/5/6; Unk1 binds to PsaB and ACPI-7 at the lumenal side; ACPI-S binds to the inner ACPIs and mediates the association of outer ACPIs (ACPI-12/13/14) with inner ACPIs. ACPI-9/10/11 may then associate with the resulting cryptophyte PSI–ACPI intermediate structure. During secondary endosymbiosis from cryptophytes to diatoms, it is presumed that the loss of PsaK, PsaO, Unk1, and ACPI-S led to conformational shifts of the corresponding FCPIs and facilitated the integration of other FCPIs to generate a large PSI–FCPI supercomplex. The flexible loop structures of diatom FCPIs may reduce the stability of the PSI–FCPI supercomplex (Nagao et al. 2020; Xu et al. 2020), likely providing the structural basis for the photoacclimation of diatoms, allowing them to survive in specific ecological niches.

In summary, the cryo-EM structure of cryptophyte PSI–ACPI reveals the specific protein organization and pigment arrangement of the PSI core and associated antennas, providing insight into the fundamental mechanisms of light harvesting and energy transfer in PSI–ACPI. Our characterization of cryptophyte PSI–ACPI sheds light on the structural variations of PSI–LHCI in the red lineages and highlights the intermediate state of cryptophyte PSI–ACPI between red algal PSI–LHCR and diatom PSI–FCPI during secondary endosymbiosis.

## Materials and methods

### Purification of PSI–ACPI

*Chroomonas placoidea* T11 (a gift from Prof. Chen Min, College of Chemical and Biological Sciences and Engineering, Yantai University, Shandong, China) was cultured in F/2 medium under continuous light (40 *μ*mol photons m^−2^ s^−1^) (LED, T5, 23 W, 5,000 K) at 22 °C with continuous bubbling of air. Purification was performed at 4 °C under dim light. Cells in the logarithmic phase were pelleted by centrifugation at 6,000 × *g* for 10 min and washed with MES1 buffer (25 mM MES–NaOH, pH 6.5, 1.0 M betaine, 10 mM MgCl_2_) followed by another centrifugation at 6,000 × *g* for 10 min. The cell pellets were resuspended in MES1 and broken down with glass beads (diameter 212 to 300 *μ*m) (Casella et al. 2017; Zhao et al. 2020). Unbroken cells were removed by centrifugation at 3,000 × *g* for 10 min, and thylakoid membranes were collected by centrifugation at 21,000 × *g* for 30 min. The thylakoid membranes were washed with MES2 buffer (25 mM MES–NaOH, pH 6.5, 1.0 M betaine, 1 mM EDTA), resuspended in MES3 buffer (25 mM MES–NaOH, pH 6.5, 1.0 M betaine, 10 mM NaCl, 5 mM CaCl_2_) at 0.3 mg mL^−1^ Chl *a*, and solubilized with 0.9% (w/v) n-dodecyl-α-D-maltopyranoside (α-DDM) (Anatrace, USA) for 5 min on ice. The mixture was centrifuged for 30 min at 21,000 × *g*, and the supernatant was loaded onto a discontinuous sucrose gradient (10% to 30%) with an interval of 2% in MES3 buffer containing 0.02% α-DDM, followed by centrifugation at 230,000 × *g* for 20 h (Beckman SW41 rotor). The PSI–ACPI band was collected and further purified by size-exclusion chromatography (GE; Superose 6 Increase 10/300 GL) in MES4 buffer (25 mM MES–NaOH, pH 6.5, 0.5 M betaine, 50 mM NaCl, 5 mM CaCl_2_, 0.02% α-DDM). The peak fractions were collected and concentrated using an Amicon Ultra 100 kDa cutoff filter (Millipore) at 4,000 g.

### Characterization of PSI–ACPI

Absorption spectra were measured at room temperature using a UV–Vis Spectrophotometer (UV-1900, Shimadzu). The PSI–ACPI supercomplexes were denatured as described (Ma et al. 2017) and separated by 8% to 16% SDS-PAGE.

For mass spectrometry, Coomassie Brilliant blue-stained bands were cut out from the gel, reduced with dithiothreitol, alkylated with iodoacetamide, and digested using sequencing-grade modified trypsin, and the resulting peptides were extracted. The peptides were separated through a reverse phase trap column (nanoViper C18, 100 *μ*m × 2 cm, Thermo Fisher) connected to the C18-reversed phase analytical column (75 *μ*m × 10 cm, 3 *μ*m resin, Thermo Fisher) with an EASY-nLC 1000 System, which was coupled to a Q Exactive mass spectrometer (Thermo Fisher). The MS/MS spectra from each LC–MS/MS run were searched using MASCOT engine (version 2.4) against the selected database using the Proteome Discovery searching algorithm (version 1.4).

Pigment composition was analyzed by HPLC (Shimadzu, Japan) using a C18 reversed-phase column (Waters, Ireland) with a Shimadzu photodiode array detector. Pigment extraction and elution were performed as described previously (Xu et al. 2020). The elutes were detected at 445 nm with a wavelength detection range of 300 to 800 nm. As reported previously (Pennington et al. 1985; Schagerl and Donabaum 2003; Roy et al. 2011; Takaichi et al. 2016), the major pigments of cryptophytes are chlorophyll *c* (Chl *c*), alloxanthin (Alx), monadoxanthin (Mon), crocoxanthin (Cro), chlorophyll *a* (Chl *a*), and α-carotene (α-Car). Pigments were identified based on the characteristic absorption peaks of their absorption spectra and elution profiles (Wright and Jeffrey 2006; Roy et al. 2011). Three major pigments (Chl *a*, Chl *c*, and Alx) were purified by HPLC from pigment extracts of *C. placoidea* thylakoid membranes and used as standards to measure the pigment contents in the samples based on the absorption peak area of the HPLC profile.

### Sequence analysis of PSI–ACPI from *C. placoidea*

Total RNA was extracted from *C. placoidea* and subjected to transcriptome sequencing by BioMarker (BMK). Sequencing libraries were generated using a NEBNext Ultra RNA Library Prep Kit for Illumina (NEB, MA, USA) following the manufacturer's recommendations. The first-strand cDNA was synthesized using random hexamer primers and RNase H, and the second-strand cDNA was synthesized using DNA polymerase I and RNase H. Terminal repair, A-tailing, and adapter addition were performed to prepare the cDNA for hybridization. The cDNA fragments ∼240 bp in length were selected using the AMPure XP system (Beckman Coulter, Beverly, USA). The cDNA was treated with USER Enzyme (NEB), and PCR was performed to obtain the final cDNA library. After clustering of the index-coded samples, the library preparations were sequenced on an Illumina HiSeq 2000 platform, and paired-end reads were generated. The transcriptome was assembled de novo based on the left.fq and right.fq using Trinity (Grabherr et al. 2011). Sequences of the PSI core and ACP subunits were identified via comparison to sequences in the National Center for Biotechnology Information databases. The sequences of the PSI core subunits are completely identical to those in *C. placoidea* strain CCAP978/8.

### Phylogenetic analysis

Sequence alignments in Supplemental Figs S7 and S12 were performed with CLC Sequence Viewer 8.0 and ESPript 3.0 (Robert and Gouet 2014). Protein sequences used to produce phylogenetic tree in Supplemental Fig. S15 were imported in MEGA X and aligned with MUSCLE (default parameters; Supplemental File S1). The alignment was used to produce a phylogenetic tree with MEGA X (Kumar et al. 2018) (Supplemental File S2). The evolutionary history was inferred using the Neighbor-Joining method (Saitou and Nei 1987). The optimal tree with a sum of branch length = 13.99626746 is shown. The percentage of replicate trees in which the associated taxa clustered together in the bootstrap test (1,000 replicates) are shown next to the branches (Felsenstein 1985). The tree is drawn to scale, with branch lengths in the same units as those of the evolutionary distances used to infer the phylogenetic tree. The evolutionary distances were computed using the Poisson correction method (Zuckerkandl and Pauling 1965) and are expressed as the number of amino acid substitutions per site. This analysis involved 38 amino acid sequences. All positions with <95% site coverage were eliminated, i.e. fewer than 5% alignment gaps, missing data, and ambiguous bases were allowed at any position (partial deletion option). The final dataset contained 107 positions.

### Cryo-EM data collection

An aliquot of 4 *μ*L of PSI–ACPI sample at a Chl concentration of 2.0 mg mL^−1^ was applied to a freshly glow-discharged holey carbon grid (Quantifoil Au R2/1, 200 mesh) with continuous carbon support. The grid was blotted for 2 s at 100% humidity at 8 °C with a force level of 2 and immediately plunged into liquid ethane cooled by liquid nitrogen with Vitrobot Mark IV (Thermo Fisher, USA). The grids were loaded into a 300 kV Titan Krios G^3i^ microscope (Thermo Fisher) equipped with a K3 BioQuantum direct electron detector (Gatan, USA) for data acquisition. A total of 9,688 movie stacks were automatically recorded using EPU (Thermo Fisher) (Thompson et al. 2019) at a total dose for a stack of 50 *e*^−^ Å^−2^ in a defocus range of −1.0 to −1.8 *μ*m. A super-resolution mode was used at a nominal magnification of ×81,000 corresponding to a pixel size of 0.53 Å with the energy filter slit set to 20 eV.

### Data processing

All movie stacks were corrected by MotionCor2.1 (Zheng et al. 2017) with dose weighting. CTF parameters for each movie were estimated by CTFFIND-4 (Rohou and Grigorieff 2015). Image processing was mainly performed using cryoSPARC 3.1.1 (Punjani et al. 2017). After automatic particle picking and reference-free 2D classifications, 211,375 particles were selected, with obvious junk excluded from the particle set. The selected particles were used as templates for the template-picking procedure in cryoSPARC. The template-picked particles were processed by reference-free 2D classifications to remove bad particle images, and 582,601 particles were selected. After combining the 2 particle sets and removing duplicated particles, the remaining 412,630 particles were 3D classified into 5 classes, among which 2 classes with 118,810 and 133,521 particles, respectively, were subjected to homogeneous refinement. After 3D nonuniform refinement and sharpening, global (per-group) CTF refinement and local (per-particle) CTF refinement were performed. The overall resolutions of the maps of cryptophyte PSI–11ACPI and PSI–14ACPI were 2.71 and 2.66 Å, respectively. To improve the resolution of the cryo-EM density maps, particle subtraction was performed, followed by local refinement targeting the core complex and peripheral LHCIs (distinguished by LHCI-a, LHCI-b, and LHCI-c), resulting in final resolutions of 2.60, 2.98, 2.88, and 2.93 Å for PSI–11ACPI and 2.53, 2.76, 2.77, and 2.83 Å for PSI–14ACPI. The resolution was estimated based on the gold-standard Fourier shell correlation 0.143. The local resolution of the cryo-EM density map was generated using ResMap (Kucukelbir et al. 2014).

### Model building and refinement

For model building of the cryptophyte PSI–14ACPI supercomplex, the structure of red algal PSI–LHCR (PDB: 5ZGB) (Pi et al. 2018) was manually placed and rigid-body fitted into the 2.66 Å resolution cryo-EM map of PSI–14ACPI with UCSF Chimera (Pettersen et al. 2004). The amino acid sequences were then mutated to their counterparts in *C. placoidea* obtained from transcriptome sequencing, except for PsaM, whose sequence could not be found in the transcriptome sequence data and were modeled using sequences of *C. placoidea* CCAP978/8 (PsaM: YP_009420403.1), as other PSI core subunits of these 2 strains share the same sequences. PsaR was mutated from PsaR of *C. gracilis* PSI–FCPI (PDB: 6LY5) (Xu et al. 2020). The subunit Unk1 (chain X in PDB file) was constructed with polyalanines. Due to its low-resolution density map, a suitable sequence could not be identified from transcriptome sequences. All ACPIs were identified based on the best match of the amino acid sequence with the cryo-EM density map and were mutated from red algae LHCRs, except for ACPI-8, which was mutated from diatom FCPI-1 (Xu et al. 2020). De novo model building was performed on the ACPI-S subunit. Chl *c* was assigned as described in previous reports (Wang et al. 2019; Nagao et al. 2020). Chl *a* and Chl *c* were distinguished by the density map corresponding to the phytol chain for Chl *a* and the planarity of C-18^1^, C-18, C-17, and C-17^1^ resulting from the C-18=C-17 double bound for Chl *c*. Each residue and cofactor was manually checked and adjusted with COOT (Emsley et al. 2010). The geometrical restraints of pigments were generated from the Grade Web Server. To build the model of the PSI–11ACPI supercomplex, the PSI–14ACPI formation was initially fitted into the 2.71 Å resolution cryo-EM map, and the 3 additional ACPIs (9/10/11) were removed. The structures of the PSI–14ACPI and PSI–11ACPI supercomplexes were refined via real-space automatic refinement against the cryo-EM map by Phenix (Adams et al. 2010). Manual correction and automatic real-space refinement were carried out iteratively until the structure matched the cryo-EM density map to the maximum extent.

### Accession numbers

The cryo-EM map and atomic coordinates have been deposited in the Protein Data Bank and the Electron Microscopy Data Bank under accession numbers EMD-33659 and 7Y7B for the PSI–14ACPI supercomplex structure and EMD-33683 and 7Y8A for the PSI–11ACPI supercomplex structure.

## Supplementary Material

koad087_Supplementary_Data

## Supplemental data

The following materials are available in the online version of this article.

**Supplemental Figure S1**. Preparation and characterization of PSI–ACPI from *C. placoidea*.

**Supplemental Figure S2**. Cryo-EM data processing for the PSI–ACPI supercomplex.

**Supplemental Figure S3**. Evaluation of cryo-EM map quality.

**Supplemental Figure S4**. Comparison of the structures and pigment sites of 2 types of cryptophyte PSI–ACPI supercomplexes.

**Supplemental Figure S5**. Cryo-EM density maps and structures of the ACPI-S, Unk1 subunits, and pigment molecules.

**Supplemental Figure S6**. Comparison of the PSI cores of the cryptophyte *C. placoidea*, red alga *C. merolae* and diatom *C. gracilis*.

**Supplemental Figure S7**. Comparison of the sequences of PSI–ACPI from *C. placoidea* (*C.p.*) with the corresponding sequences from other algae.

**Supplemental Figure S8**. Location of the PsaR subunit and its interactions with surrounding subunits.

**Supplemental Figure S9**. Structures of the PsaK, PsaO, and PsaL subunits and their interactions with surrounding subunits.

**Supplemental Figure S10**. Comparison of PsaK and PsaO from cryptophytes, red algae (PDB: 5ZGB), green algae (PDB: 6SL5), moss (PDB: 7KSQ), and land plants (PDB: 5ZJI).

**Supplemental Figure S11**. Structures of individual ACPI subunits.

**Supplemental Figure S12**. Sequence alignment of ACPIs from *C. placoidea*.

**Supplemental Figure S13**. Pigment association and location of ACPI-S.

**Supplemental Figure S14**. Interactions between ACPI-S and surrounding subunits.

**Supplemental Figure S15**. Phylogenetic tree of LHCIs in the cryptophyte *C. placoidea* (*C.p*.) (green), red alga *C. merolae* (*C.m.*) (orange), and diatom *C. gracilis* (*C.g.*) (blue).

**Supplemental Figure S16**. Structural comparison of the ACPI structures and between ACPIs and Lhcrs from the red alga *C. merolae* and FCPIs from the diatom *C. gracilis*.

**Supplemental Figure S17**. Location of ACPI-4 viewed from the stromal side (**A**), interactions of the C-terminal loop of ACPI-7 with ACPI-6 (**B**), and interactions between ACPI-8 and PSI core subunits at the lumenal side (**C–E**).

**Supplemental Figure S18**. Comparisons of pigment arrangements in ACPI-8 with ACPI-2 and diatom FCPI-1.

**Supplemental Figure S19**. The structures of 3 adjacent ACPIs used as the building modules.

**Supplemental Figure S20**. Structural comparison of PSI–ACPI of the cryptophyte *C. placoidea* with that of the red alga *C. merolae* and the diatom *C. gracilis*.

**Supplemental Figure S21**. Interactions between ACPIs in the same layer (black circle), between outer ACPIs and inner ACPIs (red circle), and between inner ACPIs and PSI core (blue circle) at the stromal side (**A**) and lumenal side (**B**).

**Supplemental Figure S22**. Comparisons of pigment arrangements in ACPIs with those in red alga Lhcrs and diatom FCPIs.

**Supplemental Figure S23**. The locations of Chl *a*314 and Cro406 (**A**), Chl *a*316 (**B**), Chl *c*317 (**C**), Chl *c*318 (**D**), Chl *a*313 (**E**), Chl 315 (**F**), Chl *a*319 (**G**), and pigments (Chl *a*: green, Chl *c*: blue, α-Car: gray) of ACPI-S (**H**).

**Supplemental Figure S24**. Arrangement of the pigments in PSI–ACPI.

**Supplemental Figure S25**. EET between ACPIs and from ACPI to the PSI core.

**Supplemental Table S1**. Cryo-EM data collection, refinement, and validation statistics.

**Supplemental Table S2**. Pigments and cofactors in cryptophyte PSI–ACPI.

**Supplemental Table S3**. Comparison of the protein subunits in PSI–LHCI of cryptophytes, cyanobacteria, red algae, diatom, green algae, and land plants.

**Supplemental Table S4**. Pigment binding sites in ACPIs.

**Supplemental Table S5**. Possible EET pathways from ACPIs to the PSI core.

**Supplemental Table S6**. EET pathways from inner ACPIs to the PSI core.

**Supplemental File S1**. Sequence alignment file for the phylogenetic analysis in Supplemental Fig. S15.

**Supplemental File S2**. Tree file for the phylogenetic analysis in Supplemental Fig. S15.

## References

[koad087-B1] Abidizadegan M , Peltomaa E, Blomster J. The potential of cryptophyte algae in biomedical and pharmaceutical applications. Front Pharmacol. 2021:11:618836. 10.3389/fphar.2020.618836 PMC7884888 33603668

[koad087-B2] Adams PD , Afonine PV, Bunkoczi G, Chen VB, Davis IW, Echols N, Headd JJ, Hung LW, Kapral GJ, Grosse-Kunstleve RW, et al *PHENIX*: a comprehensive python-based system for macromolecular structure solution. Acta Crystallogr D Biol Crystallogr. 2010:66(2):213–221. 10.1107/S0907444909052925 20124702 PMC2815670

[koad087-B3] Antoshvili M , Caspy I, Hippler M, Nelson N. Structure and function of photosystem I in *Cyanidioschyzon merolae*. Photosynth Res. 2019:139(1–3):499–508. 10.1007/s11120-018-0501-4 29582227

[koad087-B4] Bai T , Guo L, Xu M, Tian L. Structural diversity of photosystem I and its light-harvesting system in eukaryotic algae and plants. Front Plant Sci. 2021:12:781035. 10.3389/fpls.2021.781035 34917114 PMC8669154

[koad087-B5] Casella S , Huang F, Mason D, Zhao GY, Johnson GN, Mullineaux CW, Liu LN. Dissecting the native architecture and dynamics of cyanobacterial photosynthetic machinery. Mol Plant. 2017:10(11):1434–1448. 10.1016/j.molp.2017.09.019 29017828 PMC5683893

[koad087-B6] Caspy I , Malavath T, Klaiman D, Fadeeva M, Shkolnisky Y, Nelson N. Structure and energy transfer pathways of the Dunaliella Salina photosystem I supercomplex. Biochim Biophys Acta Bioenerg. 2020:1861(10):148253. 10.1016/j.bbabio.2020.148253 32569661

[koad087-B7] Chen M , Li SH, Sun L. A novel phycocyanin–Chla/c_2_–protein complex isolated from chloroplasts of *Chroomonas placoidea*. Chin Chem Lett. 2007:18(11):1374–1378. 10.1016/j.cclet.2007.09.025

[koad087-B8] Dekker JP , Boekema EJ. Supramolecular organization of thylakoid membrane proteins in green plants. Biochim Biophys Acta. 2005:1706(1–2):12–39. 10.1016/j.bbabio.2004.09.009 15620363

[koad087-B9] Emsley P , Lohkamp B, Scott WG, Cowtan K. Features and development of *Coot*. Acta Crystallogr D Biol Crystallogr. 2010:66(4):486–501. 10.1107/S0907444910007493 20383002 PMC2852313

[koad087-B10] Felsenstein J . Confidence limits on phylogenies: an approach using the bootstrap. Evolution. 1985:39(4):783–791. 10.2307/2408678 28561359

[koad087-B11] Gorski C , Riddle R, Toporik H, Da Z, Dobson Z, Williams D, Mazor Y. The structure of the *Physcomitrium patens* photosystem I reveals a unique Lhca2 paralogue replacing Lhca4. Nat Plants. 2022:8(3):307–316. 10.1038/s41477-022-01099-w 35190662

[koad087-B12] Grabherr MG , Haas BJ, Yassour M, Levin JZ, Thompson DA, Amit I, Adiconis X, Fan L, Raychowdhury R, Zeng Q, et al Full-length transcriptome assembly from RNA-seq data without a reference genome. Nat Biotechnol. 2011:29(7):644–652. 10.1038/nbt.1883 21572440 PMC3571712

[koad087-B13] Greenwold MJ , Cunningham BR, Lachenmyer EM, Pullman JM, Richardson TL, Dudycha JL. Diversification of light capture ability was accompanied by the evolution of phycobiliproteins in cryptophyte algae. Proc Biol Sci. 2019:286(1902):20190655. 10.1098/rspb.2019.0655 PMC6532512 31088271

[koad087-B14] Janssen J , Rhiel E. Evidence of monomeric photosystem I complexes and phosphorylation of chlorophyll a/c-binding polypeptides in *Chroomonas* sp. strain LT (Cryptophyceae). Int Microbiol. 2008:11(3):171–178. 10.2436/IM.V11I3.9667 18843595

[koad087-B15] Jordan P , Fromme P, Witt HT, Klukas O, Saenger W, Krauss N. Three-dimensional structure of cyanobacterial photosystem I at 2.5 angstrom resolution. Nature. 2001:411(6840):909–917. 10.1038/35082000 11418848

[koad087-B16] Kato K , Nagao R, Ueno Y, Yokono M, Suzuki T, Jiang TY, Dohmae N, Akita F, Akimoto S, Miyazaki N, et al Structure of a tetrameric photosystem I from a glaucophyte alga *Cyanophora paradoxa*. Nat Commun. 2022:13(1):1679. 10.1038/s41467-022-29303-7 35354806 PMC8967866

[koad087-B17] Kereiche S , Kouril R, Oostergetel GT, Fusetti F, Boekema EJ, Doust AB, van der Weij-de Wit CD, Dekker JP. Association of chlorophyll a/c(2) complexes to photosystem I and photosystem II in the cryptophyte Rhodomonas CS24. Biochim Biophys Acta. 2008:1777(9):1122–1128. 10.1016/j.bbabio.2008.04.045 18513489

[koad087-B18] Kim JI , Moore CE, Archibald JM, Bhattacharya D, Yi G, Yoon HS, Shin W. Evolutionary dynamics of cryptophyte plastid genomes. Genome Biol Evol. 2017:9(7):1859–1872. 10.1093/gbe/evx123 28854597 PMC5534331

[koad087-B19] Kooistra WH , Medlin LK. Evolution of the diatoms (Bacillariophyta). IV. A reconstruction of their age from small subunit rRNA coding regions and the fossil record. Mol Phylogenet Evol. 1996:6(3):391–407. 10.1006/mpev.1996.0088 8975694

[koad087-B20] Kucukelbir A , Sigworth FJ, Tagare HD. Quantifying the local resolution of cryo-EM density maps. Nat Methods. 2014:11(1):63–65. 10.1038/nmeth.2727 24213166 PMC3903095

[koad087-B21] Kumar S , Stecher G, Li M, Knyaz C, Tamura K. MEGA X: molecular evolutionary genetics analysis across computing platforms. Mol Biol Evol. 2018:35(6):1547–1549. 10.1093/molbev/msy096 29722887 PMC5967553

[koad087-B22] Ma F , Zhang X, Zhu X, Li TP, Zhan J, Chen H, He CL, Wang Q. Dynamic changes of IsiA-containing complexes during long-term iron deficiency in *Synechocystis* sp. PCC 6803. Mol Plant. 2017:10(1):143–154. 10.1016/j.molp.2016.10.009 27777125

[koad087-B23] Mazor Y , Borovikova A, Caspy I, Nelson N. Structure of the plant photosystem I supercomplex at 2.6 A resolution. Nat Plants. 2017:3(3):17014. 10.1038/nplants.2017.14 28248295

[koad087-B24] Medlin LK , Kooistra WH, Sims P, Wellbrock U. Is the origin of the diatoms related to the end-permian mass extinction? Nova Hedwigia. 1997:65(1–4):1–11. 10.1127/nova.hedwigia/65/1997/1

[koad087-B25] Morosinotto T , Breton J, Bassi R, Croce R. The nature of a chlorophyll ligand in Lhca proteins determines the far red fluorescence emission typical of photosystem I. J Biol Chem. 2003:278(49):49223–49229. 10.1074/jbc.M309203200 14504274

[koad087-B26] Mullineaux CW , Liu LN. Membrane dynamics in phototrophic bacteria. Annu Rev Microbiol. 2020:74(1):633–654. 10.1146/annurev-micro-020518-120134 32689916

[koad087-B27] Nagao R , Kato K, Ifuku K, Suzuki T, Kumazawa M, Uchiyama I, Kashino Y, Dohmae N, Akimoto S, Shen JR, et al Structural basis for assembly and function of a diatom photosystem I-light-harvesting supercomplex. Nat Commun. 2020:11(1):2481. 10.1038/s41467-020-16324-3 32424145 PMC7235021

[koad087-B28] Naschberger A , Fadeeva M, Klaiman D, Borovikova-Sheinker A, Caspy I, Nelson N, Amunts A. Structure of plant photosystem I in a native assembly state. PREPRINT (Version 1), available at Research Square. 2022. 10.21203/rs.3.rs-2406494/v1

[koad087-B29] Naschberger A , Mosebach L, Tobiasson V, Kuhlgert S, Scholz M, Perez-Boerema A, Ho TTH, Vidal-Meireles A, Takahashi Y, Hippler M, et al Algal photosystem I dimer and high-resolution model of PSI-plastocyanin complex. Nat Plants. 2022:8(10):1191–1201. 10.1038/s41477-022-01253-4 36229605 PMC9579051

[koad087-B30] Nelson N , Junge W. Structure and energy transfer in photosystems of oxygenic photosynthesis. Annu Rev Biochem. 2015:84(1):659–683. 10.1146/annurev-biochem-092914-041942 25747397

[koad087-B31] Pan X , Cao P, Su X, Liu Z, Li M. Structural analysis and comparison of light-harvesting complexes I and II. Biochim Biophys Acta Bioenerg. 2020:1861(4):148038. 10.1016/j.bbabio.2019.06.010 31229568

[koad087-B32] Pan X , Ma J, Su X, Cao P, Chang W, Liu Z, Zhang X, Li M. Structure of the maize photosystem I supercomplex with light-harvesting complexes I and II. Science. 2018:360(6393):1109–1113. 10.1126/science.aat1156 29880686

[koad087-B33] Pennington FC , Haxo FT, Borch G, Liaaen-Jensen S. Carotenoids of cryptophyceae. Biochem Syst Ecol. 1985:13(3):215–219. 10.1016/0305-1978(85)90029-8

[koad087-B34] Pettersen EF , Goddard TD, Huang CC, Couch GS, Greenblatt DM, Meng EC, Ferrin TE. UCSF Chimera–a visualization system for exploratory research and analysis. J Comput Chem. 2004:25(13):1605–1612. 10.1002/jcc.20084 15264254

[koad087-B35] Pi X , Tian L, Dai HE, Qin X, Cheng L, Kuang T, Sui SF, Shen JR. Unique organization of photosystem I-light-harvesting supercomplex revealed by cryo-EM from a red alga. Proc Natl Acad Sci U S A. 2018:115(17):4423–4428. 10.1073/pnas.1722482115 29632169 PMC5924924

[koad087-B36] Punjani A , Rubinstein JL, Fleet DJ, Brubaker MA. cryoSPARC: algorithms for rapid unsupervised cryo-EM structure determination. Nat Methods. 2017:14(3):290–296. 10.1038/nmeth.4169 28165473

[koad087-B37] Qin X , Pi X, Wang W, Han G, Zhu L, Liu M, Cheng L, Shen JR, Kuang T, Sui SF. Structure of a green algal photosystem I in complex with a large number of light-harvesting complex I subunits. Nat Plants. 2019:5(3):263–272. 10.1038/s41477-019-0379-y 30850820

[koad087-B38] Qin X , Suga M, Kuang T, Shen JR. Structural basis for energy transfer pathways in the plant PSI-LHCI supercomplex. Science. 2015:348(6238):989–995. 10.1126/science.aab0214 26023133

[koad087-B39] Robert X , Gouet P. Deciphering key features in protein structures with the new ENDscript server. Nucleic Acids Res. 2014:42(W1):W320–W324. 10.1093/nar/gku316 24753421 PMC4086106

[koad087-B40] Rohou A , Grigorieff N. CTFFIND4: fast and accurate defocus estimation from electron micrographs. J Struct Biol. 2015:192(2):216–221. 10.1016/j.jsb.2015.08.008 26278980 PMC6760662

[koad087-B41] Roy S , Llewellyn CA, Egeland ES, Johnsen G. Phytoplankton pigments: characterization, chemotaxonomy and applications in oceanography. Cambridge: Cambridge University Press; 2011.

[koad087-B42] Saitou N , Nei M. The neighbor-joining method: a new method for reconstructing phylogenetic trees. Mol Biol Evol. 1987:4(4):406–425. 10.1093/oxfordjournals.molbev.a040454 3447015

[koad087-B43] Schagerl M , Donabaum K. Patterns of major photosynthetic pigments in freshwater algae. 1. Cyanoprokaryota, Rhodophyta and Cryptophyta. Ann Limnol Int J Lim. 2003:39(1):35–47. 10.1051/limn/2003003

[koad087-B44] Shalchian-Tabrizi K , Minge MA, Espelund M, Orr R, Ruden T, Jakobsen KS, Cavalier-Smith T. Multigene phylogeny of Choanozoa and the origin of animals. PLoS One. 2008:3(5):e2098. 10.1371/journal.pone.0002098 PMC2346548 18461162

[koad087-B45] Stiller JW , Schreiber J, Yue J, Guo H, Ding Q, Huang J. The evolution of photosynthesis in chromist algae through serial endosymbioses. Nat Commun. 2014:5(1):5764. 10.1038/ncomms6764 25493338 PMC4284659

[koad087-B46] Su X , Ma J, Pan X, Zhao X, Chang W, Liu Z, Zhang X, Li M. Antenna arrangement and energy transfer pathways of a green algal photosystem-I-LHCI supercomplex. Nat Plants. 2019:5(3):273–281. 10.1038/s41477-019-0380-5 30850819

[koad087-B47] Suga M , Ozawa SI, Yoshida-Motomura K, Akita F, Miyazaki N, Takahashi Y. Structure of the green algal photosystem I supercomplex with a decameric light-harvesting complex I. Nat Plants. 2019:5(6):626–636. 10.1038/s41477-019-0438-4 31182847

[koad087-B48] Suga M , Shen JR. Structural variations of photosystem I-antenna supercomplex in response to adaptations to different light environments. Curr Opin Struct Biol. 2020:63:10–17. 10.1016/j.sbi.2020.02.005 32294569

[koad087-B49] Takaichi S . Carotenoids in algae: distributions, biosyntheses and functions. Mar Drugs. 2011:9(6):1101–1118. 10.3390/md9061101 21747749 PMC3131562

[koad087-B50] Takaichi S , Yokoyama A, Mochimaru M, Uchida H, Murakami A. Carotenogenesis diversification in phylogenetic lineages of Rhodophyta. J Phycol. 2016:52(3):329–338. 10.1111/jpy.12411 27273528

[koad087-B51] Thompson RF , Iadanza MG, Hesketh EL, Rawson S, Ranson NA. Collection, pre-processing and on-the-fly analysis of data for high-resolution, single-particle cryo-electron microscopy. Nat Protoc. 2019:14(1):100–118. 10.1038/s41596-018-0084-8 30487656 PMC7618007

[koad087-B52] Wang W , Yu LJ, Xu C, Tomizaki T, Zhao S, Umena Y, Chen X, Qin X, Xin Y, Suga M, et al Structural basis for blue-green light harvesting and energy dissipation in diatoms. Science. 2019:363(6427):eaav0365. 10.1126/science.aav0365 30733387

[koad087-B53] Wright SW , Jeffrey SW. Pigment markers for phytoplankton production. In: Volkman JK, editor. Marine organic matter: biomarkers, isotopes and DNA. Berlin and Heidelberg: Springer Science & Business Media; 2006. p. 71–104.

[koad087-B54] Xu C , Pi X, Huang Y, Han G, Chen X, Qin X, Huang G, Zhao S, Yang Y, Kuang T, et al Structural basis for energy transfer in a huge diatom PSI-FCPI supercomplex. Nat Commun. 2020:11(1):5081. doi 10.1038/s41421-021-00242-9 33033236 PMC7545214

[koad087-B55] Yan Q , Zhao L, Wang W, Pi X, Han G, Wang J, Cheng L, He YK, Kuang T, Qin X, et al Antenna arrangement and energy-transfer pathways of PSI-LHCI from the moss *Physcomitrella patens*. Cell Discov. 2021:7(1):10. 10.1038/s41421-021-00242-9 33589616 PMC7884438

[koad087-B56] Zhao LS , Huokko T, Wilson S, Simpson DM, Wang Q, Ruban AV, Mullineaux CW, Zhang YZ, Liu LN. Structural variability, coordination and adaptation of a native photosynthetic machinery. Nat Plants. 2020:6(7):869–882. 10.1038/s41477-020-0694-3 32665651

[koad087-B57] Zheng SQ , Palovcak E, Armache JP, Verba KA, Cheng Y, Agard DA. Motioncor2: anisotropic correction of beam-induced motion for improved cryo-electron microscopy. Nat Methods. 2017:14(4):331–332. 10.1038/nmeth.4193 28250466 PMC5494038

[koad087-B58] Zimorski V , Ku C, Martin WF, Gould SB. Endosymbiotic theory for organelle origins. Curr Opin Microbiol. 2014:22:38–48. 10.1016/j.mib.2014.09.008 25306530

[koad087-B59] Zuckerkandl E , Pauling L. Evolutionary divergence and convergence in proteins. In: Bryson V, Vogel HJ, editors. Evolving genes and proteins. New York and London: Academic Press; 1965. p. 97–166.

