# Supplementary material for: Structural basis and evolution of the photosystem I–light-harvesting supercomplex of cryptophyte algae
Source: Plant Cell. 2023 Mar 21;35(7):2449–63. doi: 10.1093/plcell/koad087 (PMC10291030; doi:10.1093/plcell/koad087)
Supplement: koad087_Supplementary_Data [file koad087_supplementary_data.zip › TPC2022RA00833R2_Supplemental_Figures_and_Tables.pdf]

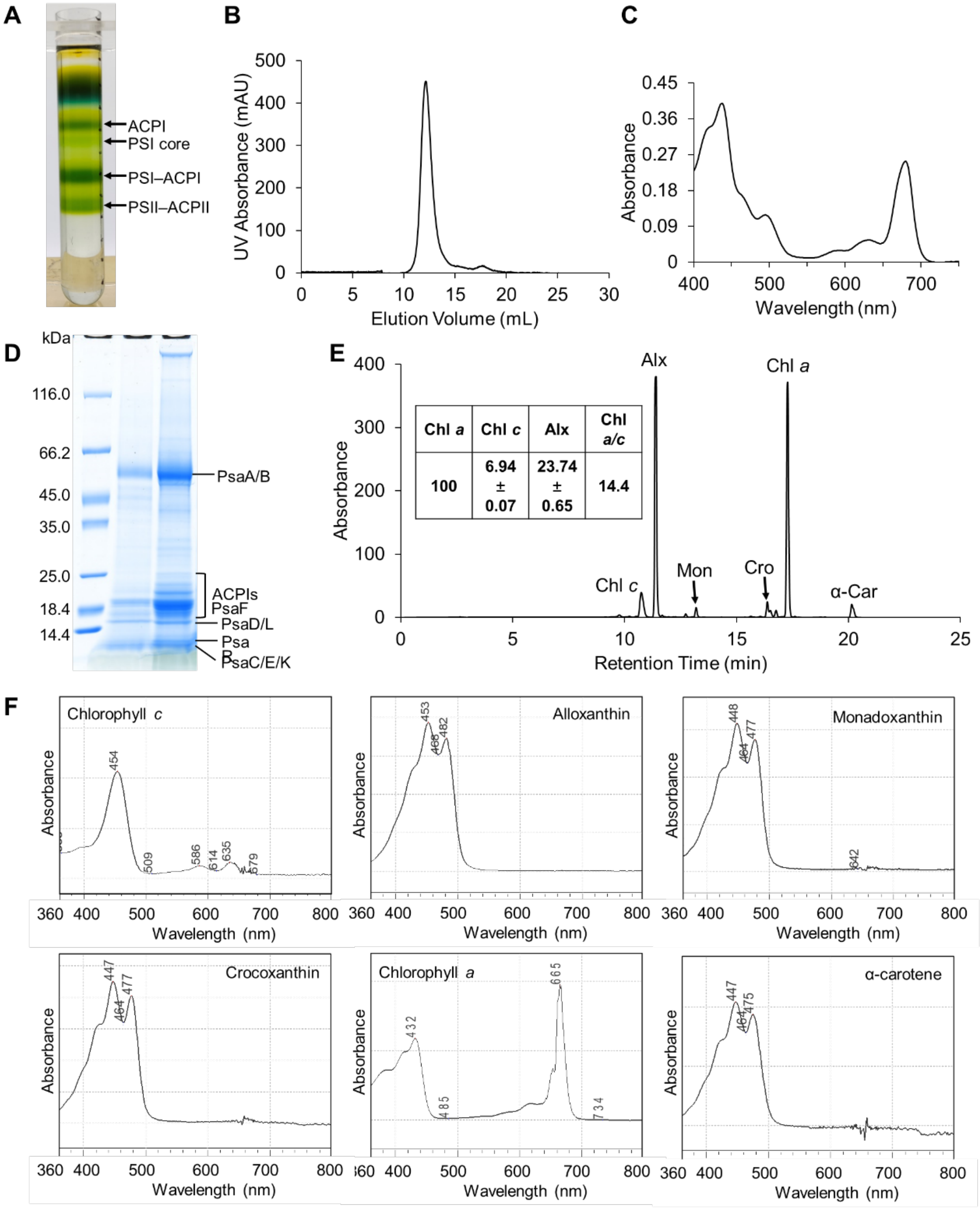

Chl *a*	Chl *c*	Alx	Chl *a/c*
100	6.94 ± 0.07	23.74 ± 0.65	14.4

Supplemental Figure S1. Preparation and characterization of PSI–ACPI from *C. placoidea* (Supports Figures 1 and 4). A, Purification of the PSI–ACPI supercomplex by sucrose density gradient centrifugation. **B**, Elution profile of the PSI–ACPI supercomplex from size-exclusion chromatography. **C**, Room-temperature absorption spectra of the PSI–ACPI supercomplex. **D**, SDS-PAGE analysis of the PSI–ACPI supercomplex. The protein composition of the bands is indicated based on tmass spectrometry analysis. **E**, Analysis of the pigment composition of PSI–ACPI by HPLC, recorded at 445 nm. Six major pigment peaks were identified based on the characteristic absorption spectrum of each peak fraction: chlorophyll *c* (Chl *c*), alloxanthin (Alx), monadoxanthin (Mon), crocoxanthin (Cro), chlorophyll *a* (Chl *a*) and α-carotene (α-Car). The relative contents of Chl *a*, Chl *c* and Alx are shown in the table, expressed as the number of pigments per 100 Chl *a*. **F**, Absorption spectra of the six major pigment peaks in **E**. These experiments were performed more than five times, and the same results were obtained reproducibly.

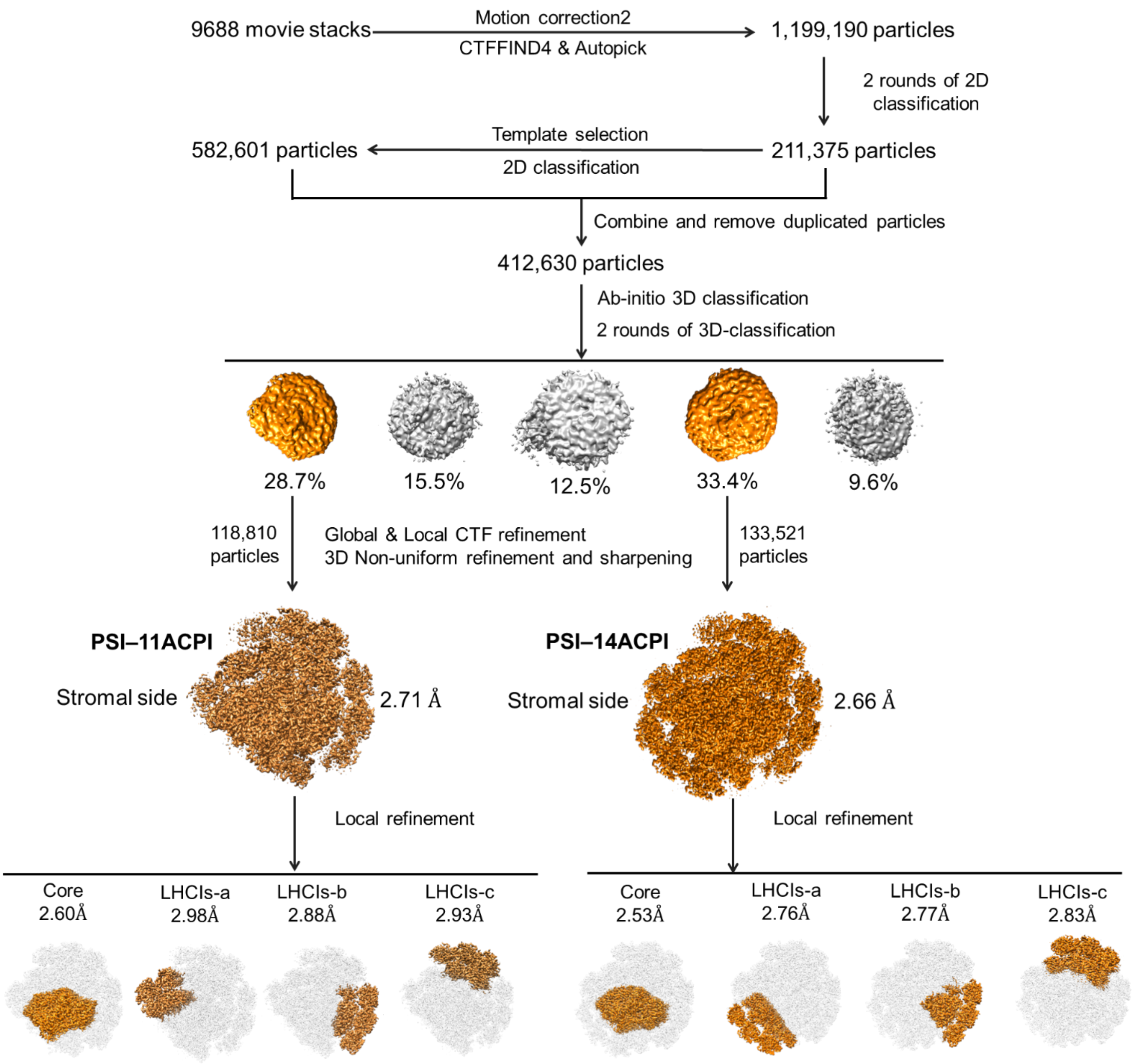

Supplemental Figure S2. Cryo-EM data processing for the PSI–ACPI supercomplex (Supports Figure 1).

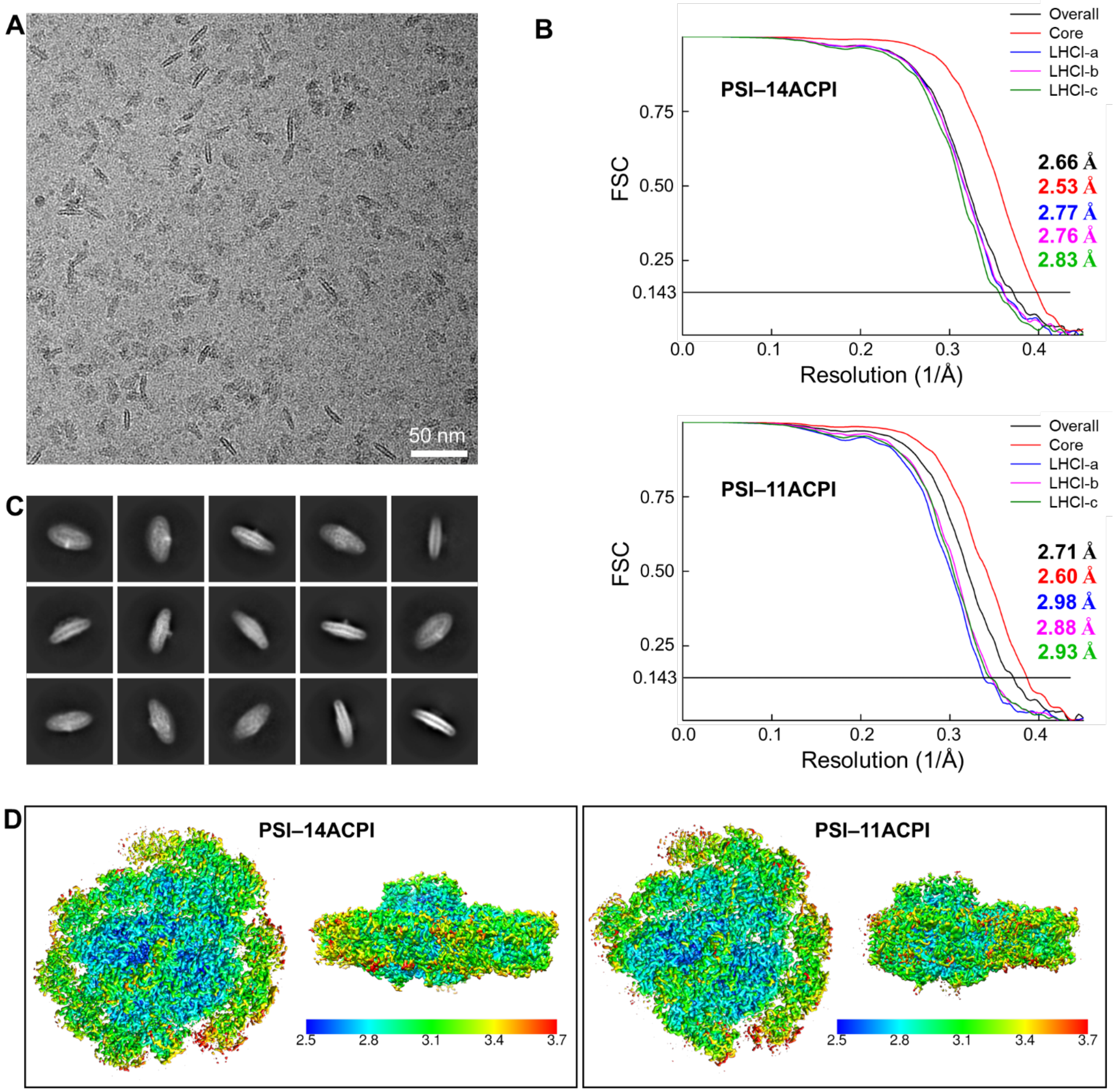

Supplemental Figure S3. Evaluation of cryo-EM map quality (Supports Figure 1). A, A representative cryo-EM micrograph of the cryptophyte PSI–ACPI supercomplex. **B**, Gold-standard Fourier shell correlation (FSC) curves of the final density map with the criterion of 0.143. **C**, Representative reference-free 2D class averages with 424 Å box size. **D**, Local resolution distributions of the cryo-EM map estimated by ResMap.

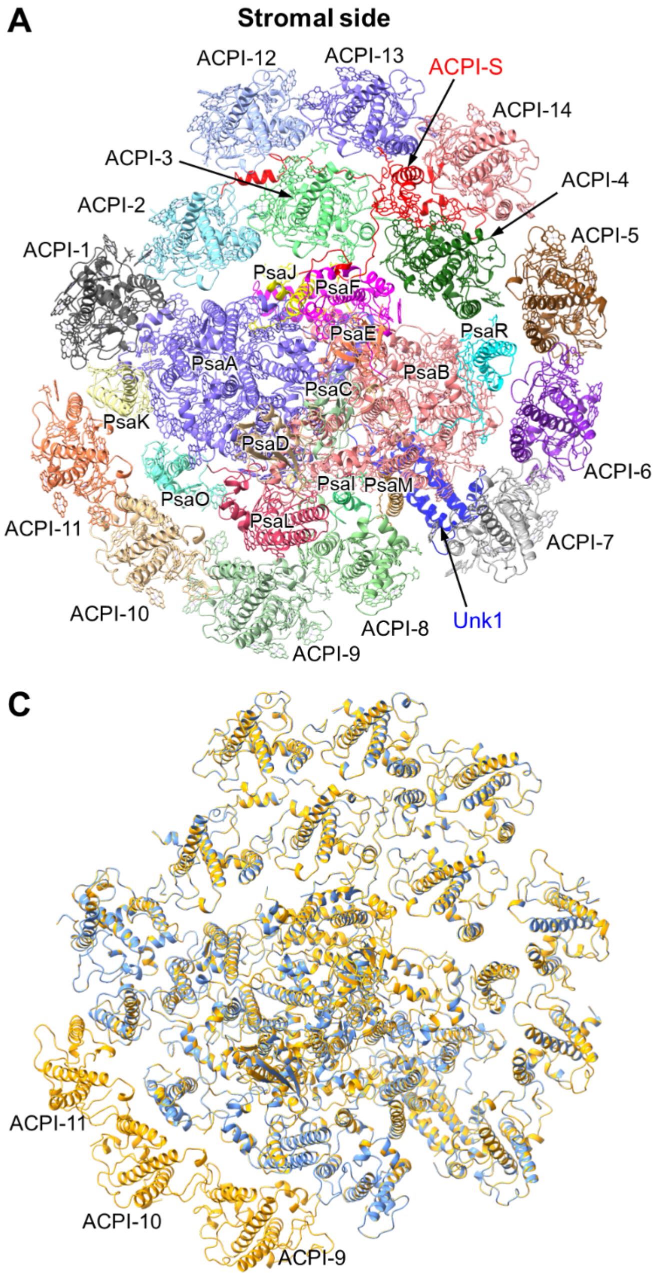

Supplemental Figure S4. Comparison of the structures and pigment sites of two types of cryptophyte PSI–ACPI supercomplexes (Supports Figure 1). A, PSI–ACPI with ACPI-9/10/11 viewed from the stromal side. The names of core subunits are labeled. LHCs are labeled as ACPI. The previously unidentified PSI core subunit and the subunit lying between ACPs are labeled as “Unk1” and “ACPI-S”, respectively. **B**, PSI–ACPI without ACPI-9/10/11 viewed from the stromal side. **C**, Superposition of the two types of PSI–ACPI. Orange: with ACPI-9/10/11; Blue: without ACPI-9/10/11. **D**, Superposition of the pigment sites of the two types of PSI–ACPI. Orange: with ACPI-9/10/11; Blue: without ACPI-9/10/11.

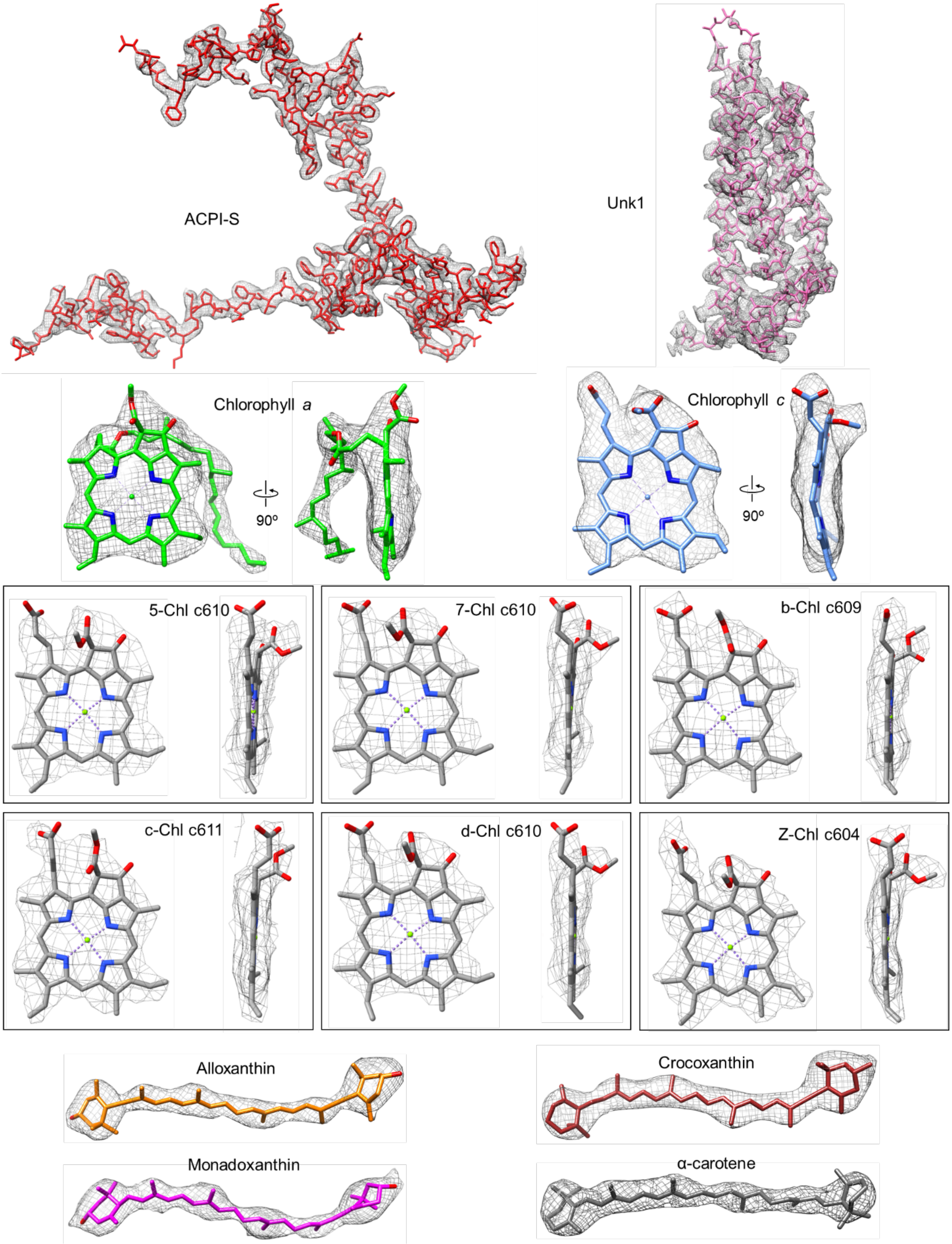

Supplemental Figure S5. Cryo-EM density maps and structures of the ACPI-S, Unk1 subunits, and pigment molecules (Supports Figures 1-4). Examples of typical maps of Chl *c* from the model are shown.

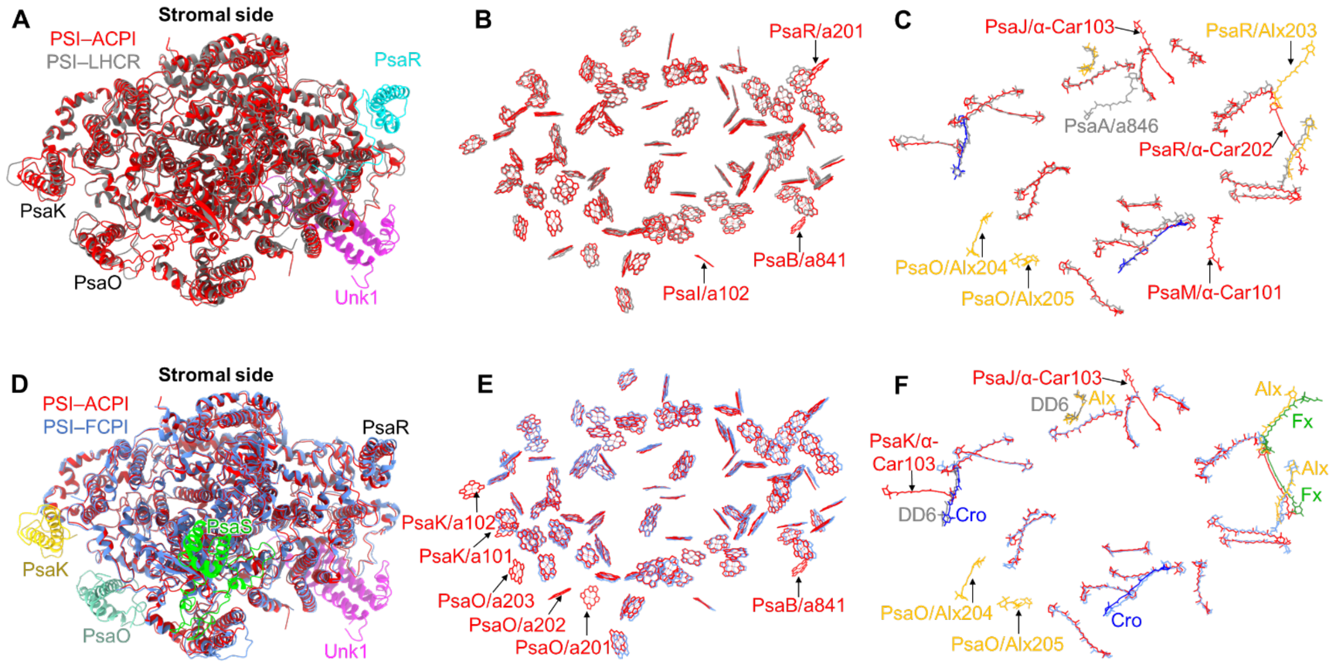

Supplemental Figure S6. Comparison of the PSI cores of the cryptophyte *C. placoidea*, red alga *C. merolae* and diatom *C. gracilis* (Supports Figures 1 and 4). A, Superposition of the cryptophyte PSI core (red) with red alga PSI core (gray). PsaR and newly identified Unk1, which are absent in the red algal PSI core, are indicated. **B**, Superposition of the Chl sites in the cryptophyte PSI core (red) with that in the red alga PSI core (gray). New Chl sites in the cryptophyte PSI core are labeled. **C**, Superposition of the carotenoid sites in the cryptophyte PSI core (red) with that in the red alga PSI core (gray). Alx and Cro in the cryptophyte PSI core are colored in orange and blue, respectively. Six new sites and one missing site in the cryptophyte PSI core are indicated. **D**, Superposition of the cryptophyte PSI core (red) with the diatom PSI core (cornflower blue). PsaK, PsaO and the newly identified subunit Unk1, which are absent in the diatom PSI core, are indicated. **E**, Superposition of the Chl sites in the cryptophyte PSI core (red) with that in the diatom PSI core (cornflower blue). New Chl sites in the cryptophyte PSI core is labeled. **F**, Superposition of the carotenoid sites in the cryptophyte PSI core (red) with that in the diatom PSI core (cornflower blue). Alx/Cro in the cryptophyte PSI core and Fx/DD6 in the diatom PSI core are colored in orange, blue, green and gray, respectively. Four new sites in the cryptophyte PSI core are indicated. All panels are viewed from the stromal side.

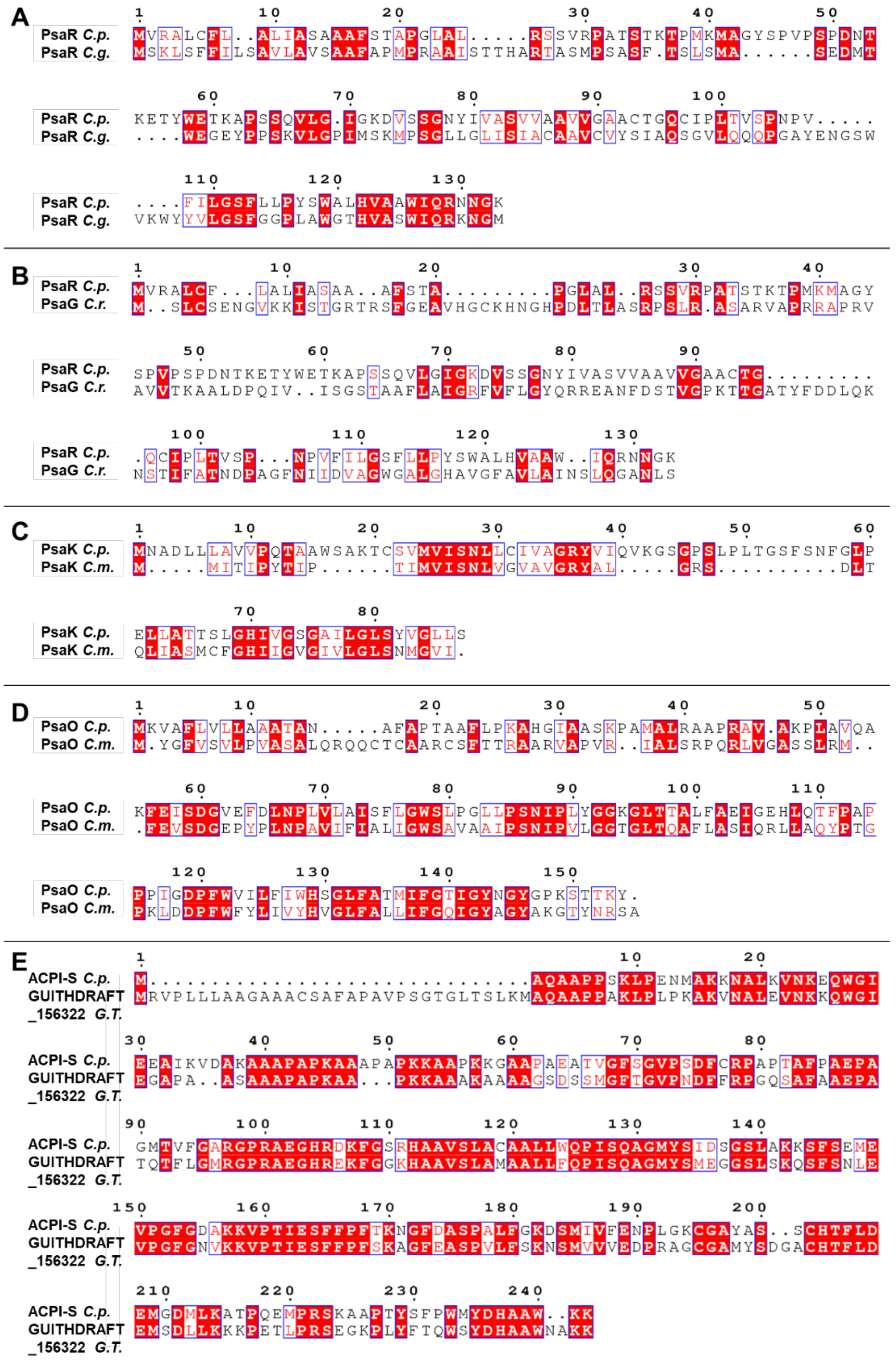

Supplemental Figure S7. Comparison of the sequences of PSI–ACPI from *C. placoidea* (*C.p.*) with the corresponding sequences from other algae (Supports Figures 2 and 3). A, Comparison of the sequences of PsaR from *C. placoidea* and *C. gracilis* (*C.g.*). **B**, Comparison of the sequences of PsaR from *C. placoidea* and PsaG from *Chlamydomonas reinhardtii* (*C.r.*). **C**, Comparison of the sequences of PsaK from *C. placoidea* and *C. merolae* (*C.m.*). **D**, Comparison of the sequences of PsaO from *C. placoidea* and *C. merolae*. **E**, Comparison of the sequence of ACPI-S from *C. placoidea* with the corresponding sequence GUITHDRAFT_156322 from *Guillardia theta* (*G.t.*). Fully conserved residues are shaded in red, and similar amino acids are highlighted by blue frames.

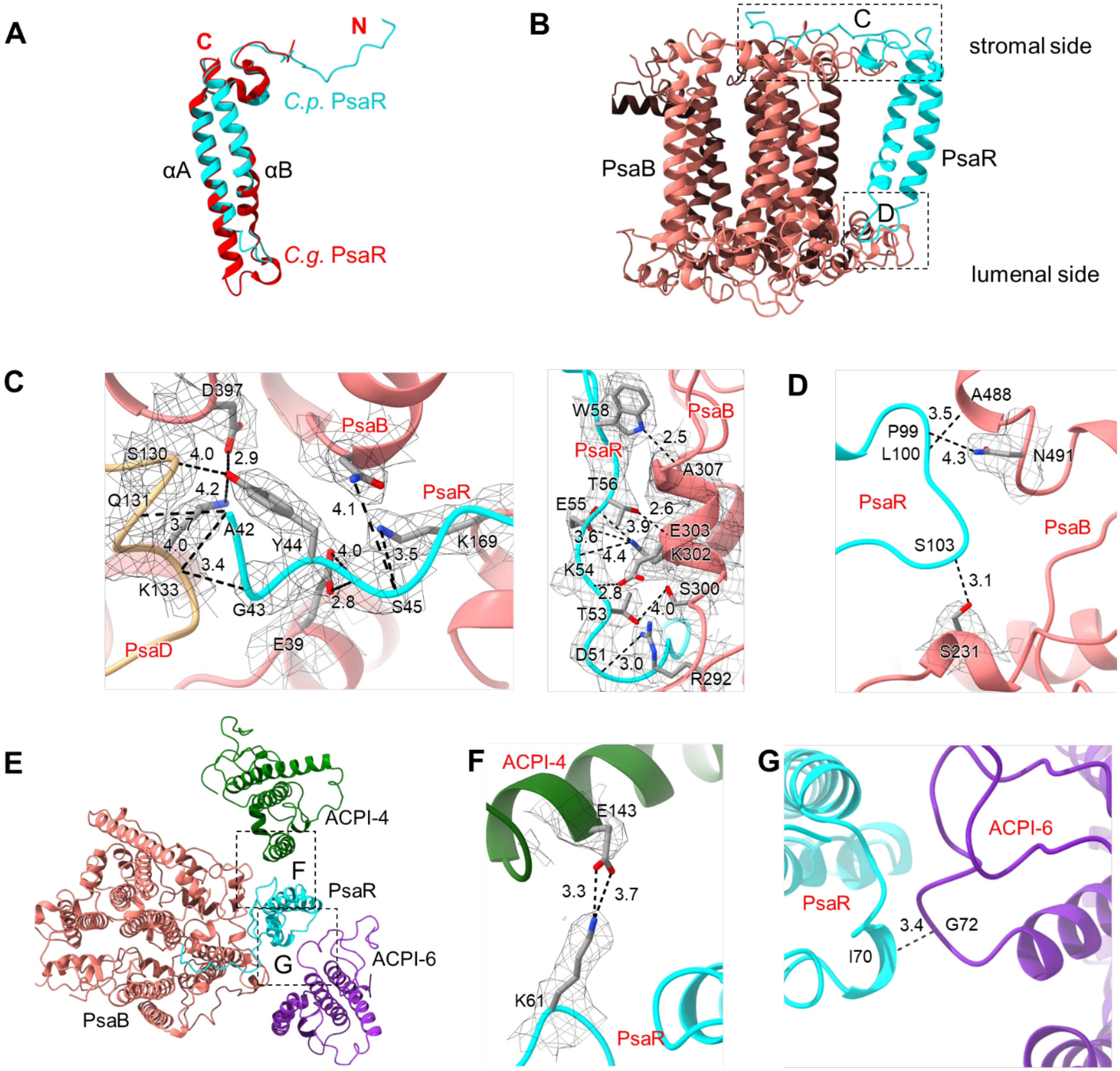

Supplemental Figure S8. Location of the PsaR subunit and its interactions with surrounding subunits (Supports Figure 2). A, Structural comparison between PsaR of *C. placoidea* (*C.p.*) (cyan) and PsaR of the diatom *C. gracilis* (*C.g.*) (red). **B**, Side view of PsaR-PsaB interactions. Squared areas are enlarged in **C** and **D**. **C**, Interactions between PsaR and PsaB at the stromal side. **D**, Interactions between PsaR and PsaB at the lumenal side. **E**, Interactions between PsaR and ACPIs viewed from the stromal side. Squared areas are enlarged in **F** and **G**. **F**, Interactions between PsaR and ACPI-4. **G**, Interactions between PsaR and ACPI-6. Interactions are indicated by black dashed lines with distances labeled in Å. The density maps of related residues are shown.

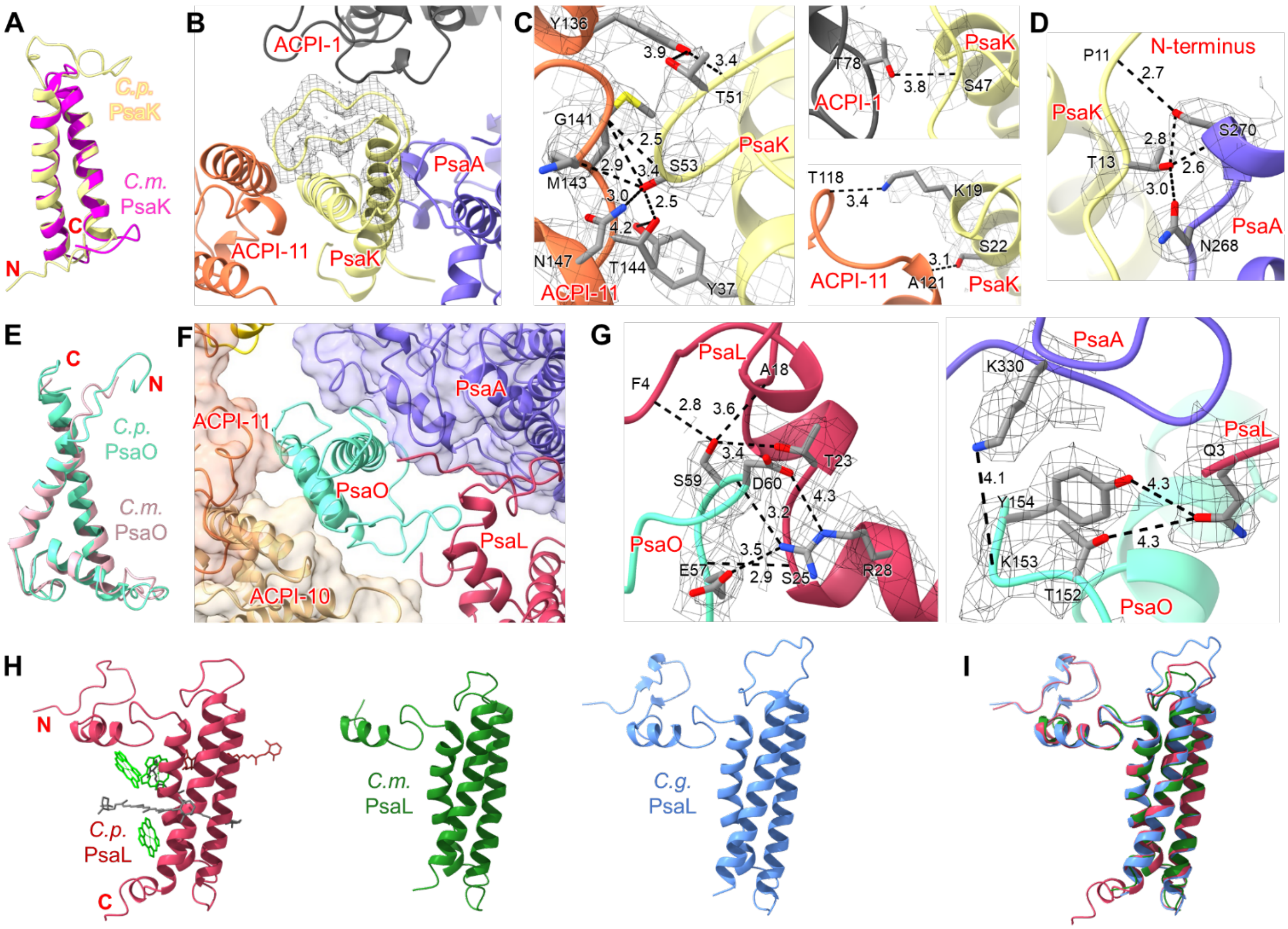

Supplemental Figure S9. Structures of the PsaK, PsaO and PsaL subunits and their interactions with surrounding subunits (Supports Figure 2). A, Structural comparison between PsaK of *C. placoidea* (*C.p.*) and PsaK of *C. merolae* (*C.m.*). **B**, The location of PsaK in PSI–ACPI viewed from the stromal side. A map of the stromal loop of PsaK is shown. **C**, Interactions between PsaK and surrounding ACPI subunits. **D**, Interactions between PsaK and PsaA subunits. **E**, Structural comparison between PsaO of *C. placoidea* and PsaO of *C. merolae*. **F**, The location of PsaO in PSI–ACPI viewed from the stromal side. **G**, Interactions between PsaO and surrounding subunits. **H**, Structural comparison of PsaL from the cryptophyte *C. placoidea* (left), red alga *C. merolae* (middle), and diatom *C. gracilis* (right). **I**, Superposition of three PsaL subunits in **H**. Interactions are indicated by black dashed lines with distances labeled in Å. The density maps of related residues are shown.

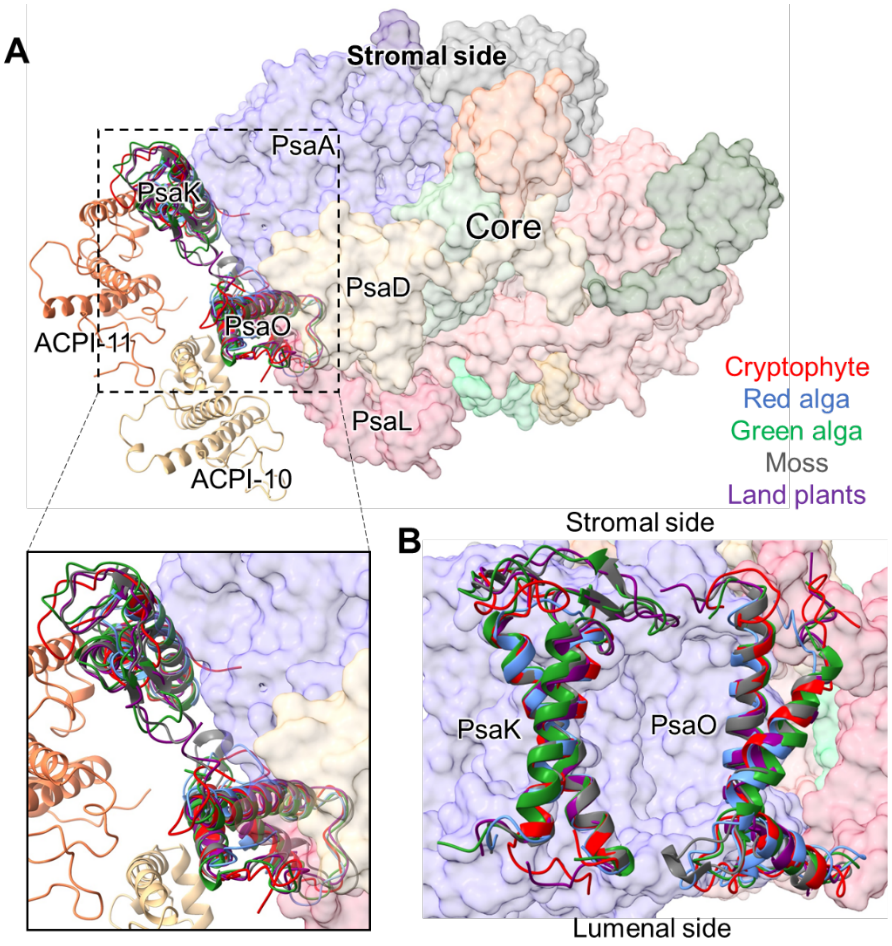

Supplemental Figure S10. Comparison of PsaK and PsaO from cryptophytes, red algae (PDB: 5ZGB), green algae (PDB: 6SL5), moss (PDB: 7KSQ), and land plants (PDB: 5ZJI) (Supports Figure 2). A, Stromal side view of PsaK and PsaO. **B**, Side view of PsaK and PsaO in panel **A**.

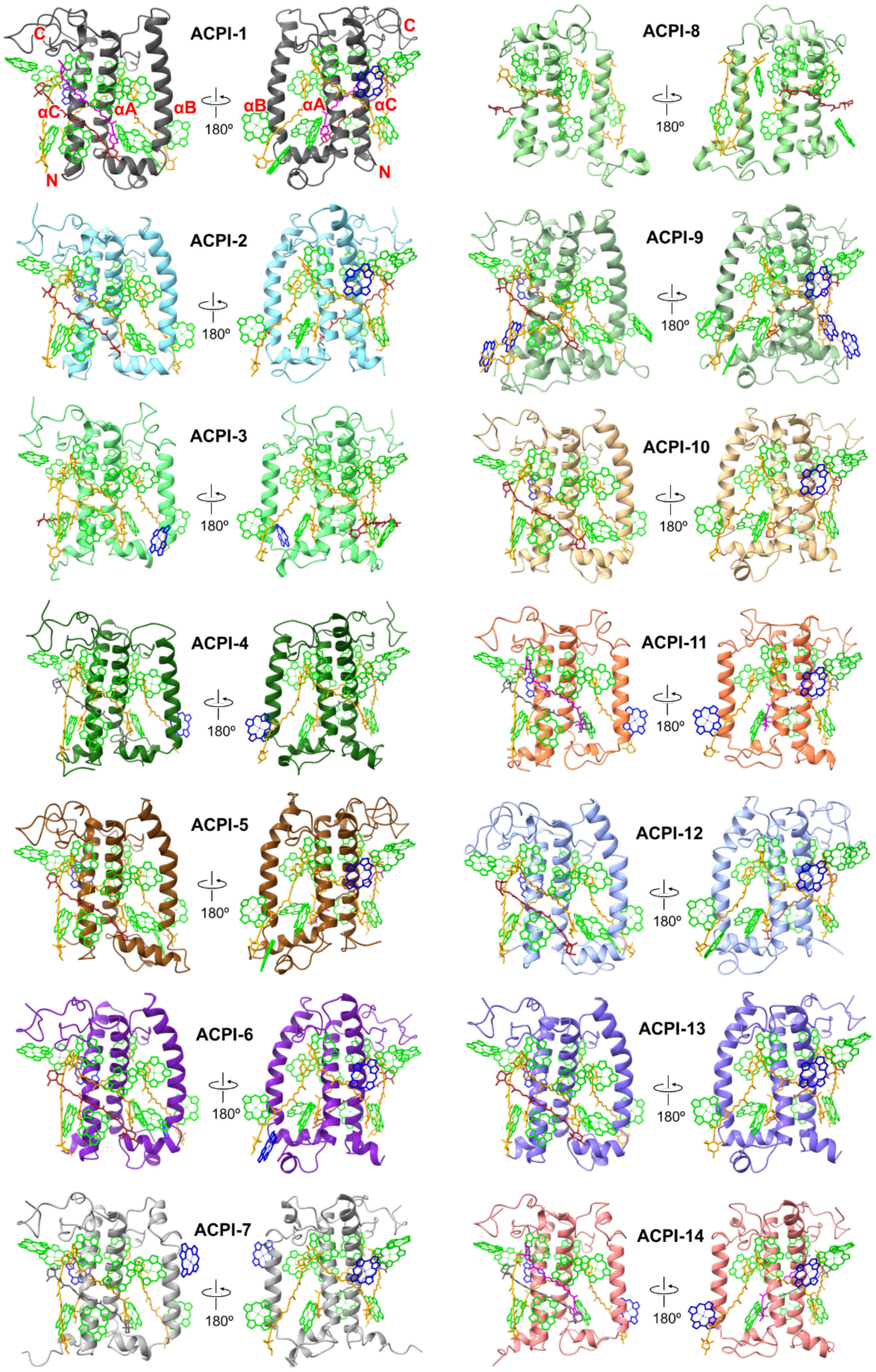

Supplemental Figure S11. Structures of individual ACPI subunits (Supports Figure 3). Chl *a*, Chl *c*, alloxanthin, crocoxanthin, monadoxanthin, and α-carotene are colored green, blue, orange, brown, magenta, and gray, respectively, with the phytol chains omitted. The helices and termini of ACPI-1 are labelled; the same labeling strategy applies to the other panels.

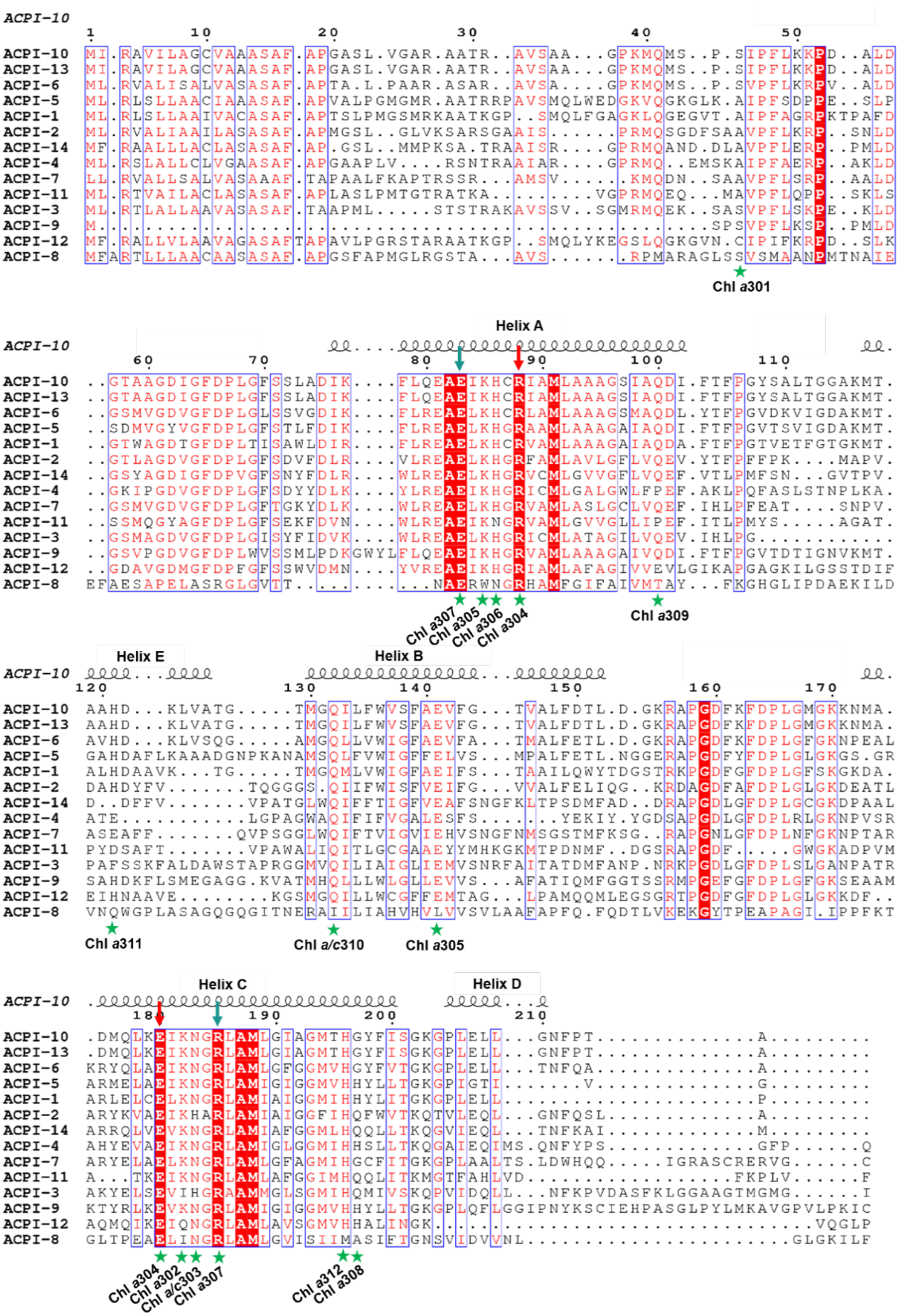

Supplemental Figure S12. Sequence alignment of ACPIs from *C. placoidea* (Supports Figure 3). The secondary structure is shown above the sequences. Fully conserved residues are shaded in red, and similar amino acids are highlighted by blue frames. Residues forming conserved ionic pairs in the ACPI structures are indicated by green and red arrows respectively. Binding sites of Chl 301-312 are indicated by green stars.

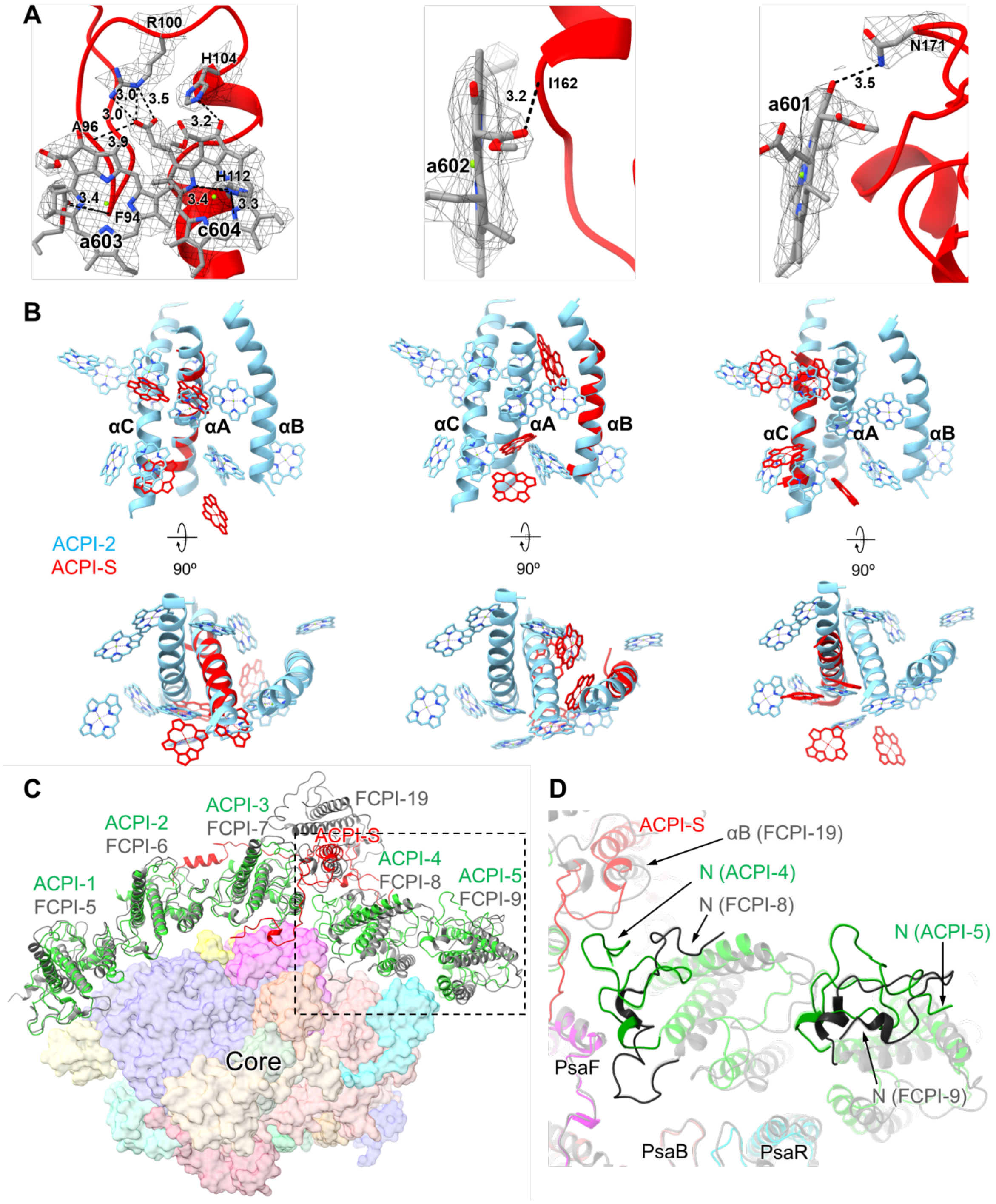

Supplemental Figure S13. Pigment association and location of ACPI-S (Supports Figure 3). A, Interactions between pigments and amino acid residues in ACPI-S. **B**, Structural comparison between the transmembrane helix and coordinated Chls of ACPI-S and ACPI-2. Three transmembrane helices of ACPI-2 are labeled as αA, αB and αC, respectively. **C**, Comparison of the binding positions of ACPIs (1-5) and their corresponding FCPIs (5-9) in diatoms, showing the shift of ACPI-4/5 due to the presence of ACPI-S (red). A zoom-in view of the boxed area is shown in **D**. **D**, Enlarged view of the boxed area in **C**. The N-terminal loops of ACPI-4/5 and FCPI-8/9 are highlighted and indicated by arrows. The transmembrane helix αB of FCPI-19 is indicated by an arrow.

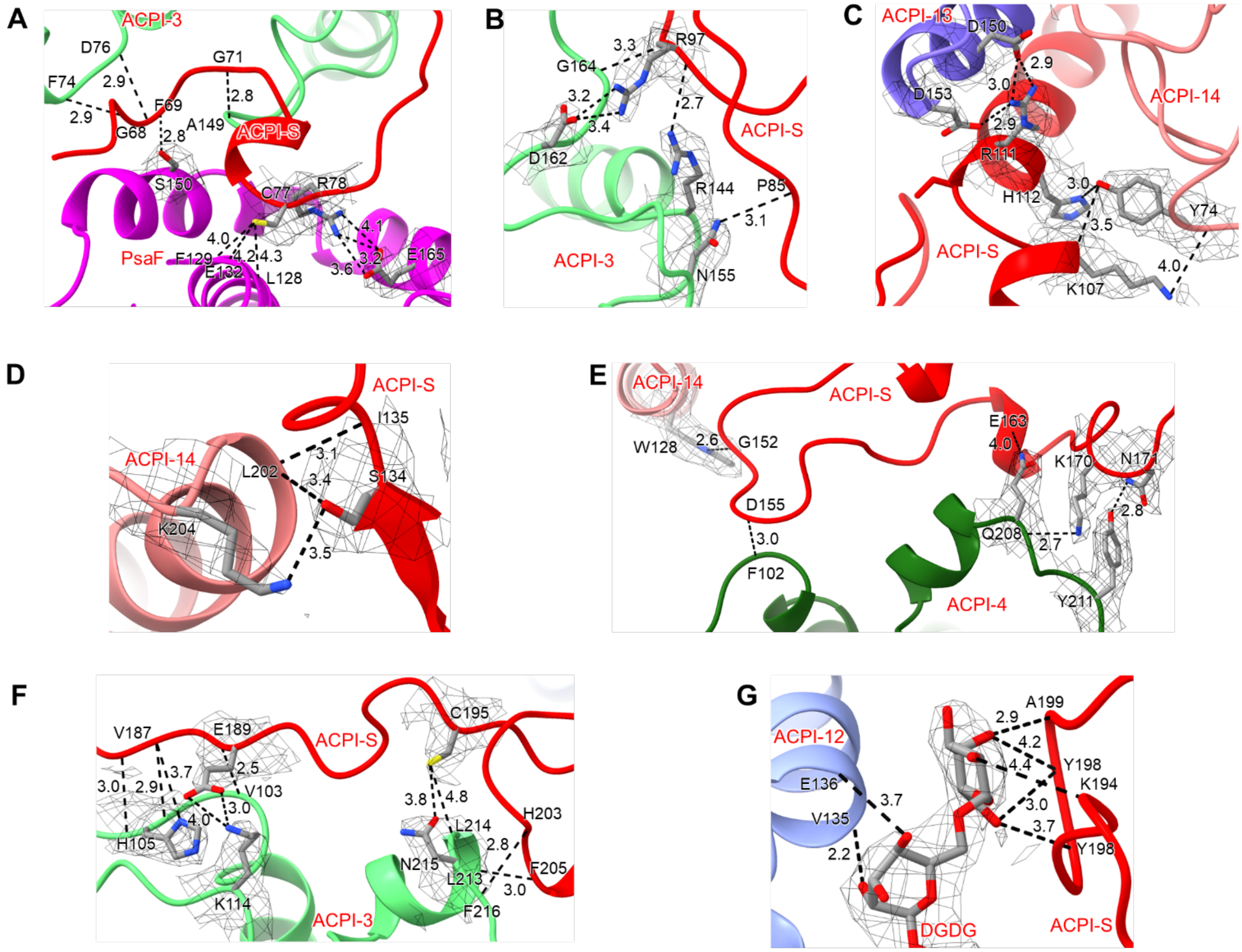

Supplemental Figure S14. Interactions between ACPI-S and surrounding subunits (Supports Figure 3). A-B, Interactions between ACPI-S and PsaB/ACPI-3 at the stromal side. **C**, Interactions between ACPI-S and ACPI-13/ACPI-14 at the stromal side. **D-E**, Interactions between ACPI-S and ACPI-4/ACPI-14 at the lumenal side. **F**, Interactions between ACPI-S and ACPI-3 at the lumenal side. **G**, Interactions between ACPI-S and ACPI-12 mediated by the lipid DGDG at the lumenal side. Interactions are indicated by black dashed lines with distances labeled in Å. The density maps of related residues are shown.

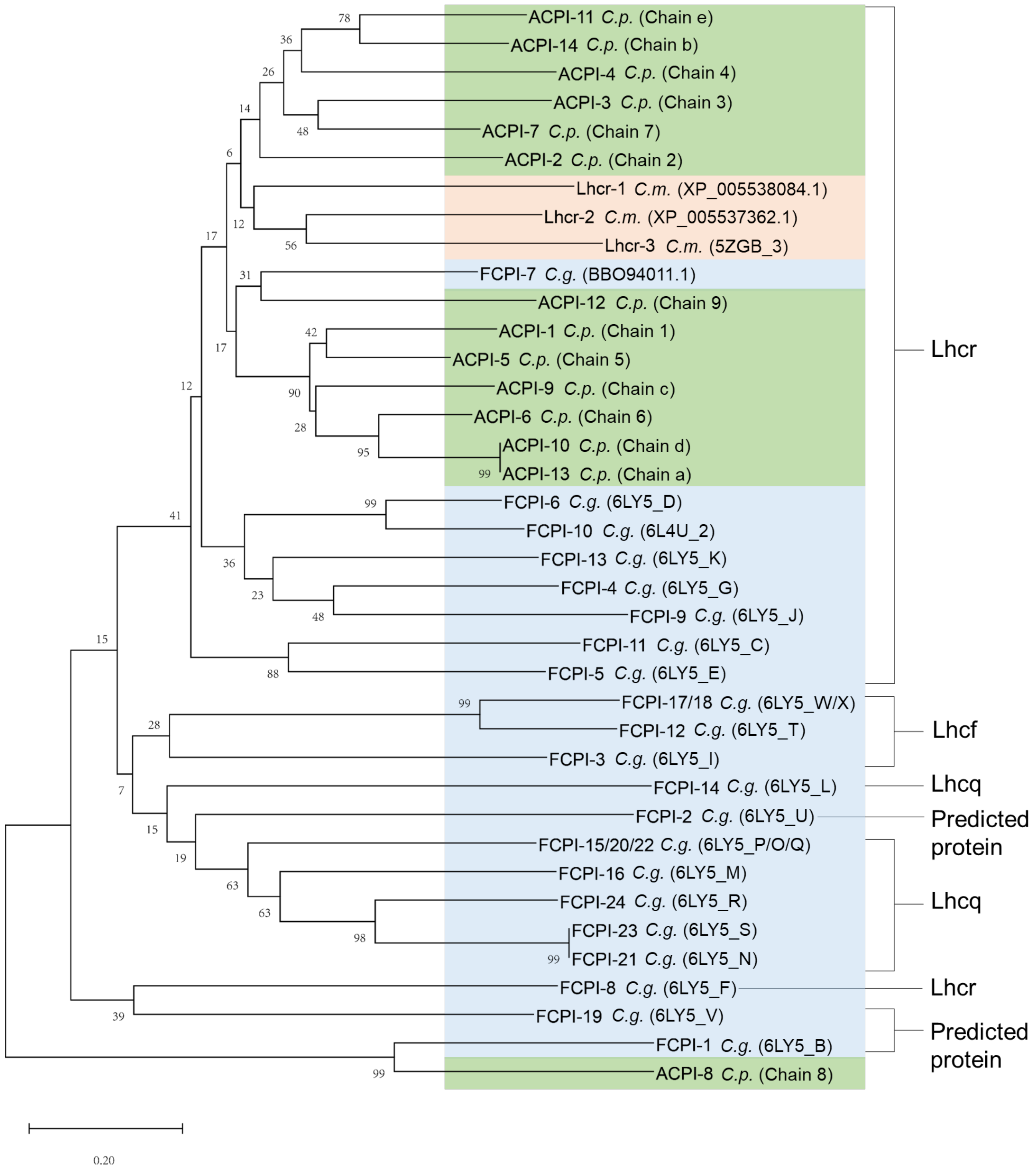

Supplemental Figure S15. Phylogenetic tree of LHCIs in the cryptophyte *C. placoidea* (*C.p.*) (green), red alga *C. merolae* (*C.m.*) (orange), and diatom *C. gracilis* (*C.g.*) (blue) (Supports Figure 3). The neighbor-joining tree is based on the amino acid sequences of LHCIs. The tree was built with the Poisson model using 107 amino acid residues, and a bootstrap test (1000 replicates) was conducted. The percentage of replicate trees in which the associated taxa clustered together in the bootstrap test are shown next to the branches.

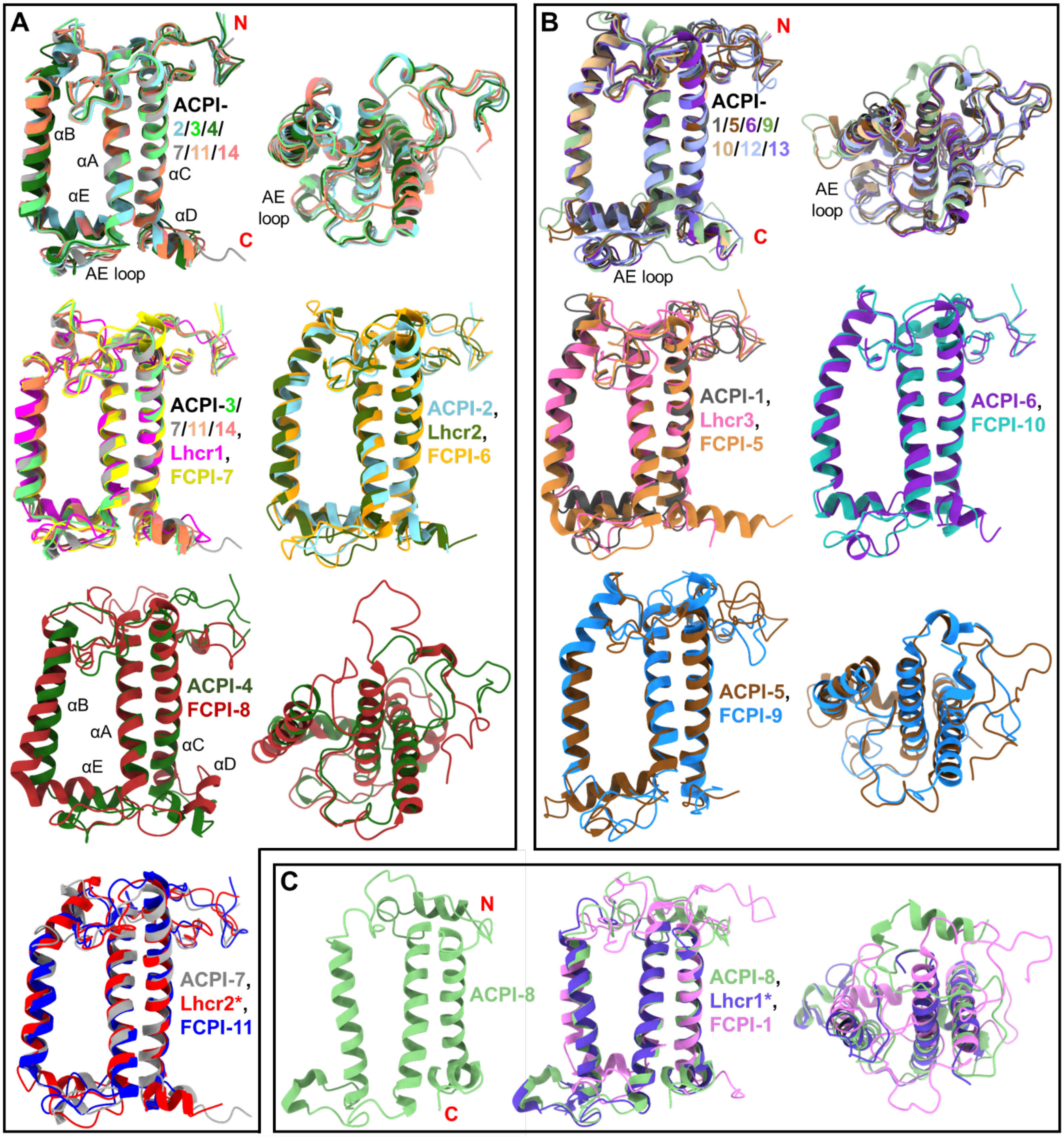

Supplemental Figure S16. Structural comparison of the ACPI structures and between ACPIs and Lhcrs from the red alga *C. merolae* and FCPIs from the diatom *C. gracilis* (Supports Figure 3). A, Superposition of ACPI-2/3/4/7/11/14 (group I) and their comparisons with Lhcrs and FCPIs. **B**, Superposition of ACPI-1/5/6/9/10/12/13 (group II) and their comparisons with Lhcrs and FCPIs. **C**, Structure of ACPI-8 (group III) and its comparison with Lhcr1* and FCPI-1. The colors of ACPI subunits are the same as those in Figure 1. Color codes for Lhcrs and FCPIs: Lhcr1, magenta; Lhcr2, olive green; Lhcr3, pink; Lhcr1*, violet; Lhcr2*, red; FCPI-1, slate blue; FCPI-5, light brown; FCPI-6, orange; FCPI-7, yellow; FCPI-8, brown; FCPI-9, dodger blue; FCPI-10, sea green; FCPI-11, blue.

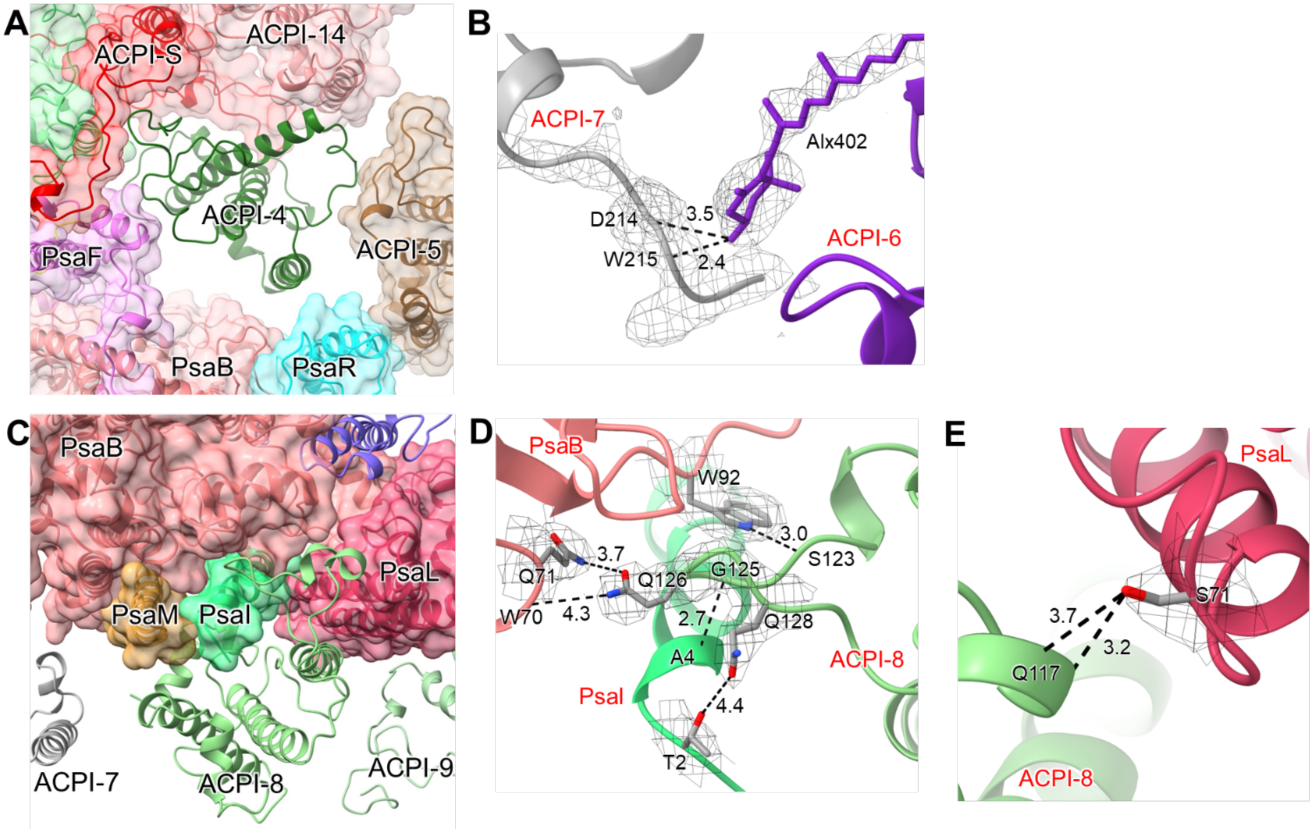

Supplemental Figure S17. Location of ACPI-4 viewed from the stromal side (A), interactions of the C-terminal loop of ACPI-7 with ACPI-6 (B), and interactions between ACPI-8 and PSI core subunits at the lumenal side (C-E) (Supports Figures 1 and 3). Interactions are indicated by black dashed lines with distances labeled in Å. The density maps of related residues are shown.

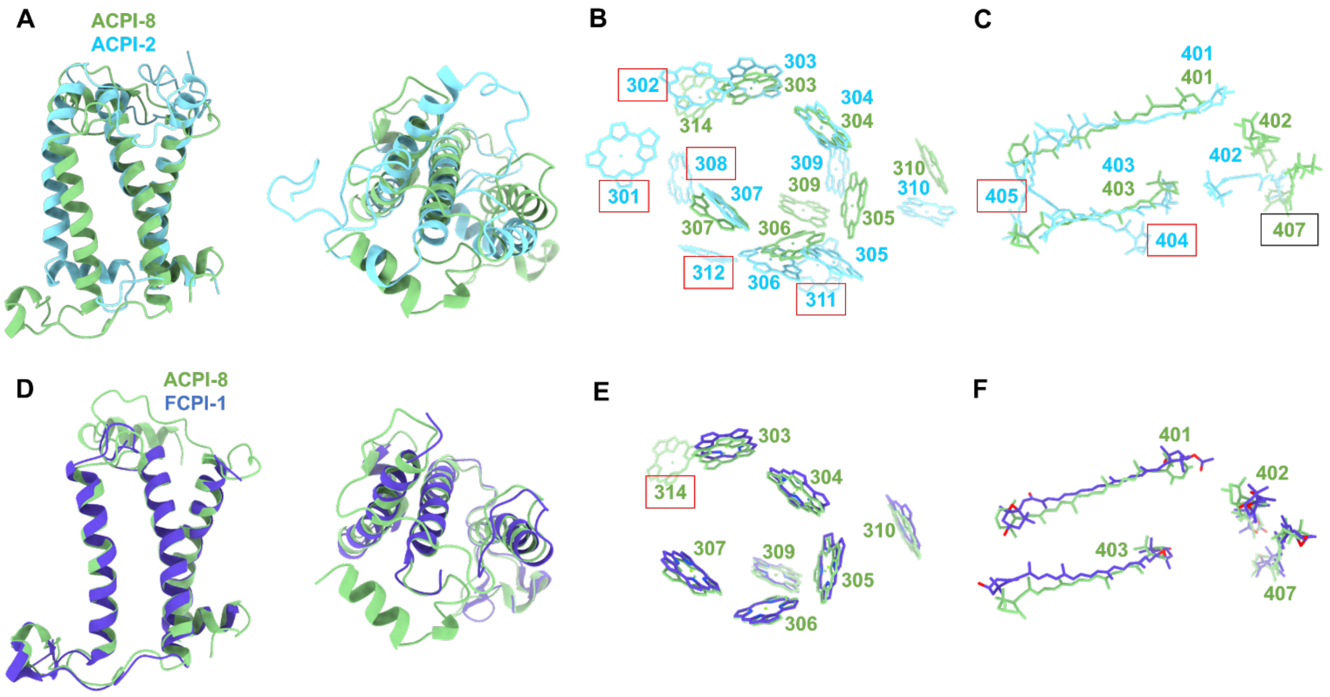

Supplemental Figure S18. Comparisons of pigment arrangements in ACPI-8 with ACPI-2 and diatom FCPI-1 (Supports Figure 3). A, Structural comparison of proteins between ACPI-8 (green) and ACPI-2 (cyan). **B**, Comparison of Chl sites in ACPI-8 (green) and ACPI-2 (cyan). The Chl sites missing in ACPI-8 are indicated by red boxes. **C**, Comparison of Car sites in ACPI-8 (green) and ACPI-2 (cyan). The Car sites missing in ACPI-8 are indicated by red boxes. The new Car site 407 in ACPI-8, which is missing in other ACPIs, is indicated by a black box. **D**, Structural comparison of proteins between ACPI-8 (green) and FCPI-1 (blue). **E**, Comparison of Chl sites in ACPI-8 (green) and FCPI-1 (blue). The new Chl site 314 in ACPI-8, which is absent in FCPI-1, is indicated by a red box. **F**, Comparison of Car sites in ACPI-8 (green) and FCPI-1 (blue).

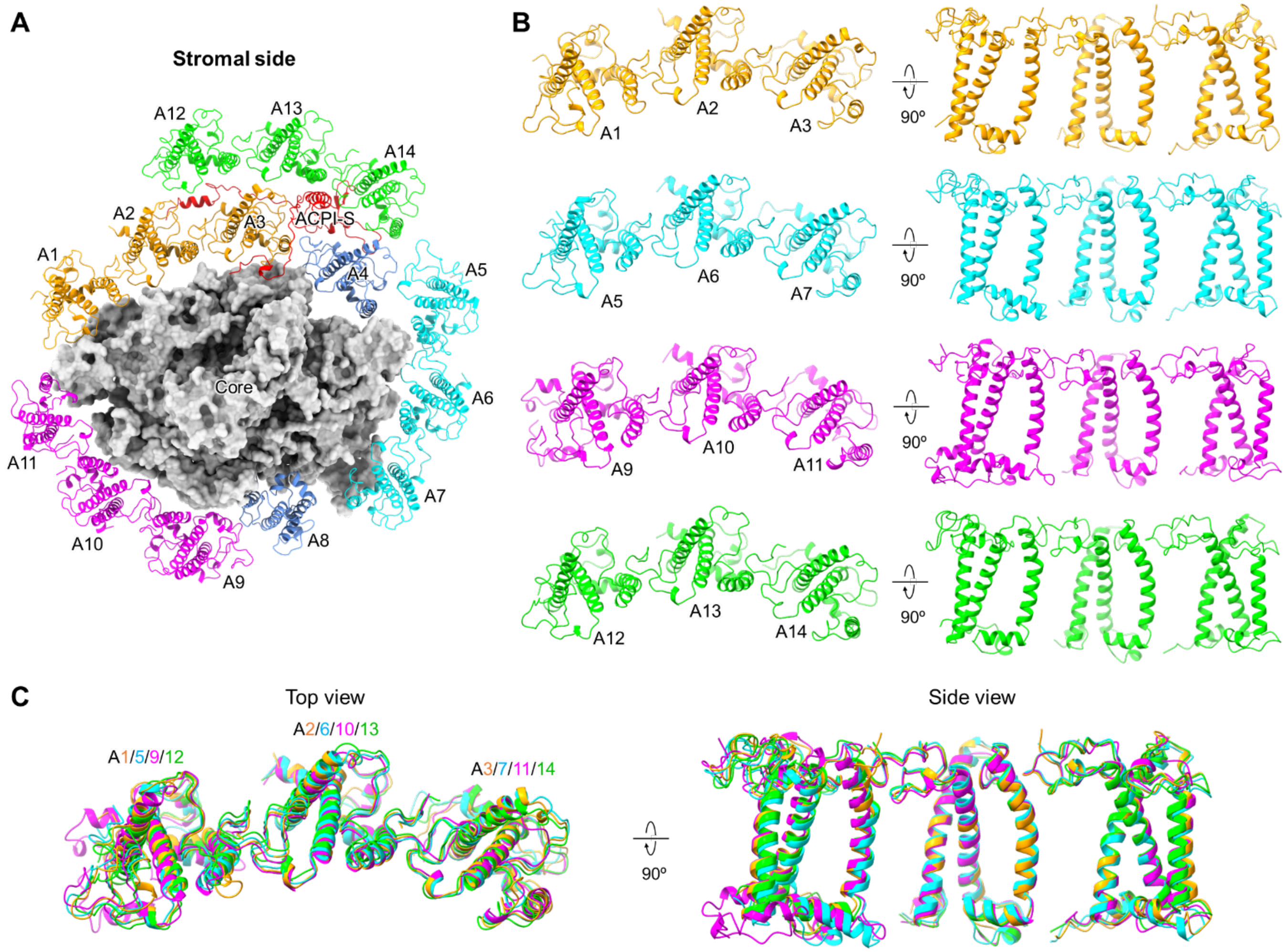

Supplemental Figure S19. The structures of three adjacent ACPIs used as the building modules (Supports Figures 1 and 3). A, The locations of ACPI trimers viewed from the stromal side. A1-A2-A3 (orange); A5-A6-A7 (cyan); A9-A10-A11 (magenta); A12-A13-A14 (green). A1-A14 indicate ACPI1-14, respectively. **B**, The structures of ACPI trimers (ACPI-1/2/3, ACPI-5/6/7, ACPI-9/10/11, and ACPI-12/13/14). **C**, Superimposition of the four ACPI trimers.

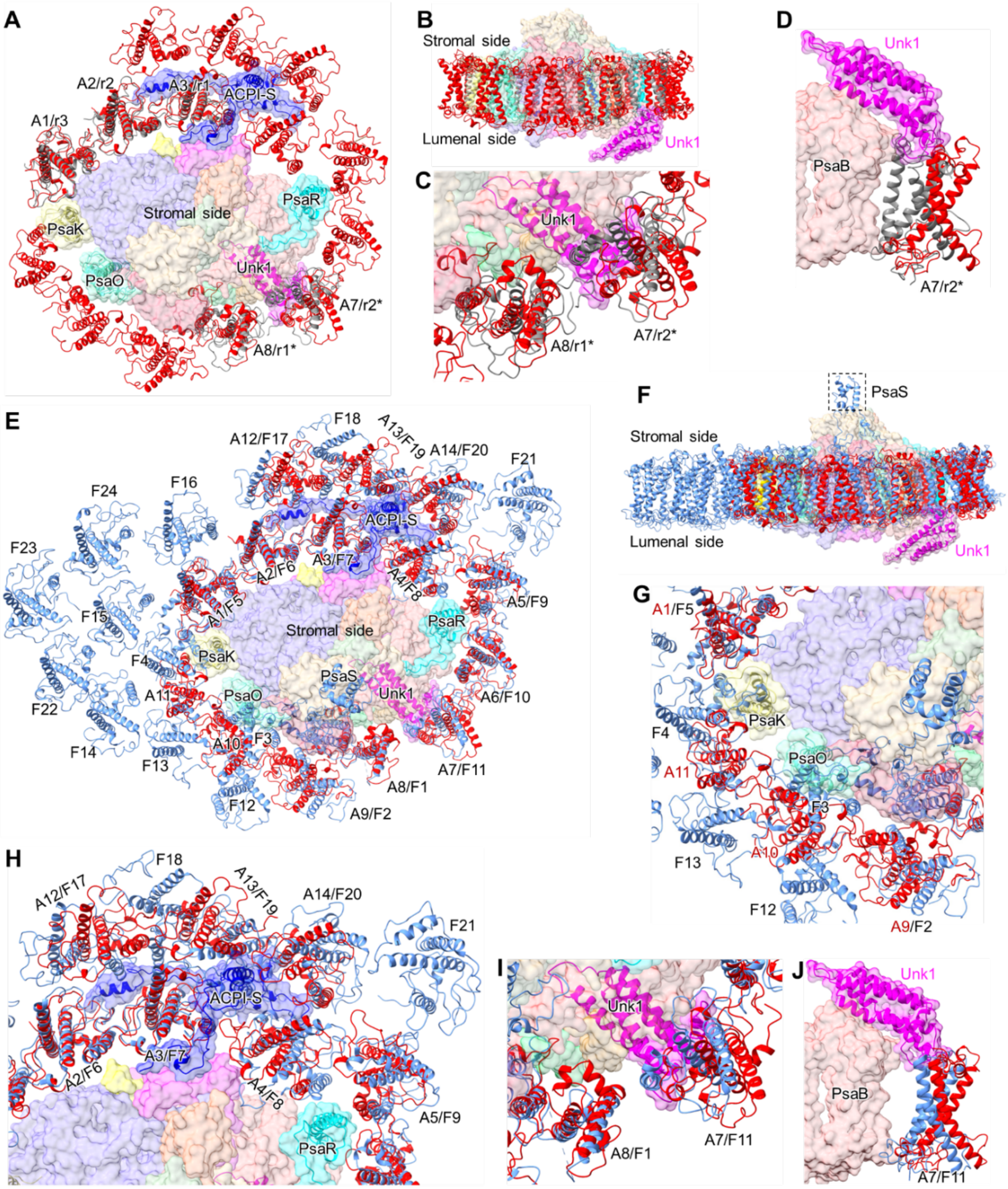

Supplemental Figure S20. Structural comparison of PSI–ACPI of the cryptophyte *C. placoidea* with that of the red alga *C. merolae* and the diatom *C. gracilis* (Supports Figures 3 and 5). A, Superposition of the PSI–ACPI structure (red) with the PSI–LHCR (gray) structure of red algae. PsaK, PsaO, PsaR, and the newly identified subunits ACPI-S and Unk1 are labeled. A1, A2, A3, A7 and A8 indicate ACPI-1/2/3/7/8, respectively. r1, r2, r3, r1* and r2* indicate Lhcr1/2/3/1*/2*, respectively. **B**, Side view of panel **A** with Unk1 indicated. **C-D**, Shift of ACPI-7 (red) compared with Lhcr2* (gray) viewed from the stromal surface (**C**) and side (**D**). **E**, Superposition of the PSI–ACPI (red) structure with PSI–FCPI (cornflower blue) structure of diatoms. PsaK, PsaO, PsaR, PsaS, ACPI-S, and Unk1 are labeled. A1-A14 and F1-F24 indicate ACPI1-14 and FCPI1-24, respectively. **F**, Side view of panel **E** with Unk1 indicated. PsaS, which is absent in PSI–ACPI, is indicated by a black square. **G-H**, Structures in **E** are enlarged to show more detail. **I-J**, Shift of ACPI-7 (red) compared with FCPI-1 (cornflower blue) viewed from the stromal surface (**I**) and side (**J**).

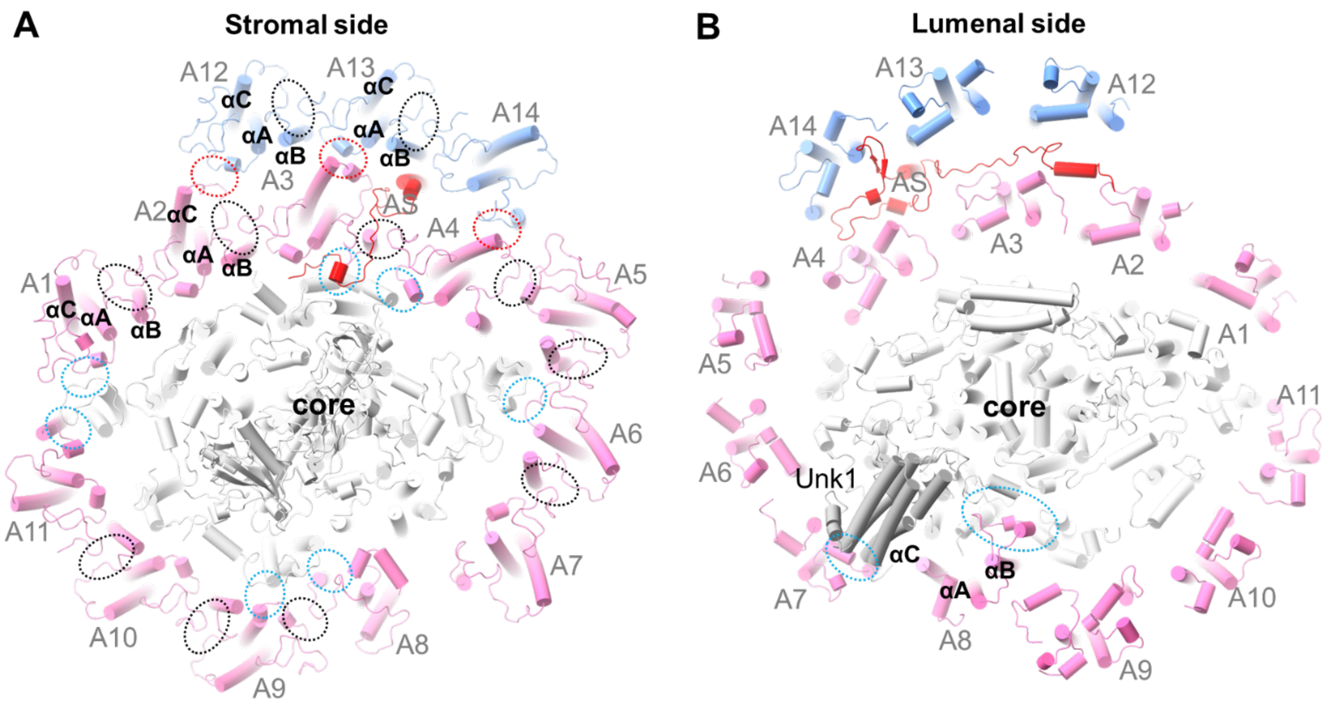

Supplemental Figure S21. Interactions between ACPIs in the same layer (black circle), between outer ACPIs and inner ACPIs (red circle), and between inner ACPIs and PSI core (blue circle) at the stromal side (A) and lumenal side (B) (Supports Figures 1 and 3). A1-A14 represent ACPI-1 to ACPI-14. AS represents ACPI-S.

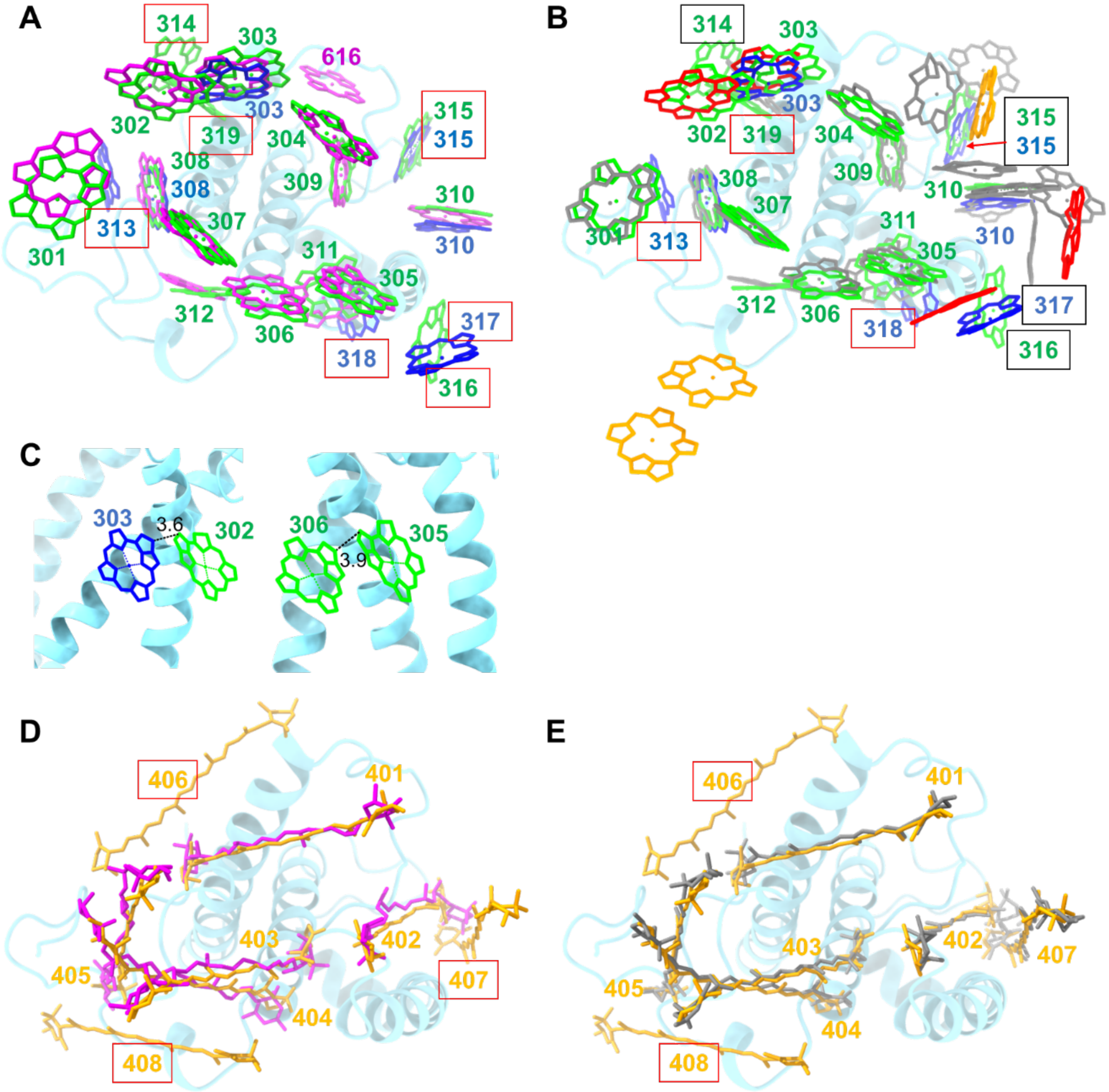

Supplemental Figure S22. Comparisons of pigment arrangements in ACPIs with those in red alga Lhcrs and diatom FCPIs (Supports Figures 4 and 5). **A**, Comparison of Chl sites in ACPIs (Chl *a*, green; Chl *c*, blue) and Lhcrs (magenta). Seven new Chl sites in ACPIs that are missing in Lhcrs are indicated by red boxes. The Chl616 in Lhcr2 that is missing in ACPIs is labeled. **B**, Comparison of Chl sites in ACPIs (Chl *a*, green; Chl *c*, blue) and FCPIs (Chl *a*, gray; Chl *c*, orange; Chl *a* or Chl *c*, red). The new Chl sites 313, 318 and 319 in ACPIs that are missing in FCPIs are indicated by red boxes. The similar Chl sites of ACPIs with FCPIs are indicated by black boxes. **C**, Chl *a*-*c* and chl *a*-*a* pairs in ACPI-2. Interactions are indicated by black dashed lines with distances labeled in Å. **D**, Comparison of Car-binding sites in ACPIs (orange) and Lhcrs (magenta). Three new Car sites in ACPIs that are missing in Lhcrs are indicated by red boxes. **E**, Comparison of Car sites in ACPIs (orange) with FCPI-1 and FCPI-7 (gray). The new Car site 406 and 408 in ACPIs that are missing in FCPIs is indicated by a red box.

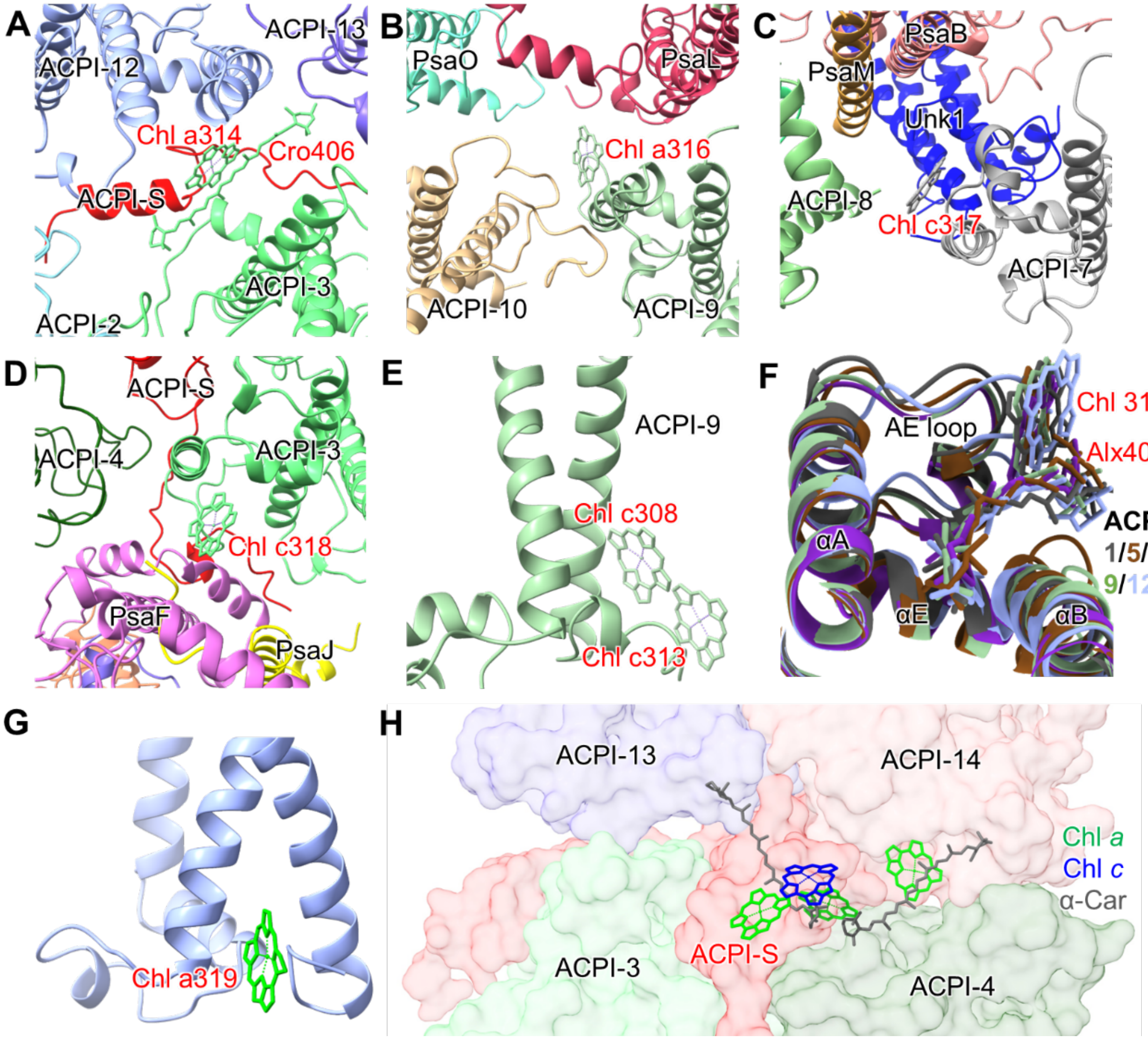

Supplemental Figure S23. The locations of Chl *a*314 and Cro406 (A), Chl *a*316 (B), Chl *c*317 (C), Chl *c*318 (D), Chl *a*313 (E), Chl 315 (F), Chl *a*319 (G) and pigments (Chl *a*: green, Chl *c*: blue, α-Car: gray) of ACPI-S (H) (Supports Figure 4).

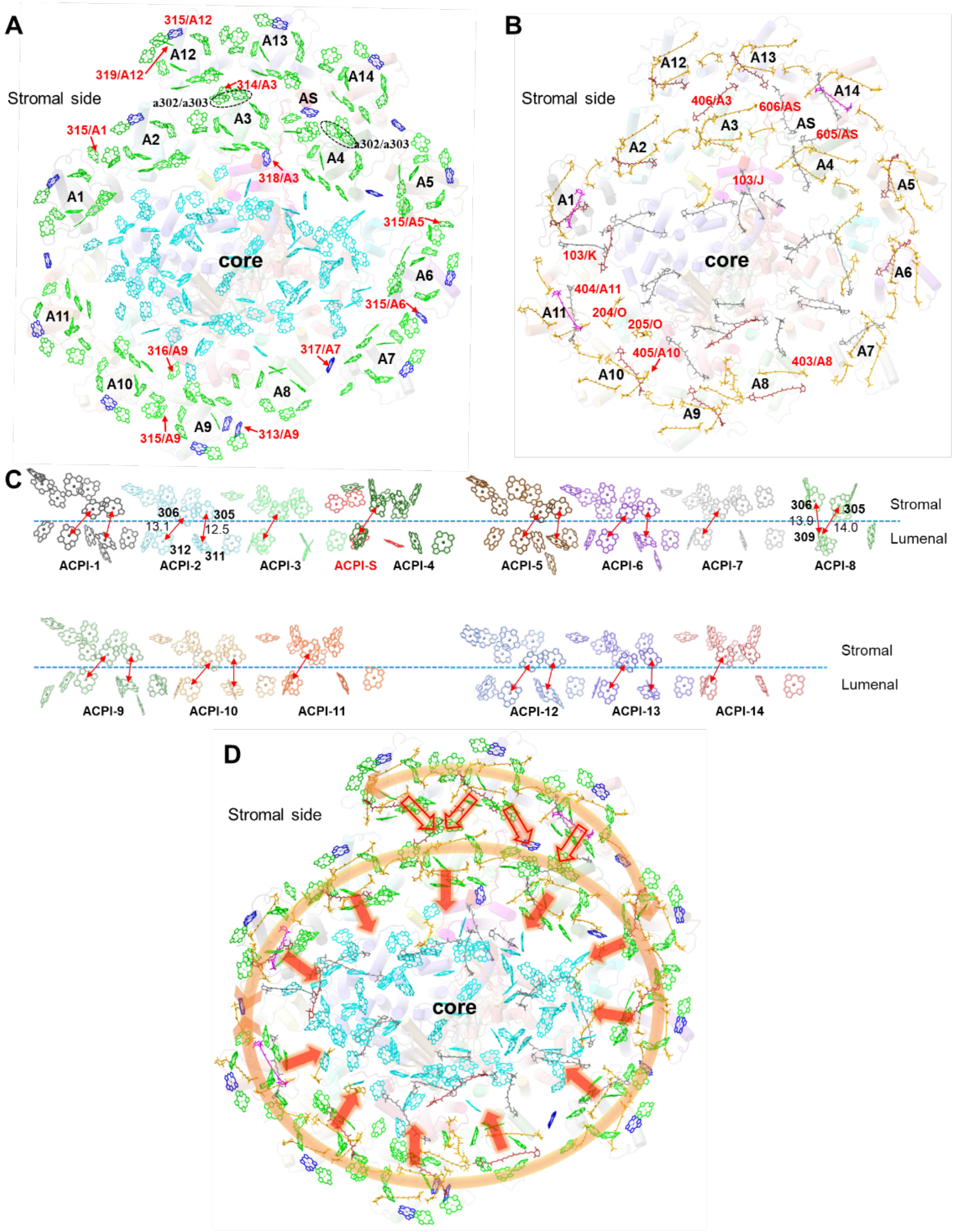

Supplemental Figure S24. Arrangement of the pigments in PSI–ACPI (Supports Figure 4). A, Arrangement of all Chls in PSI–ACPI. Chls 313-319 are labeled. Chl *a*302/*a*303 homo-pairs are indicated by ovals. A1-A14 represent ACPI-1 to ACPI-14. AS represents ACPI-S. **B**, Arrangement of all carotenoids in PSI–ACPI. The unique quenching sites are labeled. **C**, Side view of Chl arrangement in all ACPIs. Blue dotted line divides the Chls into the stromal and lumenal layers. Red double-headed arrows indicate the close interactions between the Chl *a*305/*a*306 pair and Chl *a*311/*a*312 within ACPIs (1-7, 9-14) and interactions between the Chl *a*305/*a*306 pair and Chl *a*309 within ACPI-8. The Chls forming close interactions between the stromal and lumenal layers are labeled in ACPI-2 as an example and ACPI-8 as well as the distances. **D**, Arrangement of all pigments in PSI–ACPI. The energy transfer between the ACPIs within the same layer (orange arrow), from outer to inner ACPIs (red hollow arrows), and from inner ACPIs to the PSI core (red solid arrow) are indicated. Chl *a* in ACPIs, Chl *c* in ACPIs, Chl *a* in the core, alloxanthin, crocoxanthin, monadoxanthin, and α-carotene are colored green, blue, cyan, orange, brown, magenta, and gray, respectively.

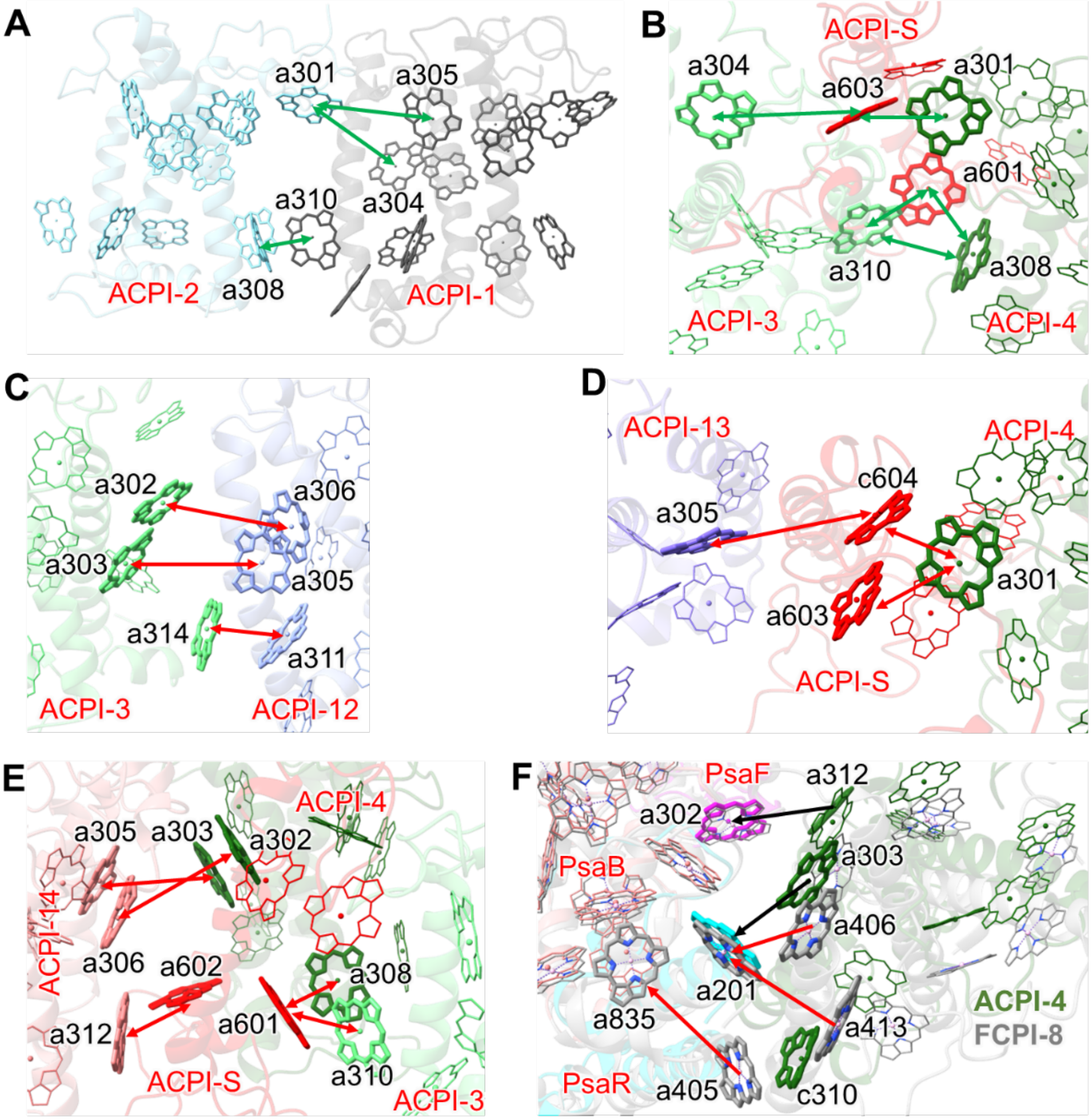

Supplemental Figure S25. EET between ACPIs and from ACPI to the PSI core (Supports Figure 4). A, EET between ACPI-1 and ACPI-2 is shown by green arrows. **B**, EET between ACPI-3 and ACPI-4 mediated by ACPI-S is shown by green arrows. **C**, EET between inner ACPI-3 and outer ACPI-12 is shown by red arrows. **D**, EET between inner ACPI-4 and outer ACPI-13 mediated by ACPI-S is shown by red arrows. **E**, EET between outer ACPI-14 and inner ACPI-3/4 mediated by ACPI-S is shown by red arrows. **F**, Comparison of EET from ACPI-4 to the PSI core (black arrows) and its counterpart (FCPI-8) in diatoms to the PSI core (red arrows). Chls of diatom PSI–FCPI are colored gray. Chls in PsaB, PsaF, PsaR and ACPI-4 are colored pink, magenta, cyan and green, respectively.

Supplemental Table S1. Cryo-EM data collection, refinement, and validation statistics.

	**PSI–14ACPI (EMDB-33659) (PDB-7Y7B)**	**PSI–11ACPI (EMDB-33683) (PDB-7Y8A)**
**Data Collection and Processing**		
Magnification	81,000	81,000
Voltage (kV)	300	300
Electron exposure (e- /Å2)	50	50
Defocus range (um)	-1.2~-2.2	-1.2~-2.2
Pixel size (Å)	0.53	0.53
Symmetry imposed	C1	C1
Initial particle images (no.)	1,199,190	1,199,190
Final particle images (no.)	133,521	118,810
Map resolution (Å)	2.66	2.71
FSC threshold	0.143	0.143
**Refinement**		
Initial model used (PDB code)	5ZGB	PSI–14ACPI
Model resolution (Å)	2.66	2.71
FSC threshold	0.143	0.143
Map sharpening *B* factor ($Å^2$)	-85.5	-79.9
Model composition		
Non-hydrogen atoms	60,514	53,403
Protein residues	5083	4519
Ligands	417	357
B factors ($Å^2$)		
Protein	46.1	68.2
Ligand	47.3	68.7
R.m.s. deviations		
Bond lengths (Å)	0.012	0.014
Bond angles (º)	1.318	1.356
Validation		
MolProbity score	1.28	1.27
Clashscore	3.10	3.22
Poor rotamers (%)	0.27	0.42
Ramachandran plot		
Favored (%)	96.91	97.16
Allowed (%)	3.05	2.78
Disallowed (%)	0.04	0.07

Supplemental Table S2. Pigments and cofactors in cryptophyte PSI–ACPI.

Subunit	Traced residues	Chls	Cars	Lipids	Others
PsaA	741 (12-752)	45 a	4 α-Car, 1 Alx	2 PG	1 SF4, 1 PQN
PsaB	731 (3-733)	41 a	6 α-Car, 1 Alx	2 PG, 1 MGDG, 1 DGDG	1 PQN
PsaC	80 (2-81)				2 SF4
PsaD	137 (4-140)				
PsaE	61 (2-62)				
PsaF	160 (24-183)	3 a	1 α-Car	1 PG	
PsaI	33 (1-33)	1 a	1 α-Car		
PsaJ	42 (1-42)	1 a	2 α-Car	1 MGDG	
PsaK	78 (10-87)	2 a	1 α-Car, 1Cro		
PsaL	153 (2-154)	3 a	2 α-Car, 1Cro		
PsaM	30 (1-30)		1 α-Car	1 MGDG	
PsaO	99 (56-154)	3 a	2 Alx	1 SQDG	
PsaR	92 (42-133)	1 a	1 α-Car, 1 Alx		
Unk1	162 (1-162)				
ACPI-1	179 (41-219)	12 a, 1 c	3 Alx, 1 Cro, 1 Mon		
ACPI-2	168 (43-210)	11 a, 1 c	4 Alx, 1 Cro	3 PG, 1 MGDG	
ACPI-3	180 (42-221)	12 a, 1 c	5 Alx, 1 Cro	1 PG, 2 MGDG, 1 SQDG	
ACPI-4	176 (41-216)	10 a, 1 c	4 Alx, 1 α-Car	1 PG	
ACPI-5	185 (41-225)	12 a, 1 c	4 Alx, 1 Cro	1 PG	
ACPI-6	173 (40-212)	11 a, 2 c	4 Alx, 1 Cro	1 PG	
ACPI-7	177 (41-217)	10 a, 2 c	4 Alx, 1 α-Car	2 PG	
ACPI-8	175 (53-227)	8 a	3 Alx, 1 Cro	1 PG, 1 MGDG	
ACPI-9	187 (1-187)	12 a, 3c	5 Alx, 1 Cro	2 PG	
ACPI-10	171 (42-212)	11 a, 1 c	4 Alx, 1 Cro	1 PG	
ACPI-11	168 (41-208)	9 a, 2 c	3 Alx, 1 α-Car, 1 Mon	1 PG	
ACPI-12	178 (41-218)	13 a, 1 c	4 Alx, 1 Cro	1 DGDG	
ACPI-13	171 (42-212)	11 a, 1 c	4 Alx, 1 Cro	3 PG	
ACPI-14	176 (43-218)	9 a, 2 c	3 Alx, 1 α-Car, 1 Mon	1 PG	
ACPI-S	153 (65-218)	3 a, 1 c	2 α-Car		
PSI–ACPI		254 a, 20 c	59 Alx, 25 α-Car, 12 Cro, 3 Mon	23 PG, 7 MGDG, 2 DGDG, 2 SQDG	2 PQN, 3 SF4

DGDG, digalactosyldiacyl glycerol; MGDG, monogalactosyldiacyl glycerol; PG, phosphatidyl glycerol; SQDG, sulfoquinovosyldiacyl glycerol; PQN, phylloquinone; SF4, sulphur–iron cluster.

Supplemental Table S3. Comparison of the protein subunits in PSI–LHCI of cryptophytes, cyanobacteria, red algae, diatom, green algae, and land plants. √: present of the subunit.

Subunit	Cryptophyte	Cyanobacteria	Red algae	Diatom	Green algae	Land plants
PsaA	√	√	√	√	√	√
PsaB	√	√	√	√	√	√
PsaC	√	√	√	√	√	√
PsaD	√	√	√	√	√	√
PsaE	√	√	√	√	√	√
PsaF	√	√	√	√	√	√
PsaG					√	√
PsaH					√	√
PsaI	√	√	√	√	√	√
PsaJ	√	√	√	√	√	√
PsaK	√	√	√		√	√
PsaL	√	√	√	√	√	√
PsaM	√	√	√	√	√	
PsaN					√[a]	√
PsaO	√		√		√	√
PsaR	√			√		
PsaS				√		
PsaX		√				
Unk1	√					
ACPI-S	√					
LHCI	11 or 14	0	3 or 5	24	6, 8, 9 or 10	4

a: missing in the reported purified green algal PSI-LHCI structures, likely due to weak association.

Supplemental Table S4. Pigment binding sites in ACPIs.

Sites	ACPI -1	ACPI -2	ACPI -3	ACPI -4	ACPI -5	ACPI -6	ACPI -7	ACPI -8	ACPI -9	ACPI -10	ACPI -11	ACPI -12	ACPI -13	ACPI -14
301	Chl *a*	Chl *a*	Chl *a*	Chl *a*	Chl *a*	Chl *a*	Chl *a*		Chl *a*	Chl *a*	Chl *a*	Chl *a*	Chl *a*	Chl *a*
302	Chl *a*	Chl *a*	Chl *a*	Chl *a*	Chl *a*	Chl *a*	Chl *a*		Chl *a*	Chl *a*	Chl *a*	Chl *a*	Chl *a*	Chl *a*
303	Chl *c*	Chl *c*	Chl *a*	Chl *a*	Chl *c*	Chl *c*	Chl *c*	Chl *a*	Chl *c*	Chl *c*	Chl *c*	Chl *c*	Chl *c*	Chl *c*
304	Chl *a*	Chl *a*	Chl *a*	Chl *a*	Chl *a*	Chl *a*	Chl *a*	Chl *a*	Chl *a*	Chl *a*	Chl *a*	Chl *a*	Chl *a*	Chl *a*
305	Chl *a*	Chl *a*	Chl *a*	Chl *a*	Chl *a*	Chl *a*	Chl *a*	Chl *a*	Chl *a*	Chl *a*	Chl *a*	Chl *a*	Chl *a*	Chl *a*
306	Chl *a*	Chl *a*	Chl *a*	Chl *a*	Chl *a*	Chl *a*	Chl *a*	Chl *a*	Chl *a*	Chl *a*	Chl *a*	Chl *a*	Chl *a*	Chl *a*
307	Chl *a*	Chl *a*	Chl *a*	Chl *a*	Chl *a*	Chl *a*	Chl *a*	Chl *a*	Chl *a*	Chl *a*	Chl *a*	Chl *a*	Chl *a*	Chl *a*
308	Chl *a*	Chl *a*	Chl *a*	Chl *a*	Chl *a*	Chl *a*	Chl *a*		Chl *c*	Chl *a*	Chl *a*	Chl *a*	Chl *a*	Chl *a*
309	Chl *a*	Chl *a*	Chl *a*	Chl *a*	Chl *a*	Chl *a*	Chl *a*	Chl *a*	Chl *a*	Chl *a*	Chl *a*	Chl *a*	Chl *a*	Chl *a*
310	Chl *a*	Chl *a*	Chl *a*	Chl *c*	Chl *a*	Chl *a*	Chl *a*	Chl *a*	Chl *a*	Chl *a*	Chl *c*	Chl *a*	Chl *a*	Chl *c*
311	Chl *a*	Chl *a*			Chl *a*	Chl *a*			Chl *a*	Chl *a*		Chl *a*	Chl *a*	
312	Chl *a*	Chl *a*	Chl *a*	Chl *a*	Chl *a*	Chl *a*	Chl *a*		Chl *a*	Chl *a*	Chl *a*	Chl *a*	Chl *a*	Chl *a*
313									Chl *c*					
314			Chl *a*					Chl *a*						
315	Chl *a*				Chl *a*	Chl *c*			Chl *a*			Chl *a*		
316									Chl *a*					
317							Chl *c*							
318			Chl *c*											
319												Chl *a*		
401	Alx	Alx	Alx	Alx	Alx	Alx	Alx	Cro	Alx	Alx	Alx	Alx	Alx	Alx
402	Alx	Alx	Alx	Alx	Alx	Alx	Alx	Alx	Alx	Alx	Alx	Alx	Alx	Alx
403	Mon	Alx	Alx	Alx	Alx	Alx	Alx	Alx	Alx	Alx	Mon	Alx	Alx	Mon
404	Cro	Cro	Alx	α-Car	Cro	Cro	α-Car		Cro	Cro	α-Car	Cro	Cro	α-Car
405	Alx	Alx	Alx	Alx	Alx	Alx	Alx		Alx	Alx	Alx	Alx	Alx	Alx
406			Cro											
407								Alx						
408									Alx					

Supplemental Table S5. Possible excitation energy transfer pathways from ACPIs to the PSI core. The pathways are based on the Mg-Mg distances in Chl *a*. The numbers 1 to 14 represent ACPI-1 to ACPI-14. The uppercase letters outside of the brackets represent PSI core subunits. The uppercase letters in brackets represent the stromal side (S) and lumenal side (L).

Possible pathway	Pigments	Mg-Mg distance (Å)	Location
1-K (L)	ACPI-1: Chl a312→PsaK: Chl a102	12.8	Lumenal
1-A (S)	ACPI-1: Chl a306→PsaA: Chl a821	17.5	Stromal
2-A (L)	ACPI-2: Chl a312→PsaA: Chl a817	12.9	Lumenal
2-A (S)	ACPI-2: Chl a305→PsaA: Chl a809	25.2	Stromal
13-3-J (L)	ACPI-13: Chl a312→ACPI-3: Chl a314	19.1	Lumenal
	ACPI-3: Chl a312→PsaJ: Chl a101	11.5	
13-12-3-J (L)	ACPI-13: Chl a308→ACPI-12: Chl a310	8.3	Lumenal
	ACPI-12: Chl a311→ACPI-3: Chl a314	10.3	
	ACPI-3: Chl a312→PsaJ: Chl a101	11.5	
13-12-3-F (S)	ACPI-13: Chl a301→ACPI-12: Chl a304	17.0	Stromal
	ACPI-12: Chl a306→ACPI-3: Chl a302	15.8	
	ACPI-3: Chl a306→PsaF: Chl a302	19.3	
13-S-4-R (S)	ACPI-13: Chl a305→ACPI-S: Chl c604	20.1	Stromal
	ACPI-S: Chl c604→ACPI-4: Chl a301	10.8	
	ACPI-4: Chl a305→PsaR: Chl a201	11.9	
13-14-S-3-J (L)	ACPI-13: Chl a310→ACPI-14: Chl a308	8.1	Lumenal
	ACPI-14: Chl a312→ACPI-S: Chl a602	13.5	
	ACPI-S: Chl a601→ACPI-3: Chl a310	9.1	
	ACPI-3: Chl a312→PsaJ: Chl a101	11.5	
13-14-S-4-F (L)	ACPI-13: Chl a310→ACPI-14: Chl a308	8.1	Lumenal
	ACPI-14: Chl a312→ACPI-S: Chl a602	13.5	
	ACPI-S: Chl a601→ACPI-4: Chl a308	10.4	
	ACPI-4: Chl a312→PsaF: Chl a303	13.4	
13-14-4-R (S)	ACPI-13: Chl a304→ACPI-14: Chl a301	16.6	Stromal
	ACPI-14: Chl a305→ACPI-4: Chl a303	13.9	
	ACPI-4: Chl a305→PsaR: Chl a201	11.9	
5-4-F (L)	ACPI-5: Chl a312→ACPI-4: Chl a310	15.5	Lumenal
	ACPI-4: Chl a312→PsaF: Chl a303	13.4	
5-R (S)	ACPI-5: Chl a305→PsaR: Chl a201	23.7	Stromal
5-6-B (L)	ACPI-5: Chl a310→ACPI-6: Chl a308	8.1	Lumenal
	ACPI-6: Chl a311→PsaB: Chl a814	19.0	
5-6-B (S)	ACPI-5: Chl a304→ACPI-6: Chl a301	16.4	Lumenal
	ACPI-6: Chl a305→PsaB: Chl a820	13.9	
7-B (L)	ACPI-7: Chl a312→PsaB: Chl a841	16.4	Lumenal
7-B (S)	ACPI-7: Chl a306→PsaB: Chl a811	19.3	Stromal
8-I (L)	ACPI-8: Chl a309→PsaI: Chl a102	11.4	Lumenal
8-7-B (S)	ACPI-8: Chl a307→ACPI-7: Chl c317	17.5	Stromal
	ACPI-7: Chl a306→PsaB: Chl a811	19.3	
8-9-L (S)	ACPI-8: Chl a304→ACPI-9: Chl a301	21.0	Stromal
	ACPI-9: Chl a306→PsaL: Chl a202	16.0	
9-O (L)	ACPI-9: Chl a316→PsaO: Chl a201	15.7	Lumenal
10-O (L)	ACPI-10: Chl a312→PsaO: Chl a201	18.3	Lumenal
10-O (L)	ACPI-10: Chl a311→PsaO: Chl a202	17.5	Lumenal
10-O (S)	ACPI-10: Chl a305→PsaO: Chl a203	21.0	Stromal
11-K (L)	ACPI-11: Chl a312→PsaK: Chl a101	19.0	Lumenal
11-O (S)	ACPI-11: Chl a306→PsaO: Chl a203	21.4	Stromal

Supplemental Table S6. Excitation energy transfer (EET) pathways from inner ACPIs to the PSI core.

Stromal EET	Lumenal EET
ACPI-1: Chl a306→PsaA: Chl a821	ACPI-1: Chl a312→PsaK: Chl a102
ACPI-2: Chl a305→PsaA: Chl a809	ACPI-2: Chl a312→PsaA: Chl a817
ACPI-3: Chl a306→PsaF: Chl a302	ACPI-3: Chl a312→PsaJ: Chl a101
ACPI-4: Chl a305→PsaR: Chl a201	ACPI-4: Chl a312→PsaF: Chl a303
ACPI-5: Chl a305→PsaR: Chl a201	ACPI-6: Chl a311→PsaB: Chl a814
ACPI-6: Chl a305→PsaB: Chl a820	ACPI-7: Chl a312→PsaB: Chl a841
ACPI-7: Chl a306→PsaB: Chl a811	ACPI-8: Chl a309→PsaI: Chl a102
ACPI-8: Chl a306→PsaB: Chl a803	ACPI-9: Chl a316→PsaO: Chl a201
ACPI-9: Chl a306→PsaL: Chl a202	ACPI-10: Chl a312→PsaO: Chl a201
ACPI-10: Chl a305→PsaO: Chl a203	ACPI-10: Chl a311→PsaO: Chl a202
ACPI-11: Chl a306→PsaO: Chl a203	ACPI-11: Chl a312→PsaK: Chl a101
